# Colloidal-ALD-Grown Metal Oxide Shells Enable the Synthesis of Photoactive Ligand/Nanocrystal Composite Materials

**DOI:** 10.1021/jacs.3c01439

**Published:** 2023-03-30

**Authors:** Philippe B. Green, Ona Segura Lecina, Petru P. Albertini, Anna Loiudice, Raffaella Buonsanti

**Affiliations:** † Laboratory of Nanochemistry for Energy Research, Institute of Chemical Sciences and Engineering, Ecole Politechnique Fédérale de Lausanne, Sion, CH-1950, Switzerland

## Abstract

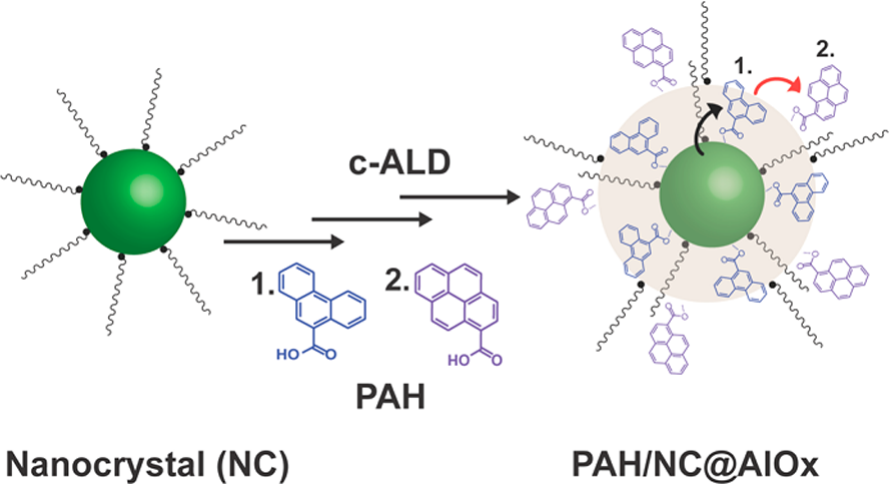

Colloidal nanocrystals (NCs) are ideal materials for a variety of applications and devices, which span from catalysis and optoelectronics to biological imaging. Organic chromophores are often combined with NCs as photoactive ligands to expand the functionality of NCs or to achieve optimal device performance. The most common methodology to introduce these chromophores involves ligand exchange procedures. Despite their ubiquitous nature, ligand exchanges suffer from a few limitations, which include reversible binding, restricted access to binding sites, and the need for purification of the samples, which can result in loss of colloidal stability. Herein, we propose a methodology to bypass these inherent issues of ligand exchange through the growth of an amorphous alumina shell by colloidal atomic layer deposition (c-ALD). We demonstrate that c-ALD creates colloidally stable composite materials, which comprise NCs and organic chromophores as photoactive ligands, by trapping the chromophores around the NC core. As representative examples, we functionalize semiconductor NCs, which include PbS, CsPbBr_3_, CuInS_2_, Cu_2–*x*_X, and lanthanide-based upconverting NCs, with polyaromatic hydrocarbons (PAH) ligands. Finally, we prove that triplet energy transfer occurs through the shell and we realize the assembly of a triplet exciton funnel structure, which cannot be obtained via conventional ligand exchange procedures. The formation of these organic/inorganic hybrid shells promises to synergistically boost catalytic and multiexcitonic processes while endowing enhanced stability to the NC core.

## Introduction

Colloidal nanocrystals (NCs) are employed in applications ranging from optoelectronics to biomedicine, which benefit from their size-dependent properties and tunable surface chemistry.^1,2^ The as-synthesized NCs include a crystalline inorganic core passivated by an organic ligand shell, which confers colloidal stability and prevents irreversible aggregation.^3^ However, these native ligands are not electronically conductive; thus they hinder energy and charge transport in or out of the NCs, which is essential in assembling viable NC-based catalysts or optoelectronic devices.^4,5^ Consequently, methods of exchanging the native ligands with electronically or optically active alternatives have been sought after over the years.^4,6−8^

A vast library of ligand exchange protocols has been developed and tailored for a multitude of applications. For example, NC-based photovoltaics benefit from the introduction of ligands with short alkyl chains or halide ligands on the NC surface, because these ligands optimize the NC packing density and favor efficient charge transport.^4,5^ Further, synergistic enhancement of heterogeneous catalysts has been attained by grafting organic species or organometallic complexes to the NC surface.^9−12^ Finally, NCs functionalized with polyaromatic hydrocarbon (PAH) ligands have emerged as excellent triplet exciton based sensitizers with applications in photocatalysis,^7,13−16^ optoelelctronics,^17,18^ optogenetics,^19,20^ and bioimaging.^21−23^

When the goal is to design and assemble structures consisting of NCs and chromophores capable of efficiently guiding the flow of energy or carriers between the two constituting units, the chromophores must be immobilized with an optimal density and in close proximity of the NC surface.^15,24−32^ To this end, ligand exchanges have been used to successfully replace a portion of the native synthetic ligands with the desired chromophore in a one-to-one swap, a process that is mostly driven by mass action.^29,33,34^ Despite being quite practical, these ligand exchanges present several limitations: (1) they require excess of the chromophore, which is often limited in supply; (2) excess chromophore passivation can hamper colloidal stability; (3) NCs need to be purified after the exchange, which leads to further loss of chromophore coverage and colloidal stability;^35^ (4) unpurified samples can suffer from detrimental absorptive screening by free ligands and have limited processability;^36^ (5) the introduction of multiple types of ligands results in their inherent competition for the limited available binding sites;^37^ and (6) sterically hindered ligands have a restricted capability of exchange.^33,34^ Consequently, an alternative methodology to ligand exchanges which traps functional organic molecules on the NC surface and/or in its close proximity would be beneficial to extend and enhance the applicability of NCs.

Established methodologies based on silica, aerogel, or polymer coatings on NCs have been employed to create a variety of composite materials.^38−42^ These methods confer stability underharsh environments to the NCs and the possibility to endow the NCs with new functionalities, including coupling them with chromophores.^38−42^ Nonetheless, such methods do not allow for the creation of thin shells, and, thus, they are impractical for applications that strongly rely on physical proximity. Hence, synthetic methods that offer sub-nanometric tunability of NC coatings would be beneficial.

Herein, we propose the synthesis of a hybrid organic/inorganic shell as a strategy to efficiently immobilize organic chromophores in close proximity of the NC surface and with tunable density. Recently, the development of colloidal atomic layer deposition (c-ALD) of metal oxides, such as amorphous alumina, was demonstrated to tightly anchor the otherwise dynamic ligands to the NC surface and, thus, to confer the inorganic NC core enhanced stability.^43,44^ Further, work by Segura Lecina et al. demonstrated that c-ALD of alumina is governed via ligand–precursor interactions, which result in a hybrid organic/inorganic shell.^45^ These observations suggest that introducing ligands that interact with the c-ALD precursor could promote their efficient incorporation within the hybrid oxide shell. This strategy would increase the ligand coverage without requiring a ligand exchange process. These observations are particularly appealing if functional ligands were involved in such ligand–precursor interactions and embedded in the alumina shell.

In this work, we grow alumina shells by c-ALD on semiconductor NCs of six different compositions to create organic/inorganic hybrid materials with PAH ligands. In particular, we choose to incorporate 9-anthracene carboxylic acid (9-ACA), 9-phenanthrene carboxylic acid (9-PTA), and 1-pyrene carboxylic acid (1-PCA) as bulky photoactive chromophores because of their demonstrated applications in incoherent photon conversion and photocatalysis.^15,24,25,29,46^ We use c-ALD on three types of surfaces: carboxylated (PbS, NaYF_4_, and NaGdF_4_:Yb), thiolated (Cu_2–*x*_S and CuInS_2_), and oleylamonium (halide and carboxylate)-passivated (CsPbBr_3_). We selected these NCs, as they have historically benefited from being coupled to photoactive ligands. For instance, PbS, CuInS_2_, and CsPbBr_3_ passivated by PAH ligands have found great use in incoherent photon conversion and photocatalyst.^15,17,24,47−49^ The coupling of PAH ligands with lanthanide-based upconverting nanoparticles (UCNPs) can drastically increase the absorption cross-section of the chromophore/NC hybrids, resulting in a significant enhancement of the upconverted emission intensity.^21,22,36^ Finally, plasmonic NCs can also be coupled to chromophores, which benefits photo and electrocatalytic applications.^9−11^

Via these examples, we illustrate that c-ALD of alumina is a simple and generalizable methodology that ensures that all ligands are embedded in a single colloidally stable hybrid structure, which possess great processability. We highlight the possibility of assembling complex structures composed of multiple ligands on a variety of NC surface types. Finally, we demonstrate a proof-of-concept surface-bound triplet exciton funnel, a structure that is not accessible via conventional ligand exchange processes.

## Results and Discussion

### Synthesis and Characterization of the PAH/NC@AlO_*x*_ Composite Materials

We synthesized NCs with six different compositions, which include oleate-passivated PbS,^50^ NaYF_4_,^21^ and NaGdF_4_:Yb,^21^ oleylamonium (bromide and oleate)-passivated CsPbBr_3_ (both quantum confined^51^ 5.1 ± 0.6 nm and unconfined^52^ 11 ± 2 nm NCs), and thiol-passivated Cu_2–*x*_S^53^ and CuInS_2_^54^ NCs, following established protocols (see experimental part in the SI). A detailed characterization of these NCs is reported in the SI (Figures S1–S9, Table S1). We grew the c-ALD alumina shell according to the previously developed procedure, which is based on alternating additions of trimethylaluminum (TMA) and O_2_ gas.^44^ We confirmed the presence of alumina shells grown by c-ALD on all the NCs using a combination of FTIR and XPS (Figures S10–S17).

**Figure 1:**
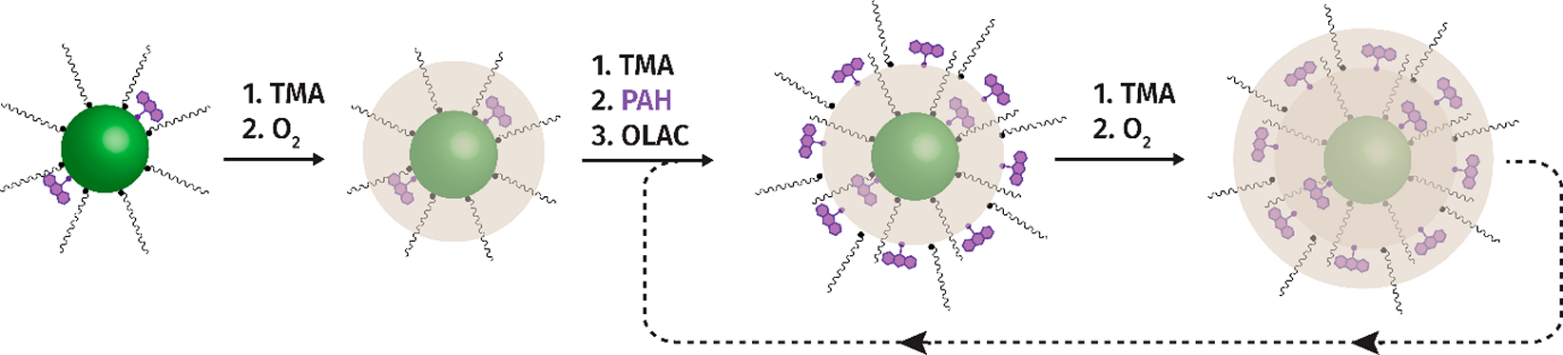
Schematic illustration of the c-ALD process to synthesize the composite materials including a NC core and a PAH-alumina hybrid shell (PAH/NC@AlO_*x*_). As an initial step TMA is titrated to a solution of NCs. Then, the shell growth is initiated through cyclic additions of TMA and O_2_. As the shell grows, PAH and OLAC ligands are added every two to four TMA/O_2_ cycles. This process yields a colloidally stable composite material consisting of a NC core and an organic/inorganic shell with tunable loading of PAH molecules per NC.

Having demonstrated that alumina shells can be grown, we went beyond the previously established procedure and set out to assemble hybrid structures composed of the NC core passivated by an alumina shell with embedded PAH ligands (Figure 1), which we will refer to as PAH/NC@AlO_*x*_. In brief (details reported in the SI), we initiate the alumina growth with three c-ALD cycles consisting in alternating TMA/O_2_ injections. An initial ligand exchange procedure to introduce some PAH ligands on the surface might be performed or not, depending on the system of interest (see details in the experimental section in the SI). We continue the growth by alternating injections of oleic acid (OLAC) and the desired PAH ligand every two to four TMA/O_2_ cycles. The optimized solution of the injected ligands includes a 1:1 OLAC:PAH molar ratio for a total of around 30 molecules/NC for each ligand addition.

**Figure 2:**
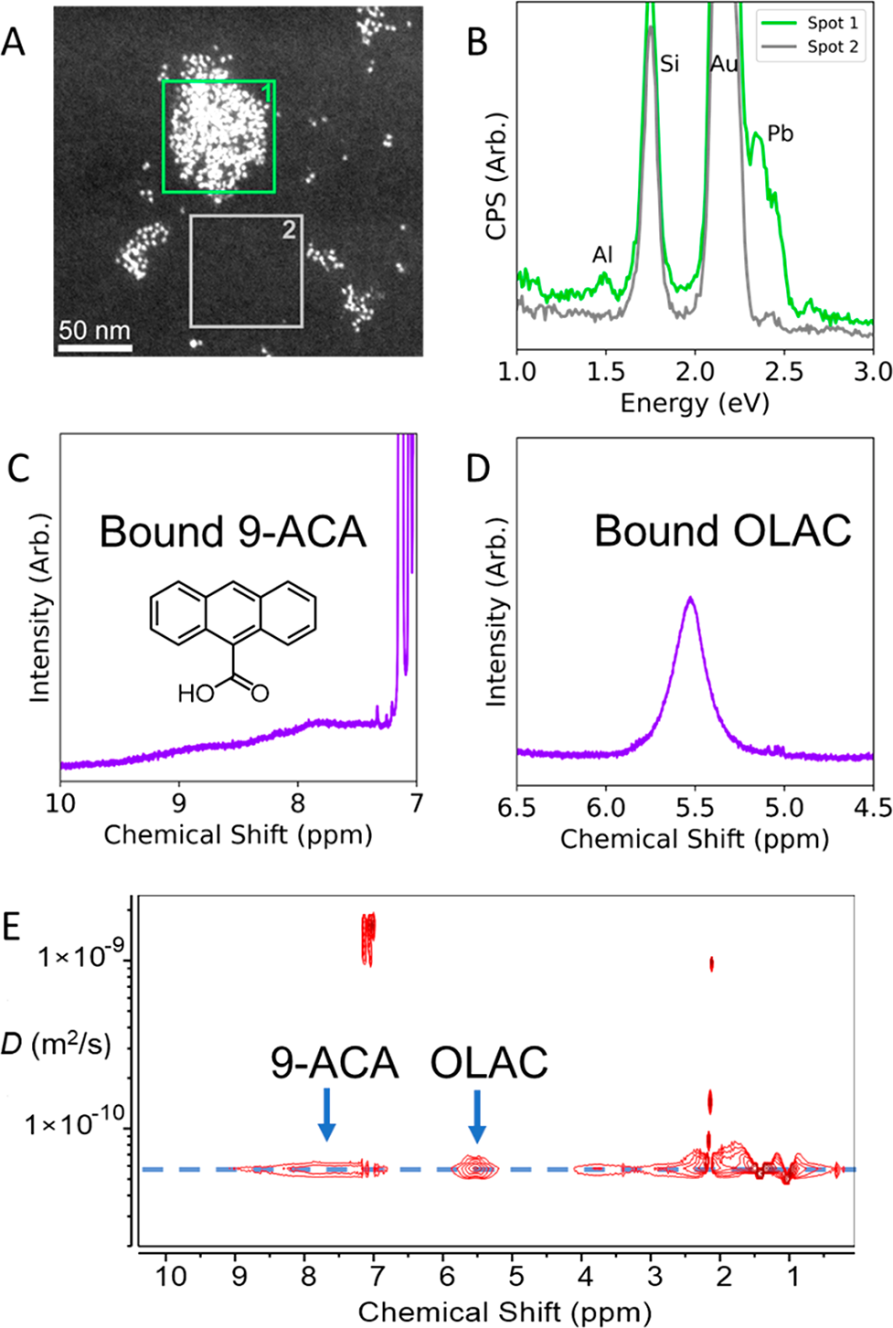
Characterization of the 9-ACA/PbS@AlO_*x*_ composite materials. (A) Representative HAADF-STEM image of the composite material and (B) corresponding EDX spectra on locations with and without PbS NCs. Aluminum is colocalized with Pb, confirming the formation of an alumina shell. (C, D) Representative ^1^H NMR spectra of the composite material showing broad resonances for the 9-ACA (C) and OLAC (D) protons and no narrow resonances, which suggest that all ligands are in a bound state. (E) Representative DOSY spectrum, which confirms that 9-ACA, OLAC, and the PbS NCs diffuse as a unit through the presence of a single diffusion coefficient for all ligands. These results were obtained for a sample prepared according to the synthetic protocol: 3×[TMA/O_2_] + 6×[TMA/L/TMA/O_2_] + 3×[TMA/O_2_] (L = 9-ACA+OLAC in a 1:1 molar ratio), which yields ∼90 9-ACA molecules/NC.

We started our investigation by incorporating 9-ACA within the alumina shell around PbS NCs, which we refer to as 9-ACA/PbS@AlO_*x*_. Figure 2 provides an overview of the obtained results. First, transmission electron microscopy (TEM) confirmed that the size and morphology of the NCs is preserved after the process and homogeneously nucleated alumina was not observed (Figure S18). In agreement, high-angle annular dark field scanning transmission electron microscopy (HAADF-STEM) imaging combined with energy dispersive X-ray (EDX) spectroscopy (Figure 2A,B) reveals that Al and Pb are spatially correlated, suggesting that alumina is deposited on the NC surface.

^1^H NMR measurements show a large convoluted signal arising from the 9-ACA embedded in the shell and, thus, in a bound state, between 6.5 and 10 ppm (Figures 2C, S19) and a single broad feature associated with the oleate ligands in a bound state at 5.5 ppm after the c-ALD process (Figures 2D, S19). No contribution from free or dynamic ligands was detected (Figure S19). Diffusion-ordered NMR spectroscopy (DOSY) measurements indicate an equivalent diffusion coefficient for both 9-ACA and oleate ligands, suggesting that both are diffusing along with the NC core (Figure 2E). As the coexistence of similarly sized PbS NCs and alumina is unlikely, the latter not being detected by electron microscopy, the DOSY data are consistent with the absence of free ligands and freely nucleated alumina. Further, titrations with undec-10-enoic acid (UDA) confirmed that the 9-ACA ligands are trapped in the shell as 9-ACA was not displaced by the incoming UDA ligand (Figures S20, S21).

**Figure 3:**
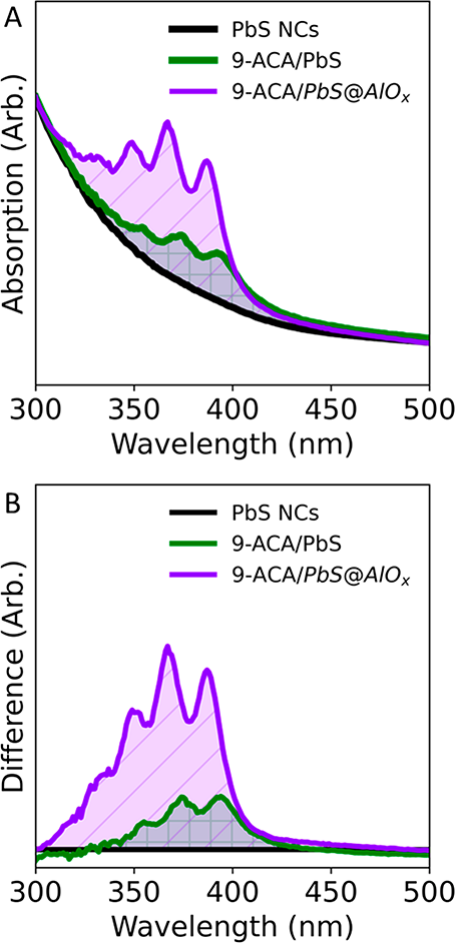
Optical properties of the 9-ACA/PbS@AlO_*x*_ compared with conventional ligand-exchanged 9-ACA/PbS NCs. (A) Optical absorption and (B) difference spectra of the as-synthesized PbS NCs (black), ligand-exchanged 9-ACA/PbS NCs (green), and 9-ACA/PbS@AlO_*x*_ (purple). Looking at the region where 9-ACA is optically active, the presence of 9-ACA atop the profile of the as-synthesized PbS NCs is clearly visible. The subtraction of the as-synthesized PbS spectrum to the others allowed us to calculate the ligand density per NCs, which is ∼30 9-ACA/NC in the ligand-exchanged 9-ACA/PbS NCs and ∼90 9-ACA/NC in the 9-ACA/PbS@AlO_*x*_.

We then thought to quantitatively compare and contrast samples obtained via c-ALD and by conventional ligand exchange. The ligand-exchanged samples are referred to as PAH/NC and were obtained by first adding a large excess of PAH to the NC suspension followed by antisolvent purification to ensure that all subsequent comparisons only regard bound ligands. Figure 3 compares the optical absorption of 9-ACA/PbS@AlO_*x*_ and 9-ACA/PbS obtained via X-for-X exchange, where carboxylates are swapped with carboxylates. The absorption spectrum of the 9-ACA/PbS@AlO_*x*_ shows a larger contribution from 9-ACA compared to that of the 9-ACA/PbS NCs (Figure 3A), which indicates higher loading. We calculated the number of the 9-ACA molecules embedded in the shell and, thus, in a bound state, by subtracting the optical absorption of the as-synthesized NCs from that of the functionalized samples (Figure 3B). In the illustrated example, we estimated the amount of 9-ACA in a bound state to be approximately 30 molecules/NC and 90 molecules/NC in 9-ACA/PbS and 9-ACA/PbS@AlO_*x*_, respectively. Irrespective of the number of 9-ACA molecules introduced during the ligand exchange (which were 380 molecules/NC for the selected example), we could not achieve a higher loading on the native surface (Figures S22 and S23). This limited loading by conventional X-for-X exchange, despite the large excess initially employed, reflects the limited availability of binding sites compatible with the presence of the bulky PAH ligands on the native surface.^33,34^ In contrast, these optical data demonstrate that c-ALD is a method that creates new binding sites unencumbered by the native surface and allowing for the incorporation of additional functional ligands, thus bypassing the thresholded incorporation obtained by X-for-X exchange.

**Figure 4:**
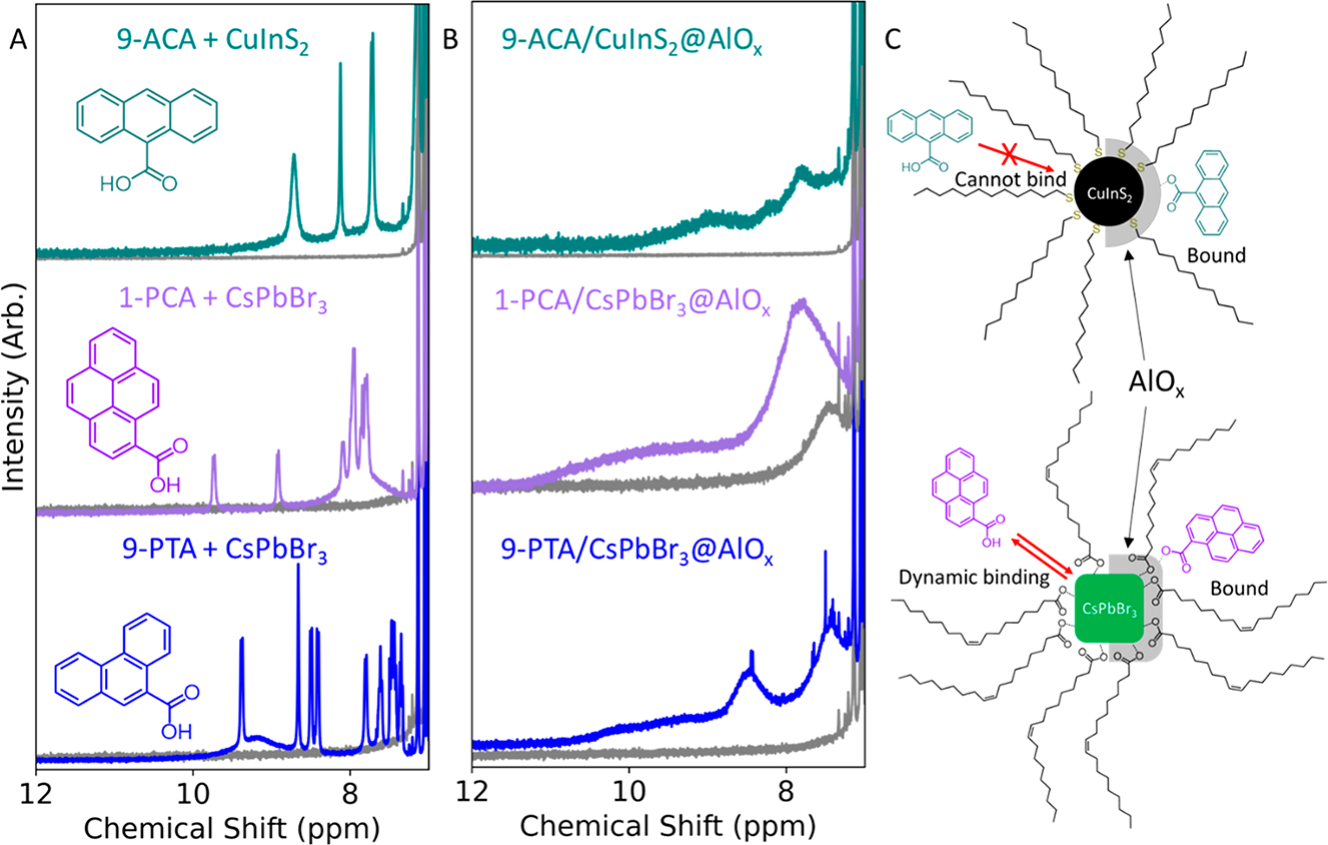
Generality of the PAH/NC@AlO_*x*_ synthesis. (A) ^1^H NMR of CsPbBr_3_ NCs with 1-PCA and 9-PTA ligands and CuInS_2_ with 9-ACA. Herein the ligands are added to solution without purification (see Figure 5). The narrow line widths indicate that the PAH ligands are either in a dynamic exchange (1-PCA or 9-PTA with CsPbBr_3_) or noninteracting with the NC surface (9-ACA with CuInS_2_). The as-synthesized samples are shown in gray. (B) ^1^H NMR of 1-PCA/CsPbBr_3_@AlO_*x*_, 9-PTA/CsPbBr_3_@AlO_*x*_, and 9-ACA/CuInS_2_@AlO_*x*_; the broadened NMR line widths indicate that all the PAH ligands added are embedded in the system. The as-synthesized samples are shown in gray. (C) Schematic representation of the introduction of PAH ligands on the as-synthesized and oxide shelled NCs. In CuInS_2_ NCs, 9-ACA cannot exchange because of the comparatively stronger bonds of the native thiolates. The growth of an alumina shell generates new sites that enable the binding of the 9-ACA. In CsPbBr_3_ NCs, the ligand shell is highly dynamic, resulting in a labile interaction of the introduced ligands with the surface. The growth of an alumina shell circumvents the inherent dynamicity and creates a sample with fully embedded PAH ligands in a bound state. The surface chemistry depiction is simplified in the case of the CsPbBr_3_ NCs as the halide and ammonium ligands, which are also present, are not displayed for clarity.

Having assessed the validity of the proposed methodology to create 9-ACA/PbS@AlO_*x*_ composite materials, we performed similar experiments on CsPbBr_3_, CuInS_2_, Cu_2–*x*_S, NaGdF_4_:Yb, and NaYF_4_ to expand the library of composite materials and surface chemistries. We also included 1-PCA and 9-PTA as the PAH ligands. Electron microscopy confirmed that the NC morphology was mainly preserved, and no alumina aggregates were observed, suggesting the absence of homogeneously nucleated alumina and the deposition of the alumina on the NCs (Figures S24 and S25). Figure 4 showcases the most representative examples and reports the ^1^H NMR spectra of 1-PCA and 9-PTA added to 5.1 nm CsPbBr_3_ NCs and of 9-ACA added to CuInS_2_ NCs without purification (PAH+NC, Figure 4A) and those after c-ALD (PAH/NC@ALO_*x*_, Figure 4B). The narrow ^1^H NMR line widths of the 1-PCA and 9-PTA+CsPbBr_3_ NCs are consistent with the intrinsic high ligand dynamicity of the system, indicating a dynamic exchange on the surface (Figures 4A, S26–S29). In contrast, c-ALD resulted in the complete binding of the photoactive ligands to the surface, which is revealed by the much broader ligand line widths in the 1-PCA/CsPbBr_3_@AlO_*x*_ and 9-PTA/CsPbBr_3_@AlO_*x*_ samples (Figure 4B, S30 and S31). These experiments were repeated with the 11 nm CsPbBr_3_ NCs using 9-ACA as the PAH ligand and revealed an analogous behavior (Figures S32–S34).

The ^1^H NMR line widths of the 9-ACA remain narrow when these ligands are added to a solution of CuInS_2_ NCs (Figures 4A, S35–S37). This result is consistent with the fact that carboxylates, such as 9-ACA, cannot substitute the thiolate ligands on CuInS_2_ and Cu_2–*x*_S NCs via conventional ligand exchanges because of the weaker binding of carboxylates compared to thiolates on the surface of these NCs.^37,55^ Instead, the broad NMR line width in 9-ACA/CuInS_2_@AlO_*x*_ indicates that 9-ACA is in a bound state after c-ALD (Figures 4B, S38). Similarly, we could also synthesize 9-ACA/Cu_2–*x*_S@AlO_*x*_ composite materials (Figure S33). DOSY measurements revealed a similar diffusion coefficient for the original ligands (dithiothreitol, DDT) and for the OLAC and PAH ligands added during the c-ALD, which provides additional confirmation of their incorporation into the alumina matrix (Figures S38 and S39). ^1^H NMR experiments on NaYF_4_ NCs demonstrated the 9-ACA can be fully integrated in the ligand shell through c-ALD in 9-ACA/NaYF_4_@AlO_*x*_ (Figures S40 and S41). For NaGdF_4_:Yb NCs, ^1^H NMR experiments were not possible; consequently we could not assess the coordination state of the ligands. However, given the similar synthetic routes and surface chemistry of NaYF_4_ and NaGdF_4_:Yb, we expect that our scheme can also be transferred to lanthanide-based upconversion NCs.^21^

A schematic representation highlights the major differences between the incorporation of PAH ligands by conventional ligand exchange and by the proposed methodology based on c-ALD (Figure 4C). Specifically, the PAH ligands showcased in this figure are incompatible with ligand exchange procedures on CuInS_2_, due to strong binding of the native ligands, and are not optimal on CsPbBr_3_ NCs, due to dynamic binding of carboxylates on their surface. Yet, c-ALD bypasses these issues by creating new biding sites and localizes all PAH ligands within an alumina shell around the NC core.

**Figure 5:**
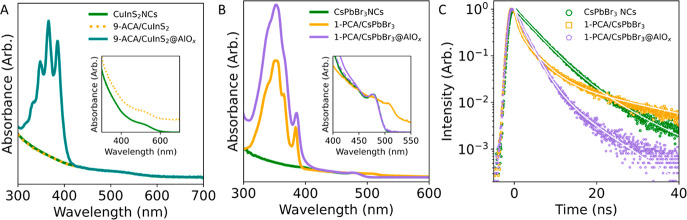
Optical properties of PAH/NC@AlO_*x*_ compared with conventional ligand-exchanged PAH/NCs. (A) Optical absorption spectra of the as-synthesized DDT-passivated CuInS_2_ (green), ligand-exchanged 9-ACA/CuInS_2_ (dotted orange), and 9-ACA/CuInS_2_@AlO_*x*_ with ∼60 9-ACA embedded in the shell per NC (teal). The inset highlights the absence of any 9-ACA in 9-ACA/CuInS_2_ compared to the as-synthesized CuInS_2_ NCs. (B) Optical absorption spectra of the as-synthesized 5.1 nm CsPbBr_3_ NCs (green), ligand-exchanged 1-PCA/CsPbBr_3_ NCs (orange), and 1-PCA CsPbBr_3_@AlO_*x*_ with ∼100 1-PCA embedded in the shell per NC (purple). The inset highlights the red-shift of the absorption edge and loss of the confinement for the ligand-exchanged 1-PCA/CsPbBr_3_ NCs which underwent antisolvent purification. (C) TRPL decay of the samples shown in B. The faster decay of 1-PCA/CsPbBr_3_@AlO_*x*_ (purple) compared to the as-synthesized sample (green) is attributed to triplet energy transfer. In contrast, a longer lifetime component appears when using antisolvent purification to obtain bound chromophores (orange), which is consistent with the creation of trap states.

Then, we moved to compare the optical properties of the PAH/NC@AlO_*x*_ with the ligand-exchanged PAH/NCs. Figure 5 reports the data for the same samples shown in Figure 4, plus those further purified via antisolvent washing. The addition of 9-ACA to DDT-passivated CuInS_2_ NCs does not form 9-ACA/CuInS_2_ NCs. In fact, there is no optical signature of 9-ACA upon antisolvent purification, which was performed to ensure only bound ligands remained present. This result suggests that introducing 9-ACA to the native surface of CuInS_2_ NCs via ligand exchange is highly unlikely (Figures 5A, S42). In contrast, the characteristic 9-ACA absorption features are evident in 9-ACA/CuInS_2_@AlO_*x*_ (Figure 5A), consistently with the indication of bond ligands provided by ^1^H NMR (Figure 4B). We estimated approximately ∼60 9-ACA molecules embedded in the shell per NC in the sample shown in Figure 5A. We observed a similar behavior for Cu_2–*x*_S NCs (Figure S38).

As for the 1-PCA on CsPbBr_3_ (5.1 nm), some binding of the ligand is observed after purification (Figures S43 and S44). As a matter of fact, the optical features of the 1-PCA are present in 1-PCA/CsPbBr_3_ (Figure 5B). However, a portion of the 1-PCA remained free and the band edge absorption (inset in Figure 5B) underwent a red-shift, which indicates the formation of larger undesired NCs. On the contrary, the absorption of 1-PCA/CsPbBr_3_@AlO_*x*_ clearly indicates the presence of the ligands (Figure 5B), consistently with their bound state evidenced by NMR (Figure 4B). We calculate that this sample contains approximately ∼100 1-PCA molecules per NC. Further, the band edge feature remains intact, suggesting that c-ALD is nondestructive. Time-resolved photoluminescence (TRPL) shows faster decay in the 1-PCA/CsPbBr_3_@AlO_*x*_ compared to the as-synthesized CsPbBr_3_ NCs (Figure 5C). This faster decay can be attributed to triplet energy transfer (Figure S45). In contrast, the PL decay of the ligand-exchanged 1-PCA/CsPbBr_3_ upon purification shows a longer lifetime component, which is representative of the creation of trap states. This formation of trap states is indicative of ligand stripping and sample degradation by the antisolvent, consistent with the literature.^35,56^ Thus, c-ALD enables the assembly of CsPbBr_3_ NCs with photoactive ligands solely in a bound state embedded in the shell without the need for destructive purification steps, which is crucial for these samples.

**Figure 6:**
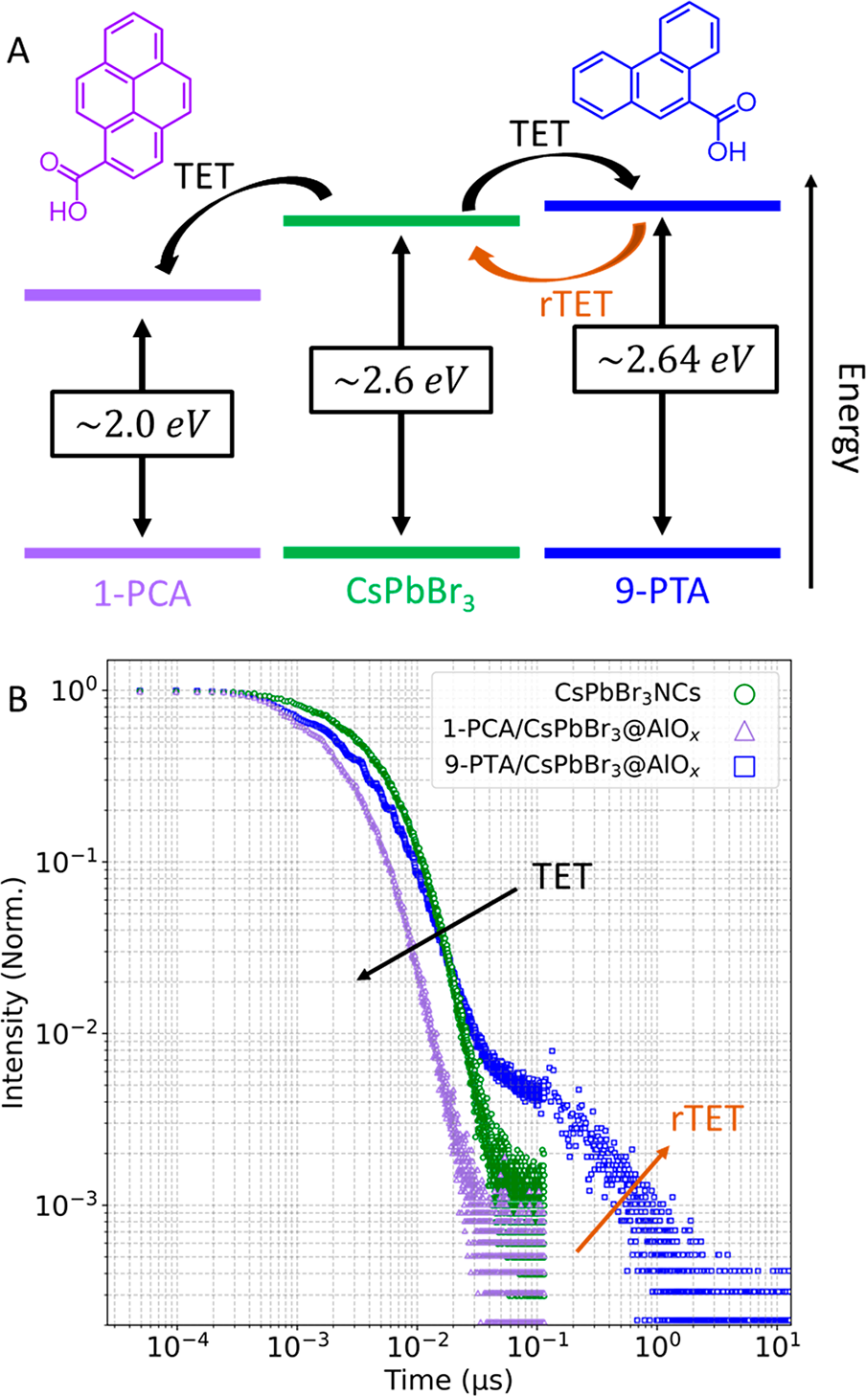
Time-resolved photoluminescent decay of 1-PCA/CsPbBr_**3**_@AlO_*x*_ and 9-PCA/CsPbBr_**3**_@AlO_*x*_. (A) Energy levels of the 5.1 nm CsPbBr_3_ and triplet excited states of 1-PCA and 9-PTA. Highlighted are the various energy transfer routes possible: direct triplet energy transfer (TET) from the NC to 1-PCA and 9-PTA; reverse TET (rTET) from 9-PTA to the NC. (B) TRPL decay of a sample with ∼100 PAH per NC. When incorporating either 1-PCA (purple) or 9-PTA (blue) into the alumina shell, TET is observed from both ligands and rTET is seen from 9-PTA.

**Figure 7:**
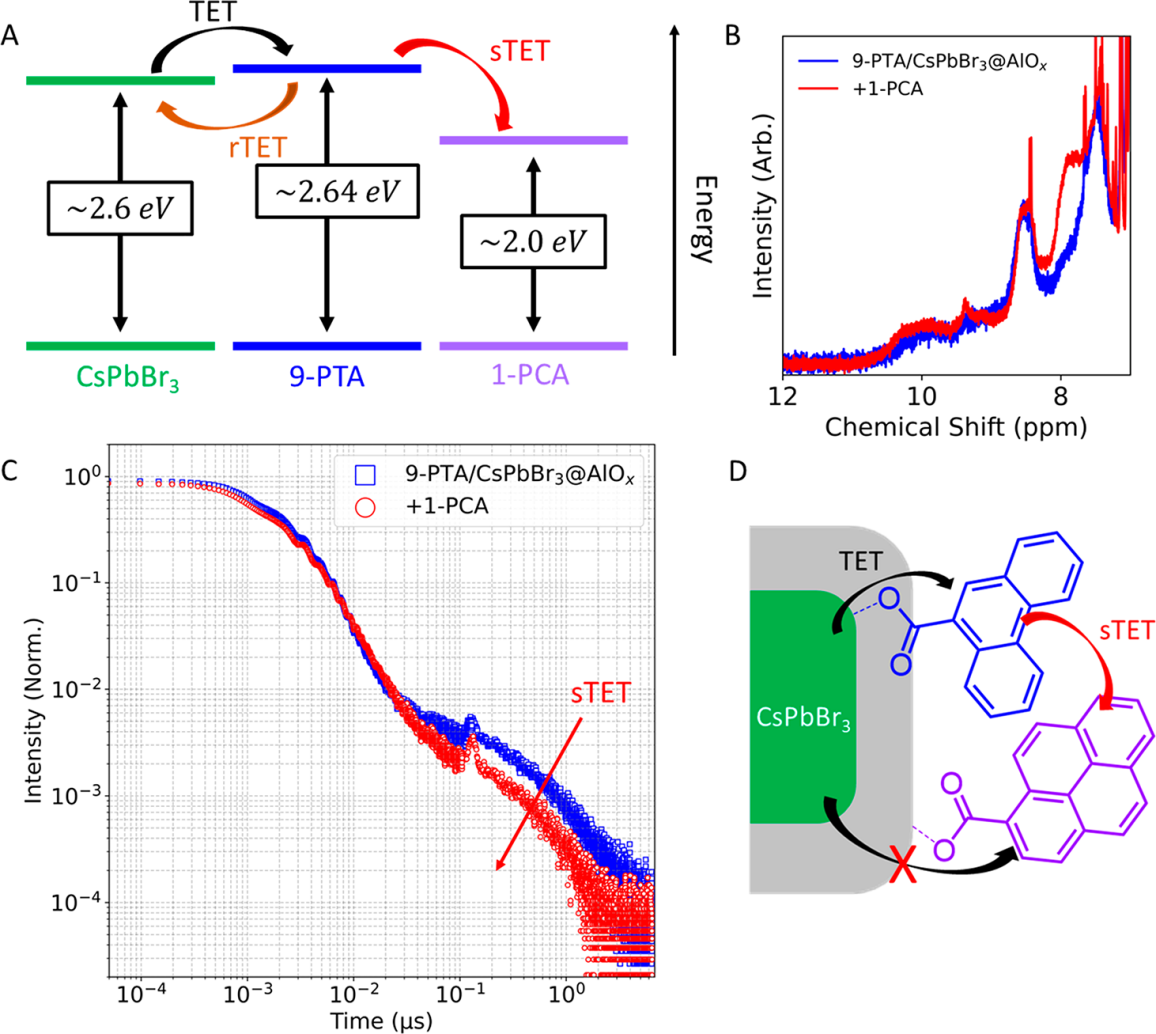
Assembly of a PAH/CsPbBr_**3**_@AlO_*x*_ composite with the function of a triplet exciton funnel. (A) Schematics of the energetics involved in assembly of a triplet exciton funnel away from CsPbBr_3_ NCs, which involves a secondary TET (sTET) step from 9-PTA to 1-PCA. (B) ^1^H NMR of the NCs with ∼120 9-PTA molecules introduced within the alumina shell around a 5.1 nm CsPbBr_3_ NC (blue) and after the addition of ∼40 1-PCA molecules per NC (red). 1-PCA does not displace 9-PTA, and both ligands are in a bound state. (C) Time-resolved photoluminescence of the selective excitation of the assembled NC structure with embedded 9-PTA, which displays the expected TET and rTET (blue). The addition of 1-PCA quenches the reverse TET (rTET) suggestive of sTET. No direct TET from the NC to 1-PCA is observed, confirming the formation of a triplet exciton funnel. (D) Schematic of the proposed structure of the surface-bound exciton funnel.

Having confirmed that the c-ALD-grown alumina shell generates samples with PAH ligands solely in a bound state with a higher loading than what can be obtained via conventional ligand exchange processes, we moved toward demonstrating the functionality of the newly synthesized PAH/NC@AlO_*x*_ composite materials for energy transfer (Figures 6 and 7). First, we studied the possibility to transfer energy to and from the NC core via the PAH embedded in the shell by triplet energy transfer (TET). Particularly, we were concerned that the shell, while incorporating the ligands around the NC, would prevent efficient TET due to physical distancing between the two units. We elected to combine the quantum confined 5.1 nm CsPbBr_3_ NCs (*E*_g_: 2.6 eV) and 1-PCA (triplet energy: 2 eV) and 9-PTA (triplet energy: 2.64) based on their energy levels (Figure 6A).^25,29^

We monitored the sensitization of triplet excitons by recording the TRPL when selectively exciting the NCs with 450 nm irradiation. We observe quenching of the NC photoluminescence by TRPL as a change in the average PL lifetime from *τ*_ave_ = 4.1 ns to 2.0 ns when 1-PCA is loaded in the alumina shell (Figure 6B). This quenching is not associated with the alumina shell, as CsPbBr_3_ NCs upon which a similar shell was grown but without 1-PCA possess a *τ*_ave_ of 3.7 ns (Figure S46). Thus, we conclude that TET does occur in the 1-PCA/CsPbBr_3_@AlO_*x*_. We note that the quenching is not as efficient as when directly having 1-PCA bound to the NC surface (Figure S47, Table S2), which is expected as the shell acts as a spacer. However, our composite is a more efficient quencher when solely bound ligands are required (Figure 5C). We also tested TET from CsPbBr_3_ to 9-PTA in 9-PTA/CsPbBr_3_@AlO_*x*_. As TET from the NC to 9-PTA is endothermic, a reverse TET (rTET) channel is expected from 9-PTA back to the NC (Figure S48).^29,46^ The TRPL measurements support this hypothesis, as the data show an early time quenching followed by a microsecond-scale component associated with rTET (Figure 6B).^46^ The observation that TET and rTET take place in our system is consistent with at least a fraction of the PAH molecules being in close proximity of the NC surface and, possibly, separated by only a thin layer of alumina (∼0.3 nm),^44^ a distance that is comparable to previous TET distance dependent studies.^57^ Altogether these experiments show that TET from or to the NC is possible through the shell, and further optimization should be possible in future work thanks to the tunability of the entire process.

Finally, given that we can position a variety of PAH ligands on a multitude of nanocrystalline cores while ensuring that all the ligands are in a bound state embedded in the shell, we sought to assemble a composite material that leveraged the newly gained complexity. To this aim, we assembled a structure capable of funneling triplet excitons away from the NC core beyond a single PAH ligand, while being fully contained within the vicinity of the NC. Such a structure cannot be achieved via conventional surface functionalization of native NCs as the sensitized triplet exciton will always be located on the PAH immediately adjacent to the NC core. Such a proximity has been noted to reduce the triplet exciton lifetime, limiting following exciton transfer steps essential for photochemistry.^57^ Further, the proximity of the PAH to the NC surface limits the possibility of triplet fusion upconversion within the ligand shell, as the generated singlet exciton can readily under go Förster resonance energy transfer (FRET) back to the NC.^57−59^ Circumventing these main limitation could provide a platform to better employ triplet excitons in photocatalysis and to develop triplet fusion upconversion schemes that do not rely on molecular diffusion. Our proposed scheme employs c-ALD to stagger different PAH ligands within the shell. This process would provide a spatial and energetic continuum of PAH ligands with properly chosen energy levels that are gradually distanced from the NC core. Consequently, we hoped to assemble a shell that would extract triplet excitons from the NC and funnel them away from the NC core toward an otherwise inaccessible shell surface.

To demonstrate the formation of a surface-bound triplet exciton funnel, we opted to assemble a composite material with 9-PTA in proximity to the NC surface and 1-PCA on the shell surface. We selected the 9-PTA and 1-PCA pair, as the rTET afforded by 9-PTA provides a direct spectroscopic probe by TRPL of a secondary TET (sTET) (Figure 7A). This sTET would be manifested through the quenching of rTET (Figure 7A). Consequently, this proof-of-concept funnel is not designed to be the most efficient but to be directly observable by TRPL. We grew an alumina shell that incorporated 9-PTA. ^1^H NMR confirmed that all 9-PTA molecules are in a bound state (Figure 7B). The TRPL traces display the two expected triplet energy transfer steps (endothermic TET from the NC to 9-PTA and an rTET back to the NC) (Figures 7C, S49). Upon addition of 1-PCA, ^1^H NMR confirmed that all added ligands are in a bound state (Figure 7B), while a similar rate of TET and a quenching of the rTET are observed (Figure 7C). The average PL lifetime, calculated by a Riemann sum, goes from *τ*_ave_ = 0.44 μs to 0.17 μs. We exclude the quenching of rTET being due to direct TET to 1-PCA, as control experiments involving the addition of 1-PCA on NC initially passivated by a similarly grown shell did not show any quenching of the CsPbBr_3_ NCs. These results confirmed that 1-PCA is spatially distant from the core and unable to directly extract a triplet from the NC, but in a close enough proximity to 9-PTA to promote sTET (Figure S50). This experiment demonstrates that sTET to 1-PCA occurs within the vicinity of the NC surface. These observations combined with the localized positioning of 1-PCA to the shell surface suggest that a portion of the sensitized triplet excitons are funneled to the surface 1-PCA through 9-PTA (Figure 7D). Thus, c-ALD can be used to assemble complex structures capable of directing the flow of energy in the form of exciton funnels.

## Conclusion

In conclusion, we have synthesized ligand/NC composite materials by growing alumina shells embedding photoactive PAH molecules on a variety of nanocrystalline cores via c-ALD. c-ALD anchors these functional ligands in places and allows for the assembly of hybrid metal-oxide shells, which are capable of funneling energy from the NC core to the surface of the composite material. We expect this scheme to be extendable to other metal oxides and we believe that a variety of ligands with different binding head groups could be used to favor energy transfer. In addition, we consider that c-ALD could be leveraged in schemes where multiple functional ligands are introduced in the hybrid shell surrounding the NC core to create a variety of functional composite materials. Consequently, c-ALD emerges as a methodology to assemble colloidally stable complex hybrid structures that can find practical applications in incoherent photon conversion and photocatalysis and beyond.

## Data Availability

The data underlying this study are openly available in Zenodo at 10.5281/zenodo.7707974.

## Supporting Information Available

Additional experimental details, FTIR, XPS, NMR, TEM, UV–vis, PL emission spectra, and TRPL; further discussions on ligand exchanges (PDF)
