# Supplementary material for: Colloidal-ALD-Grown Metal Oxide Shells Enable the Synthesis of Photoactive Ligand/Nanocrystal Composite Materials
Source: J Am Chem Soc. 2023 Mar 30;145(14):8189–97. doi: 10.1021/jacs.3c01439 (PMC10103164; doi:10.1021/jacs.3c01439)
Supplement: Supplementary file 1 — ja3c01439_si_001.pdf [file ja3c01439_si_001.pdf]

Supporting information

Colloidal-ALD Grown Metal Oxide Shells Enable the Synthesis of Photoactive Ligand/ Nanocrystal Composite Materials

Philippe B. Green,[†] Ona Segura Lecina,[†] Petru P. Albertini,[†] Anna Loiudice[†] and Raffaella Buonsanti[†]*

[†]Laboratory of Nanochemistry for Energy Research, Institute of Chemical Sciences and Engineering, Ecole Politechnique Fédérale de Lausanne, Sion, CH-1950, Switzerland

*email: raffaella.buonsanti@epfl.ch

Material & Instruments

Chemicals:

All chemicals unless specified were purchased from Sigma-Aldrich. Lead Oxide (99.999%), bis(trimethylsilyl) sulfide (synthesis grade), cesium carbonate (99.9%), lead bromide (99.99%, Alfa Aesar), zinc bromide (99.999%), copper acetylacetonate (99.9%), copper (II) acetate (99.99%), indium acetate (99.99%), yttrium acetate hydrate (99.9%), gadolinium acetate hydrate (99.9%), ytterbium acetate hydrate (99.9%), sodium hydroxide (97%), ammonium fluoride (99.99%), 1-octadedene (ODE, Technical grade 90%), oleic acid (OLAC, Technical grade 90%), oleylamine (OLAM, technical grade 70%), 1-dodecanethiol (DDT, 98%), hexane (95%), octane (anhydrous ≥99%), acetone (anhydrous 99.8%, ACROS), ethyl acetate (anhydrous 99.8%), deuterated toluene (99%), trimethylaluminium (TMA, 98% Strem), 9-anthracene carboxylic acid (9-ACA, 99%), 1-pyrene carboxylic acid (1-PCA, 97%), 9-phenathrene carboxylic acid (9-PTA, 97% Fluorochem).

Instrumentation:

Optical absorption: Measured using a PerkinElmer Lambda 950 spectrophotometer, equipped with a deuterium (ultraviolet range) and tungsten lamp (visible and infrared range). A PMT and InGaAs detector was used.

Time resolved photoluminescence (TRPL): A Horiba Jobin Yvon Fluorolog-3 instrument was employed. A Horiba nanoLED with excitation wavelength of 450 nm was employed to selectively excite the $CsPbBr_3$ NCs.

Fourier transform infrared spectroscopy (FT-IR): FT-IR was carried out using an attenuated total reflectance (ATR) Perkinelmer Two spectrometer. The sample was directly drop-casted on the ATR plate from hexanes or octane.

Nuclear magnetic resonance (NMR): A Bruker Avance III 400 MHz spectrometer equipped with a 5mm BBFO Z-gradient SmartProbe was used. ^1H, DOSY and NOESY were acquired using standard pulse sequences from the Bruker library. Data was processed using Topspin and Mestrenova.

Transmission electron microscopy (TEM): A FEI Tecnai-spirit at 120 kV was employed. The sample was prepared by drop casting from hexane or octane on copper TEM grids. ImageJ was used to calculate the average NC size.

X-ray photoelectron spectroscopy (XPS): XPS was performed using a Kratos Analytical instrument equipped with a monochromated Kα X-ray line of an Al anode. The samples were prepared by drop casting the sample from hexane or octane on a clean silicon substrate. CasaXPS was used to analyze the data. All spectra were referenced at 284.8 eV using the C-C bond of the C 1s orbital.

Energy dispersive X-ray spectroscopy (EDX) and HAADF-STEM: These measurements where performed using a FEI Tecnai Osisris TEM in scanning mode at an acceleration voltage of 200 kV. This microscope is equipped with a high brightness X-FEG gun, silicon drift Super-x EDX detector and Bruker Esprit acquisition software. The sample was prepared by drop casting from hexane or octane on copper or gold grids.

Synthesis

PbS:

PbS NCs were synthesized through a previously published method.[1,2] In brief, 0.669 g of PbO was added to a three-neck flask with 25 mL ODE, 5.7 mL OA and 0.395 mL of OLA. This reaction was heated to 120°C for 1 hour under a nitrogen atmosphere. The solution was then cooled to 90°C. A solution containing 0.09 mL of TMS-S in 1 mL toluene was then swiftly injected. The solution quickly turned black. The reaction was left for 90 seconds. Then a water bath was used to cool the reaction to room temperature. Purification was performed by adding twice the volume of acetone and centrifuging at 6000 rpm (4140 rcf) for 1 minutes. The solution was then redispersed in 5mL hexanes. Four more antisolvent purification with acetone were performed. The sample was then dissolved in the desired solvent and stored in a nitrogen filled glovebox.

$CsPbBr_3$:

Unconfined $CsPbBr_3$:

First a stock solution of cesium oleate was prepared by adding 0.22g of cesium carbonate into 8.25 mL of ODE and 0.67 mL OA.[3] This solution was degassed and then heated to 150°C for 1hour. The solution was then cooled to 100°C. In a three-neck flask 0.28g of $PbBr_2$ was added with 20 mL ODE, 2 mL OA and 2mL OLA. This solution was degassed at 120°C and then heated to 160°C in a nitrogen atmosphere. Then 1.6 mL of the cesium oleate solution was swiftly injected into the three-neck flask. The mixture rapidly turned bright green. The flask was cooled to room temperature with a water bath. The solution was then centrifuged at 6000 rpm (4140 rcf) for 2 hours. The precipitate was discarded and the supernatant was purified by the addition of 4 times the volume of ethyl acetate. This solution was centrifuged at 6000 rpm for 10 minutes. The precipitate was then collected and dissolved in the desired solvent in a nitrogen filled glovebox.

Confined $CsPbBr_3$:

For this reaction the Cs-oleate was prepared by heating to 150°C a solution containing 0.25g cesium carbonate 0.9 mL OA and 8.9 mL ODE under a nitrogen atmosphere for 1 hour.[4] The solution was then cooled to 100°C. In a three-neck flask, 75mg of $PbBr_2$ and 300mg of $ZnBr_2$ were added with 5mL ODE, 2 mL OLA and 2mL OA. This solution was then heated to 120°C for 1 hour. Then 0.4mL of the Cs-oleate was rapidly injected. The reaction was left for 120s and then cooled with a water bath. The solution was then collected and centrifuged for 2 hours at 6000 rpm (4140 rcf). The precipitate was discarded and the supernatant was purified by the addition of acetone. The precipitate was then collected and dissolved in the desired solvent in a nitrogen filled glovebox.

$NaYF_4$:

In three neck flaks, 105 mg of $Y(CH_3CO_2)_3 \cdot H_2O$ was mixed with 4mL Olac and 6 mL ODE.[5] This solution was heated to heated to 150°C for 1 hour and then cooled to room temperature. A solution of 60mg NH_4F and 40mg NaOH in 6mL methanol was then added to the three-neck flask. The flask was heated to 50°C under ambient conditions for 1 hour. The flask was then sealed and degassed at 120°C for 30 minutes. Then the reaction was rapidly heated to 290°C and kept at this temperature for 2 hours. The reaction was then cooled with an air gun. The solution was then purified with twice the volume of ethanol and centrifuged at 6000 rpm (4140

rcf) for 10 minutes. This process was repeated four times. The precipitate was then collected and dissolved in the desired solvent in a nitrogen filled glovebox.

$NaGdF_4$:Yb:

In a three neck flask, 66mg of $Gd(CH_3CO_2)_3 \cdot H_2O$ and 70mg $Yb(CH_3CO_2)_3 \cdot H_2O$ was mixed with 5 mL of OLAC and 5 mL of ODE.[5] This solution was heated to heated to 150°C for 1 hour and then cooled to room temperature. A solution of 50mg NH_4F and 40mg NaOH in 6mL methanol was then added to the three-neck flask. The flask was heated to 50°C under ambient conditions for 1 hour. The flask was then sealed and degassed at 100°C for 30 minutes. Then the reaction was rapidly heated to 270°C and kept at this temperature for 1 hours. The reaction was then cooled with an air gun. The solution was then purified with twice the volume of ethanol and centrifuged at 6000 rpm (4140 rcf) for 10 minutes. This process was repeated four times. The precipitate was then collected and dissolved in the desired solvent in a nitrogen filled glovebox.

$Cu_{2-x}S$:

In a three-neck flask we added 105 mg $Cu(acac)_2$, 16 mg elemental sulfur, 2mL DDT and 4mL OLAC.[6] The solution was degassed at 80°C for 1 hour. Then under a nitrogen atmosphere, the reaction was heated to 220°C for 20 minutes. The solution was then cooled down to room temperature and purified with acetone and centrifuged at 6000 rpm (4140 rcf) for 2 minutes. This process was repeated four times. The precipitate was then collected and dissolved in the desired solvent in a nitrogen filled glovebox.

$CuInS_2$:

In a three neck flask we added 24.4 mg $Cu(acetate)_2$, 58.2mg of $In(CH_3CO_2)_3$, 0.5mL DDT and 5mL ODE.[7] The solution was degassed at 80°C for 1 hour. Then under a nitrogen atmosphere, the reaction was heated to 240°C for 10 minutes. The reaction was then cooled with an air gun. The solution was then purified with twice the volume of acetone and centrifuged at 6000 rpm (4140 rcf) for 2 minutes. This process was repeated four times. After the second purification step 0.1 mL of Olam was added to help in the redispersion of the NCs. The precipitate was then collected and dissolved in the desired solvent in a nitrogen filled glovebox.

Characterization

Summary:

All nanocrystals where synthesized following established protocols. PbS NCs where prepared through the hot injection of hexamethyldisilathiane (TMS-S) into a heated solution of lead oleate in 1-octadecene (ODE).[1,2] After five antisolvent precipitation this process yielded 2.9 ± 0.5 nm particles as measured by transmission electron microscopy (TEM, Figure S1) and corroborated by established sizing curves relating the excitonic peak position measured by optical absorption measurements to NC size (Figure S1).[8] ^{1}H NMR revealed through the broad alkene resonance around 5.7 ppm that the surface is passivated by oleate ligands and that free ligand contributions are marginal (Figure S1).

$CsPbBr_3$ NCs were synthesized by the injection of Cs-oleate into a heated solution containing $PbBr_2$, oleic acid (OLAC), oleylamine (OLAM) and ODE.[3] These NCs where purified only once by antisolvent reprecipitation with ethyl-acetate as further purification steps resulted in the degradation of the NC ensemble. The obtained NCs had an average edge length of 11 ± 2 nm as per TEM (Figure S2). ^{1}H NMR revealed the highly dynamic nature of the surface ligands of these NCs through the presence of narrow alkene resonances (Figure S2). This has previously been associated to a highly dynamic passivation by oleylamonium-bromide and oleylamonium-oleate.[9]

Quantum confined $CsPbBr_3$ where synthesized through a similar methodology but adding $ZnBr_2$ as an additive to control the size.[4] This yielded NCs with an average edge length of 5.1 ± 0.6 nm and a sharp excitonic peak at 473 nm as observed in optical absorption (Figure S3). ^{1}H NMR also revealed a dynamic surface passivated by oleylamonium species. In fact, the ammonium peak was clearly visible by ^{1}H NMR around 8 ppm (Figure S3).

$NaYF_4$ where prepared through a heat up reaction involving yttrium acetate, NH_4F, NaOH and OLAC in ODE.[5] This yielded NCs with an average diameter of 23 ± 2 nm as per TEM (Figure S4). The alkene resonance of oleate could not be resolved by ^{1}H NMR but given that OLAC is the sole ligand employed we expect the absence of the resonance to be a consequence of considerable linewidth broadening due to the large NC size (Figure S4).[10]

$NaGdF_4$:Yb where prepared by a heat up reaction involving gadolinium acetate, ytterbium acetate, NH_4F, NaOH and OLAC in ODE. This yielded 13 ± 1. nm particles (Figure S5).[5] The surface chemistry could not be determined by ^{1}H NMR due to the paramagnetic nature of the Gd^{3+} ions. However, we expect a similar surface to the OLAC passivated $NaYF_4$ NCs.

$Cu_{2-x}S$ NCs where prepared by a heat up reaction involving copper acetylacetonate, elemental sulfur, dodecanethiol (DTT) and OLAC.[6] This yielded 5.0 ± 0.7 nm NCs as per TEM (Figure S6) and ^{1}H NMR revealed that the NCs were solely passivated by DDT (Figure S6).

$CuInS_2$ NCs where synthesized though a heat up reaction involving Cu(I)-acetate and indium acetate in a mixture of DDT and ODE.[7] A small amount of OLAM was added during the purification step to help redisperse the sample. This yielded a sample with an average diameter of 2.7 ± 0.5 nm and a surface passivated by DDT and OLAM (Figure S7). Fourier transform infrared spectroscopy (FT-IR) also demonstrated the expected absorption bands for the respective ligands passivating the six nanocrystalline system studied (Figure S1-7).

General data:

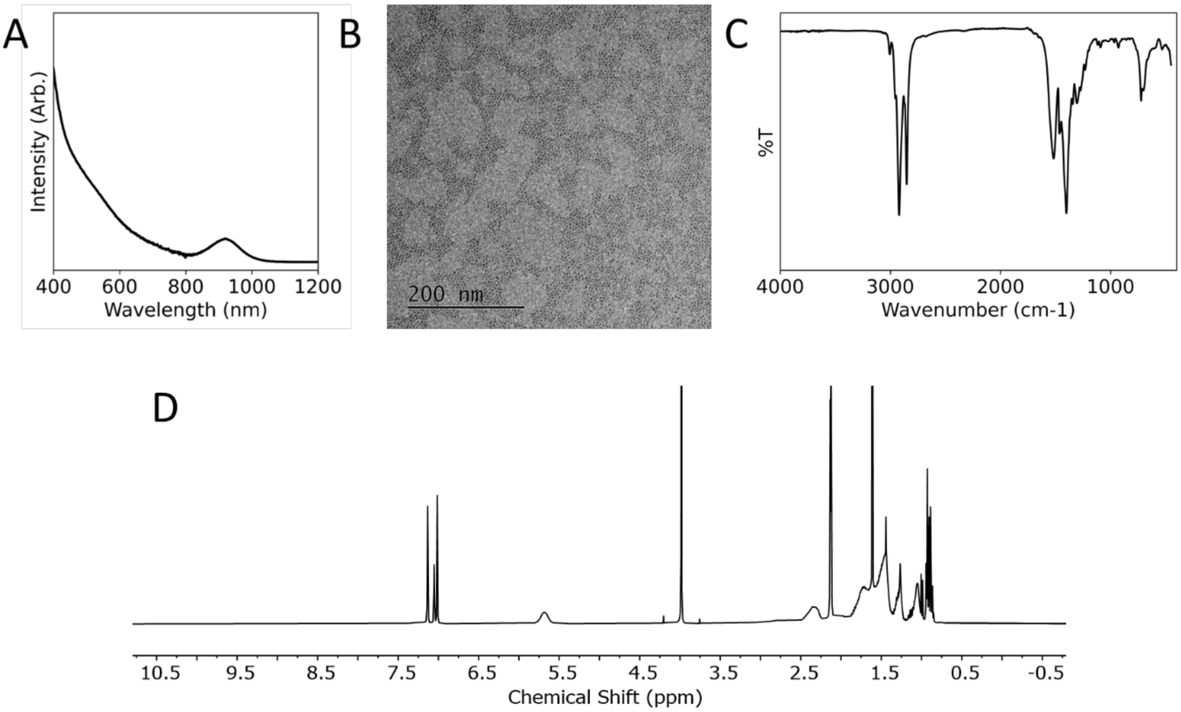

Figure S1: Characterization of as made PbS NCs. A) Optical absorption spectrum with a first excitonic peak at 920 nm. B) TEM of the PbS NCs with an average size of 2.9 ± 0.5 nm. C) FTIR of PbS NCs showing the expected bands for oleate passivation. D) Complete ^{1}H NMR showing oleate passivation.

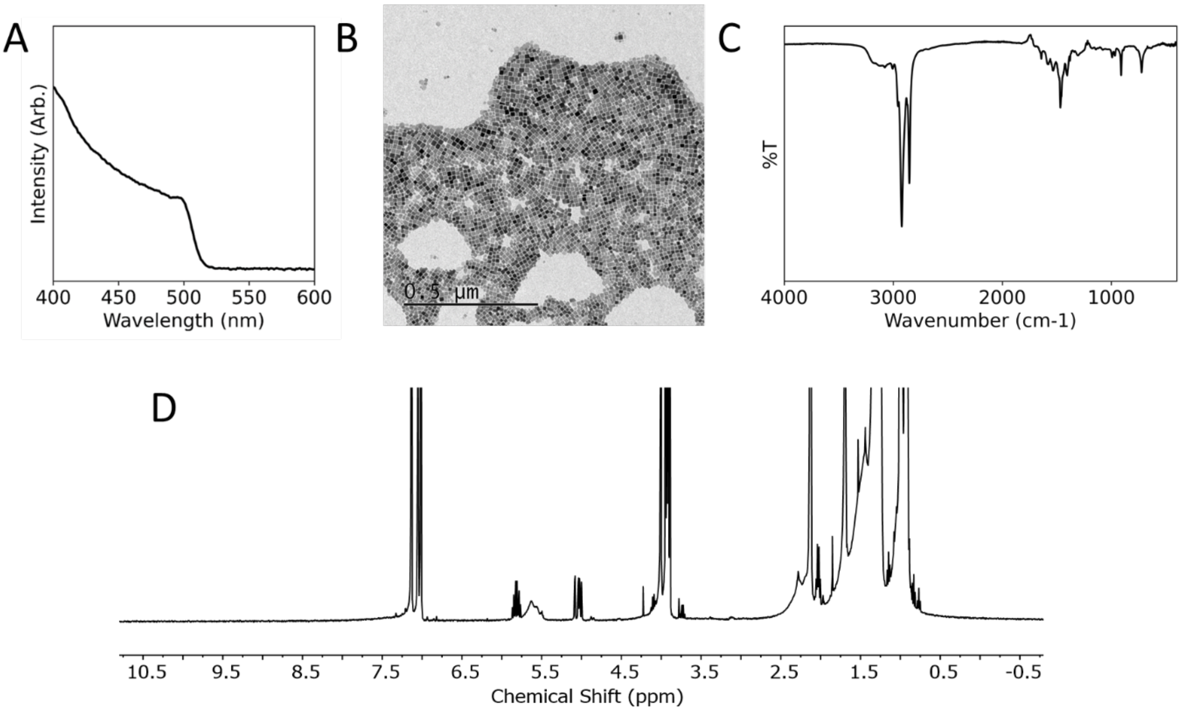

Figure S2: Characterization of as made large $CsPbBr_3$ NCs. A) The optical absorption of the NCs does not display an excitonic peak as is consistent with NCs larger than the Bohr radius of $CsPbBr_3$ (~7 nm). B) TEM of the $CsPbBr_3$ NCs with an average edge length of 11.3 ± 1.6 nm. C) FTIR of $CsPbBr_3$ NCs showing the expected bands for oleate and oleylamonium passivation. D) Complete ^{1}H NMR. Since these sample could not be adequately purified due to the instability of the NCs to polar solvents, residual ODE signal can be observed at 5 and 5.7 ppm. The alkene resonance arises from a mixture of proton from oleic acid and oleylamine, both in a dynamic interaction with the NC surface.

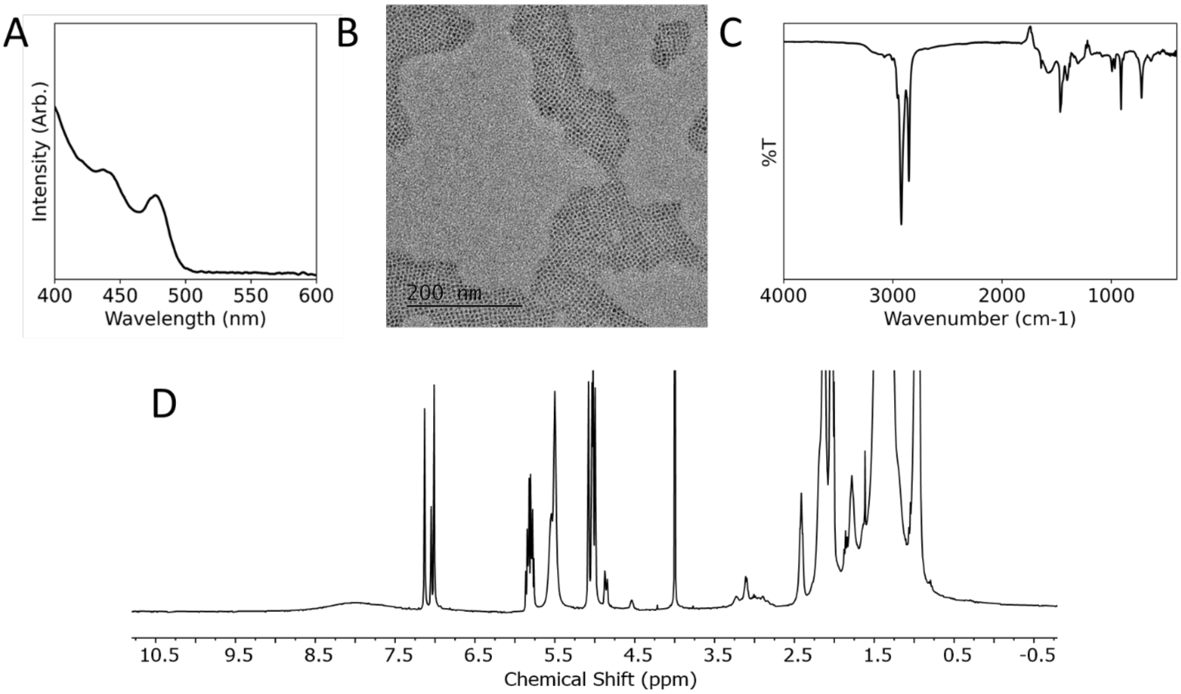

Figure S3: Characterization of as made quantum confined $CsPbBr_3$ NCs. A) The optical absorption of the NCs displays an excitonic peak at 471 nm. B) TEM of the $CsPbBr_3$ NCs with an average edge length of 5.1 ± 0.6 nm. C) FTIR of $CsPbBr_3$ NCs showing the expected bands for oleate and oleylammonium passivation. D) Complete ^{1}H NMR. Since these sample could not be adequately purified due to the instability of the NCs to polar solvents residual ODE signal can be observed at 5 and 5.7 ppm. The alkene resonance arises from a mixture of proton from oleic acid and oleylamine, both in a dynamic interaction with the NC surface. The broad signal around 8 ppm is associated to ammonium protons from oleylammonium.

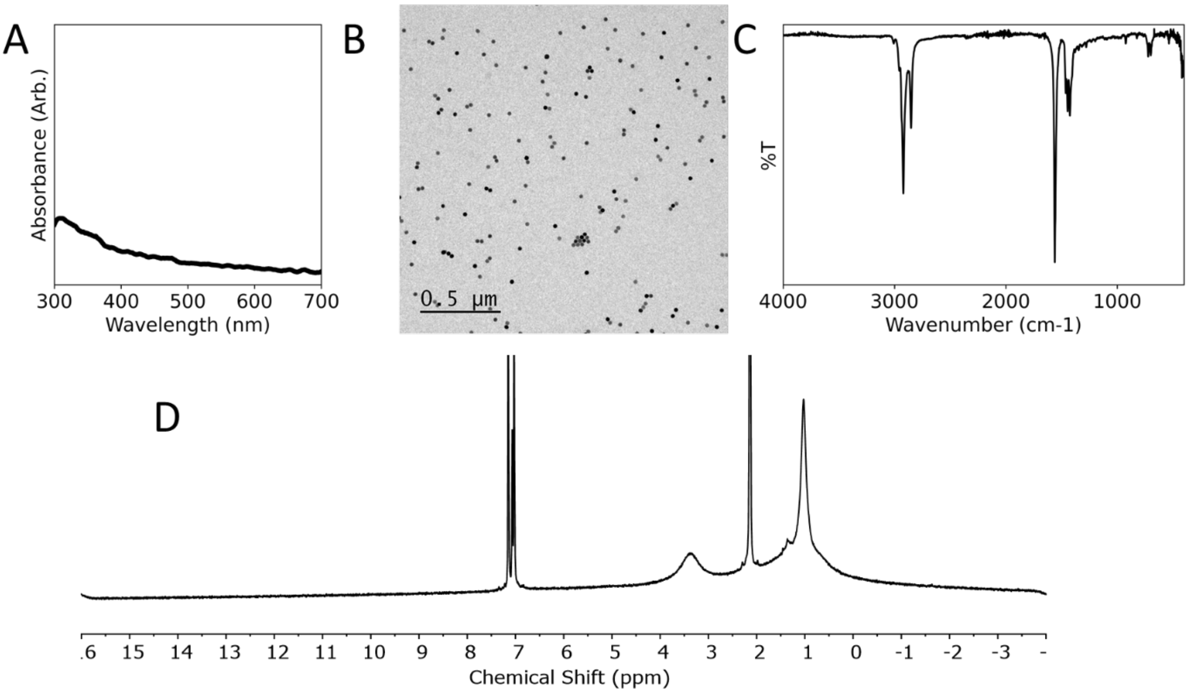

Figure S4: Characterization of as made $NaYF_4$ NCs. A) The optical absorption of the NCs is featureless. Barely any absorption was observed between 300 nm and 700 nm. The rise towards higher energies could be due to scattering. B) TEM of the $NaYF_4$ NCs with an average diameter of 23.1 ± 1.9 nm. C) FTIR of $NaYF_4$ NCs showing the expected bands for oleate passivation. D) Complete ^{1}H NMR showing oleate passivation. The alkene resonance is too broad to resolve, this most likely arises from the large linewidth broadening induced by the large size of the NCs.

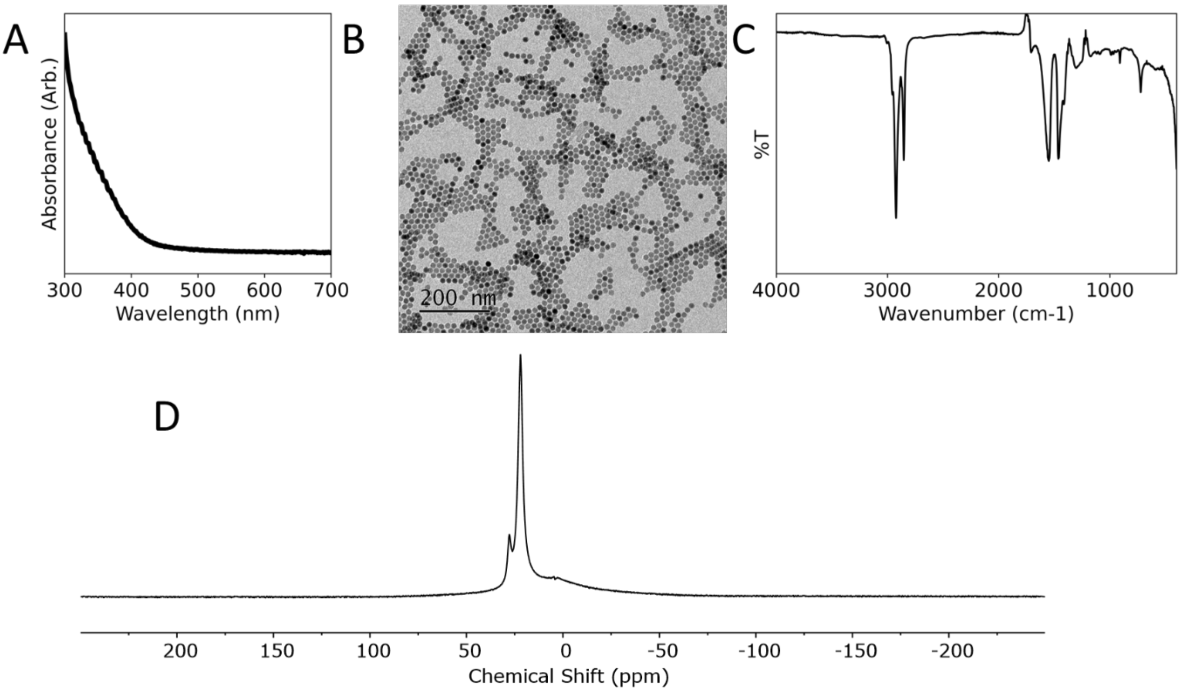

Figure S5: Characterization of as made $NaGdF_4$:Yb NCs. A) The optical absorption of the NCs is featureless with a rise around 400 nm. B) TEM of the $NaGdF_4$:Yb NCs with an average diameter of 13.2 ± 1.0 nm. C) FTIR of $NaGdF_4$:Yb NCs showing the expected bands for oleate passivation. D) Complete ^1H NMR. This technique was incompatible with these NCs because of the significant linewidth broadening of the proton resonance due to the paramagnetic nature of the gadolinium ions. Demonstrated here is a spectrum acquired by initially locking on a blank sample containing only deuterated toluene and then exchanging the sample. The linewidths observed are too broad to extract any valuable information regarding the binding state of the ligands or their nature.

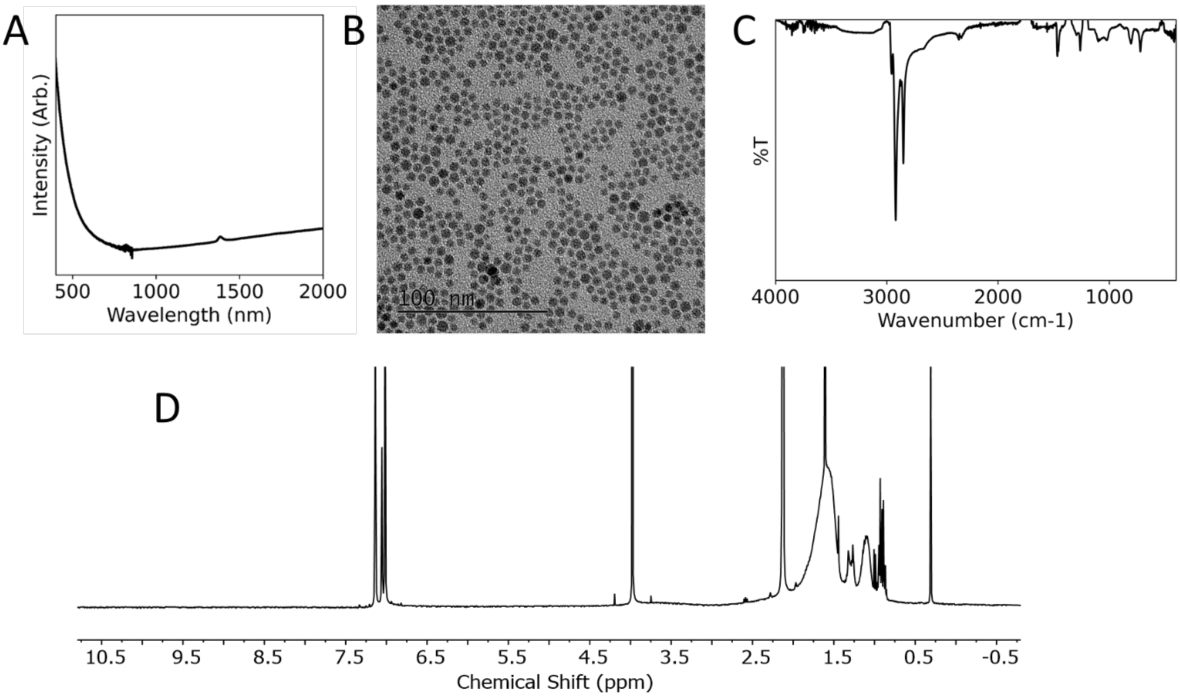

Figure S6: Characterization of as made $Cu_{2-x}S$ NCs. A) The optical absorption of the $Cu_{2-x}S$ NCs displays a constant increase in absorption between 1000 and 2000 nm as consistent with the plasmonic nature of these NCs. B) TEM of the $Cu_{2-x}S$ NCs with an average diameter of 5.0 ± 0.7 nm. C) FTIR of $Cu_{2-x}S$ NCs showing the expected bands for DDT passivation. D) Complete ^{1}H NMR showing DDT passivation.

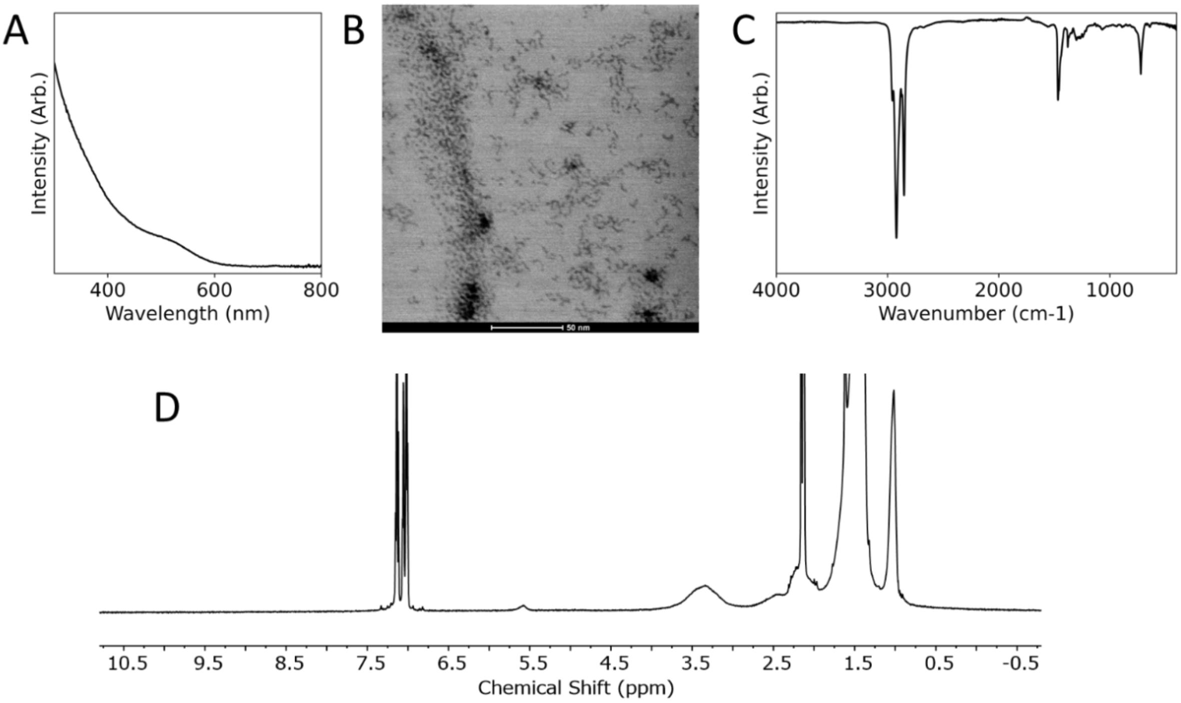

Figure S7: Characterization of as made $CuInS_2$ NCs. A) The optical absorption of the $CuInS_2$ NCs displays a broad excitonic feature around 550 nm. B) TEM of the $CuInS_2$NCs with an average diameter of 2.7 ± 0.5 nm. The NCs formed large aggregates when dispersed on the TEM grid, rendering an accurate estimation of the average NC size difficult. C) FTIR of $CuInS_2$ NCs showing the expected bands for DDT passivation. D) Complete ^{1}H NMR showing DDT passivation and a small fraction of OLAM.

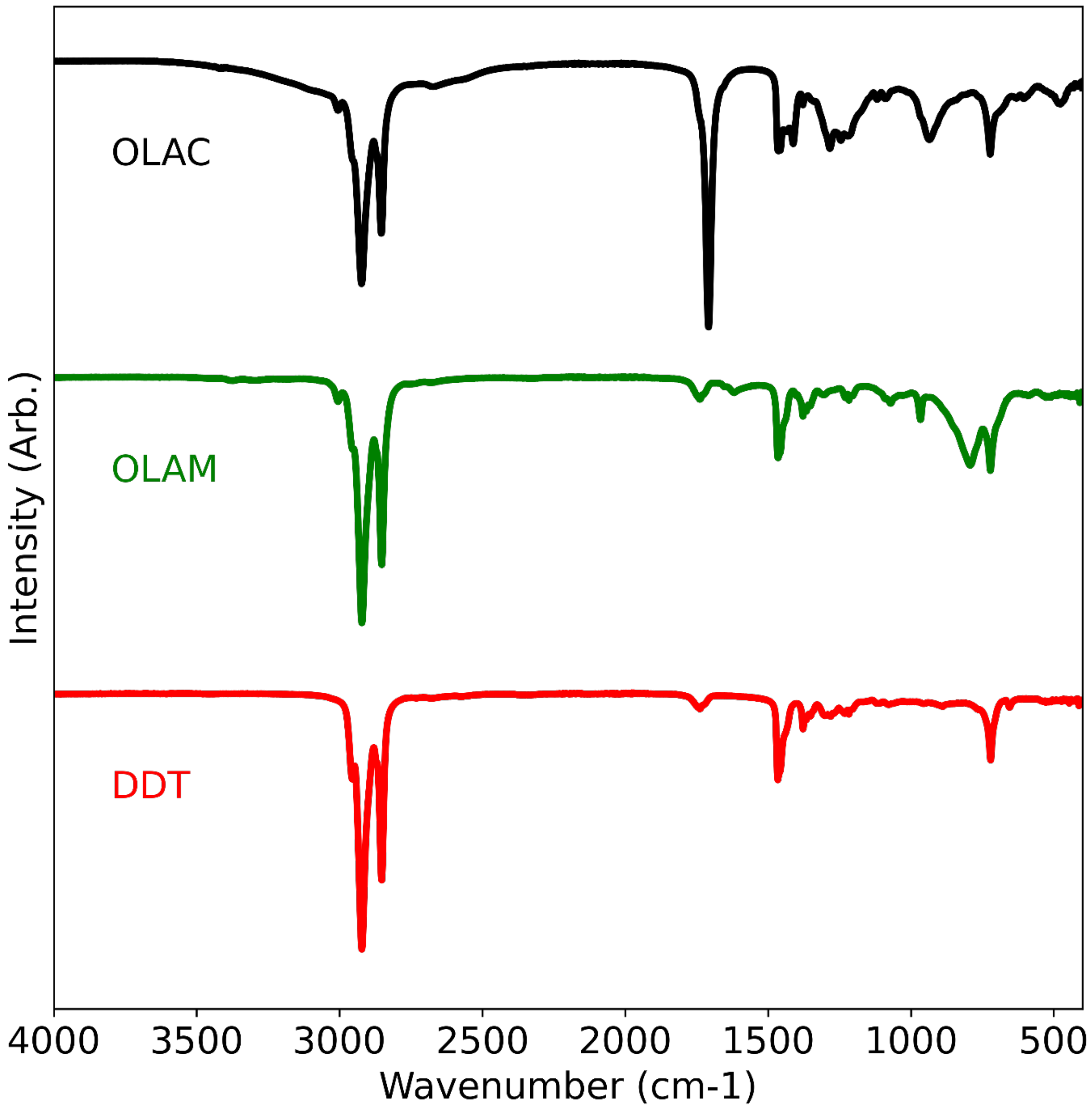

Figure S8: FTIR of the ligands employed during colloidal synthesis to consider as refences for the data reported for each NC above. Black: oleic acid (OLAC) employed in the synthesis of PbS, $CsPbBr_3$, $NaYF_4$ and $NaGdF_4$:Yb; Green: oleylamine (OLAM) employed in the synthesis of $CsPbBr_3$ and $CuInS_2$; Red: dodecanethiol (DDT) employed in the synthesis of $Cu_{2-x}S$ and $CuInS_2$. Difference in the observed resonances for free OLAC and passivating OLAC have been observed and reveal the binding state of the ligand (Figure S1-S7).[11,12] The state is revealed by the difference in the asymmetric and symmetric stretches between 1400 and 1700 cm^{-1}. For free OLAC the difference is above 250 cm^{-1}, consistent with a monodentate coordination. When bound such as on the PbS, $NaYF_4$ and $NaGdF_4$:Yb NCs the difference is considerably narrower consistent with OLAC being in a chelating configuration.

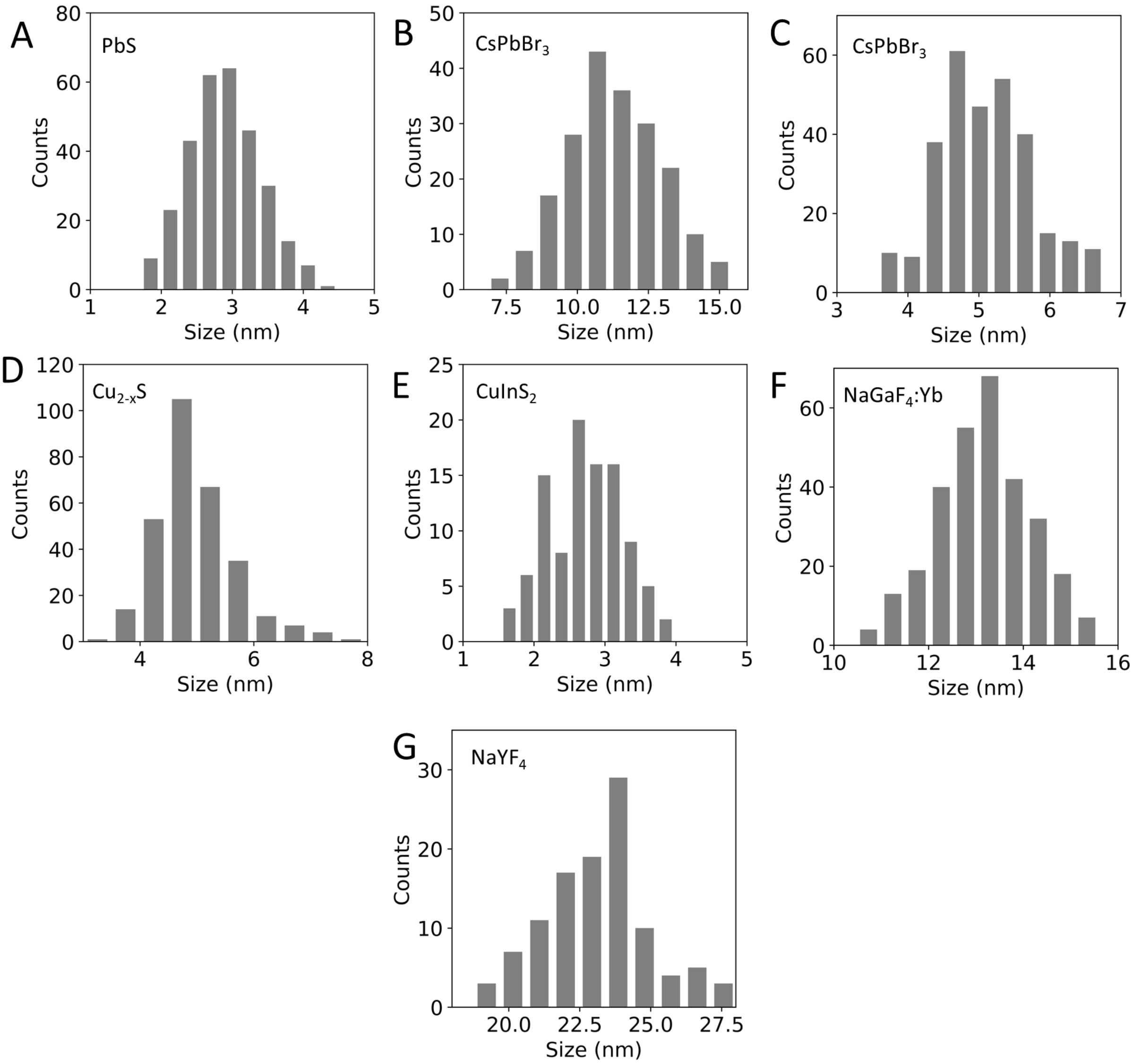

Figure S9: Summary of the NC sizes. Sizing histograms for A: PbS, B: $CsPbBr_3$, C: quantum confined $CsPbBr_3$, D: $Cu_{2-x}S$, E: $CuInS_2$, F: $NaGdF_4$:Yb and G: $NaYF_4$. A summary of all the size can be found in table S1.

Table S1: Average NC sizes for all studied nanocrystalline composition as extracted from TEM

NC composition	Size (nm)	Standard deviation (nm)
**PbS**	2.9	0.5
**$CsPbBr_3$**	11.3	1.6
**$CsPbBr_3$**	5.1	0.6
**$Cu_{2-x}S$**	5.0	0.7
**$CuInS_2$**	2.7	0.5
**$NaGdF_4$:Yb**	13.2	1.0
**$NaYF_4$**	23.1	1.9

Colloidal Atomic Layer Deposition

Procedure:

A stock TMA solution in anhydrous octane was prepared in a nitrogen filled glovebox. All equipment used to prepare this solution was then quenched through dilution with hexane (500x the volume) followed by the addition of isopropanol. This step releases methane in the glovebox. Extreme care was taken while manipulating concentrated TMA solutions as it is highly pyrophoric.

The TMA solution employed for c-ALD was prepared by mixing the stock TMA solution with anhydrous octane to yield a 20mM solution. This solution was then transferred to a gas tight syringe (S.G.E. Gas Tight Luer Lock Syringe 5mL) with a stainless steel 304 syringe from Sigma Aldrich. The syringe was purged with nitrogen before introducing TMA.

From a nitrogen filled glovebox a 3mL solution of ~34 uM NC in anhydrous octane was prepared from the stock NC solution. This solution was then transferred to a three-neck flask that had been previously purged with nitrogen. The nitrogen was supplied through a three-way valve to ensure the constant renewal of nitrogen and replacement of the introduced O_2 and generated methane. The solution was constantly stirred at room temperature for the duration of the experiment.

The general procedure for the growth of the alumina shell was performed as follows but can be modified according to the desired application and NC core (see comments for each nanocrystalline composition below). Each TMA step involved the injection of 50uL of the TMA solution at a rate of 1mL/hr using a syringe pump (Chemyx Fusion 200 two-channel injection pump). The TMA injection was then followed either by the bubbling of O_2 or the introduction of ligands. The bubbling of O_2 was performed using a mass flow controller at a rate of 1.5 mL/min for 2 min.

A representative synthesis of PAH/NC@AlOx can be summarized as follows. First, an initial ligand exchange can be performed to introduce some PAH ligands on the surface. This step is particularly relevant when energy transfer is required, but cannot be performed when the added ligand don't bind the surface, such as PAH on $CuInS_2$ or $Cu_{2-x}S$ NCs. Then, we initiated the c-ALD process by growing a thin alumina shell through three cycles of TMA and O_2 (3x [TMA/O_2] cycles). At this point, if thicker shell and higher loading of PAH were targeted, we added a ligand mixture containing the PAH and OLAC (L: PAH + OLAC). OLAC was added because the NC solution loses colloidal stability without its addition. Addition of the ligands can be performed after every two additions of TMA up to the desired shell thickness and loading of PAH. Here we added TMA followed by the ligands and then closed with a complete [TMA/O_2]. This step can be repeated arbitrarily (*n* x[TMA/L/TMA/O_2]). Finally, the process was terminated by three more cycles (3x[TMA/O_2]). The whole process can be summarized as 3x[TMA/O_2] + *n* x[TMA/L/TMA/O_2] + 3x[TMA/O_2] (L: 9-ACA + OLAC).

Each [TMA/L/TMA/O_2] resulted in the addition of 10 to 20 molecules of each PAH per NC. This amount of PAH molecules per NC ratio was selected as it did not compromise colloidal stability.

The ligands such as OLAC, 9-ACA, 9-PTA and 1-PCA were introduced using a purged needle and directly injected into the three-neck flask. After each step, whether TMA, O_2 or ligands was introduced a minimum 5-minute delay between steps was performed.

After the desired number of cycles, the solution was dried under a constant flow of nitrogen and the NCs were redispersed in the desired anhydrous solvent such as deuterated toluene or octane and stored in a nitrogen filled glovebox.

The gas tight syringe was then washed by flowing 15 mL of anhydrous hexanes to avoid alumina formation and clogging of the syringe. The syringe was then placed in a 60°C oven. If alumina deposits are observed 1M HCl solution was placed in the syringe and left for 1hr. The syringe was then thoroughly rinsed and stored in the oven.

Additional Notes:

Note: The outlined protocol is highly versatile, in that it can be performed in various ways to obtain a similar outcome. A general scheme would be as follows: PAH (purification if possible) + m x[TMA/O_2] + n x[TMA/L/TMA/O_2] + p x[TMA/O_2]. An optional PAH addition at the end is also possible, but care must be taken to not add excess ligands that would not be bound.

Note: the addition of excess TMA can result in the precipitation of the NCs. It was thus useful for us to initially titrate the NC solutions with TMA until precipitation was visibly observed. Then we employed half of this amount in each cycle of the growth process.

Note: To verify if the growth occurs we found it helpful to extract aliquots of the solution and to take an FTIR spectrum. If the four bands discussed in the manuscript and highlighted in Figure S9 were present, we expected the successful growth of an alumina shell. These bands can usually be observed after 2 cycles. If the bands were not present, we suspected either the TMA was quenched or growth of a shell was incompatible with the NC surface chemistry.

Note: For the growth a hybrid shell comprising of a PAH ligand that can accept triplet excitons, it was observed to be necessary to wrap the flask in aluminum foil to prevent light exposure. If the system is unlikely to undergo energy transfer this precaution was not observed to be necessary.

Note: 9-ACA, 9-PTA and 1-PCA are poorly soluble in octane or toluene, hence the solution was thoroughly mixed before addition. We estimate that this lack of solubility results in variations in the amount of PAH added. We consider that 10 to 20 PAH molecules per NC is added at every addition of PAH ligand.

Note: All glassware was kept at 110°C for at least two hours before c-ALD due to the high sensitivity of TMA to air and water

c-ALD on carboxylated surfaces (PbS, $NaGdF_4$:Yb and $NaYF_4$ NCs):

c-ALD on such NCs can be performed with or without an initial ligand exchange. On PbS NCs exchanged with 9-ACA, shells of alumina could be grown but to the expense of colloidal stability. This resulted in the precipitation of the NCs after only 4 c-ALD cycles. In contrast, the as-synthesized sample could withstand up to 8 cycles without loss of colloidal stability.

The example we highlighted to generate 9-ACA/PbS@AlO_x NCs in Figure 2 was performed as follows 3x[TMA/O_2] + 6 x[TMA/L/TMA/O_2] + 3x[TMA/O_2] (L: PAH + OLAC). The same procedure was performed on $NaGdF_4$:Yb and $NaYF_4$ NCs. This method allowed for a colloidally stable product with a high loading of 9-ACA. The last 3x[TMA/O_2] cycles were performed to ensure all ligands were locked in the structure.

c-ALD on oleylammonium-(bromide or carboxylate) surfaces ($CsPbBr_3$ NCs):

Similar to carboxylated surfaces, the initial ligand exchange is optional. However, introducing an initial amount of PAH via ligand exchanged prior to the shell grows is beneficial for triplet energy transfer in the end product. This outcome is expected because of the shorter distance of exchanged ligands compared to those incorporated during the alumina growth. The protocol used for these samples was the following: PAH + [TMA/O_2] + [TMA/OLAC] +[TMA/PAH] 3x[TMA/O_2]. Each PAH or OLAC addition resulted in approximately 30 added molecules per NC. This process can be continued to include more PAH ligands.

c-ALD on thiolate surfaces ($CuInS_2$ and $Cu_{2-x}S$ NCs):

For these NCs, no ligand exchange was performed as the added ligands do not interact with the starting surface. Therefore, PAH and OLAC were only introduced after 3x[TMA/O_2]. Then a certain number of [TMA/L/TMA/O_2] cycles can be performed before terminating the process with 3x[TMA/O_2].

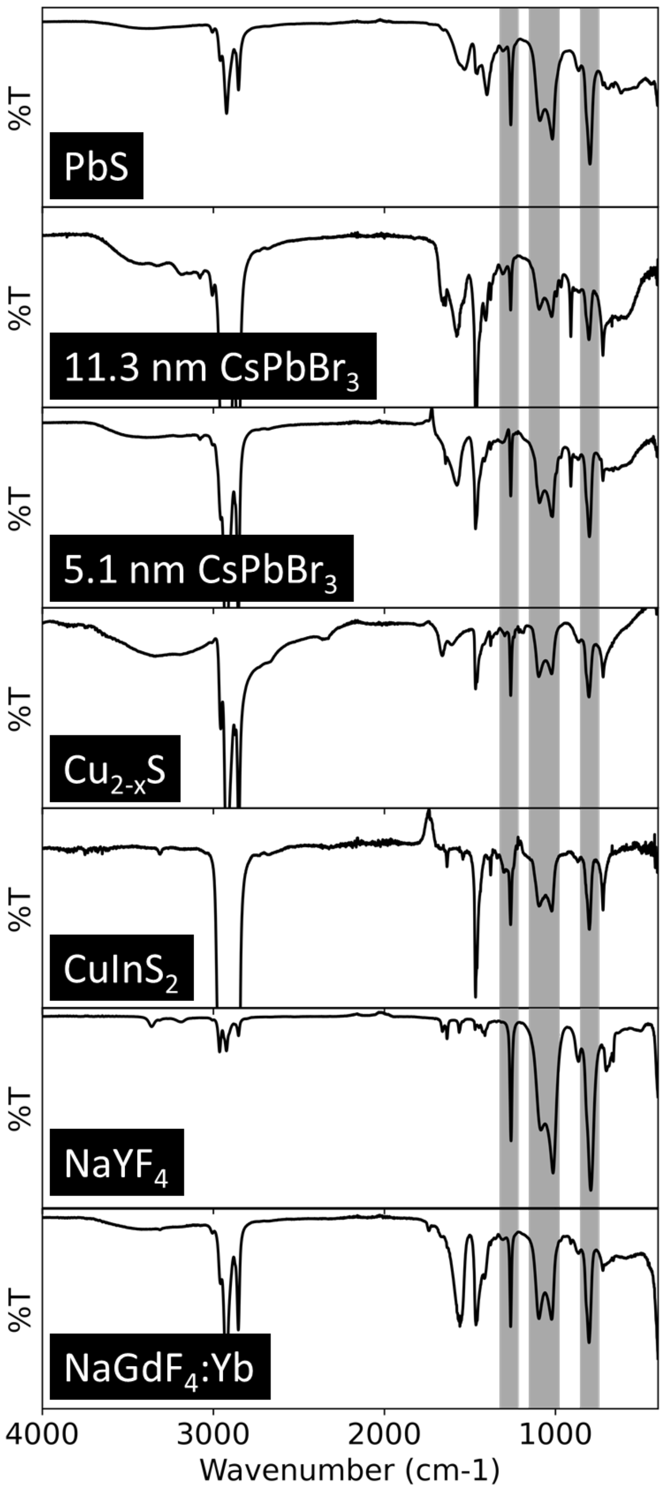

Figure S9: FTIR of all nanocrystalline compositions after c-ALD. Four bands characteristic of amorphous alumina are observed at 1260, 1094, 1023 and 801 cm^{-1}.[13] The presence of these bands is a strong indicator that an alumina shell is growing around the NC core, as they are not observed if alumina is homogeneously nucleated by reacting TMA and O_2 or when TMA is added to free OLAC.[13] Consequently, these data suggest the presence of AlO_x shells and not bulk alumina. As for the ligands, their bands remain the same of the as-synthesized NCs (Figure S1-S7).

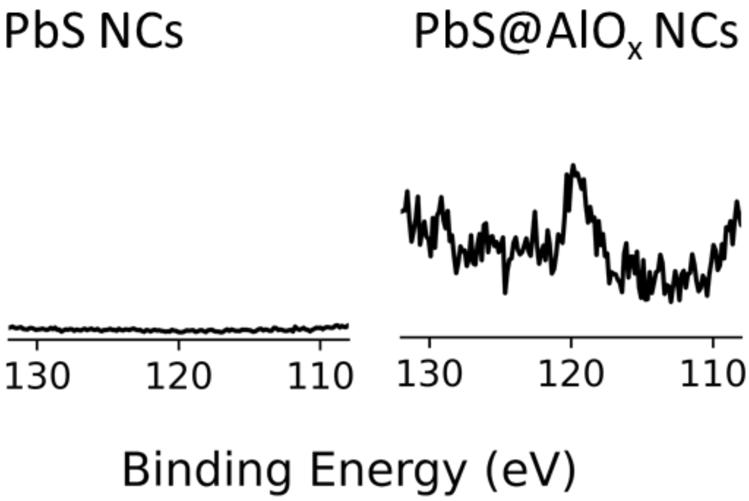

Figure S11: XPS spectra of the Al 2s core regions of PbS NCs and PbS@AlO$_x$ NCs. Signal arising from Al 2s orbitals is only observed after shell growth.

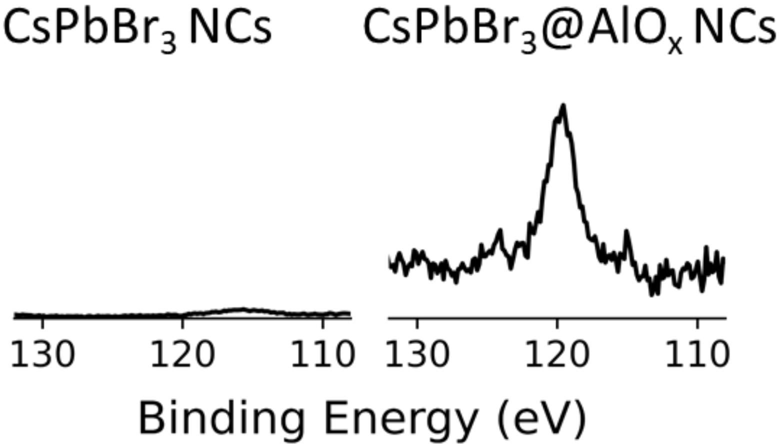

Figure S12: XPS spectra of the Al 2s core regions of 11 nm $CsPbBr_3$ NCs and $CsPbBr_3$ @AlO$_x$ NCs. Signal arising from Al 2s orbitals is only observed after shell growth.

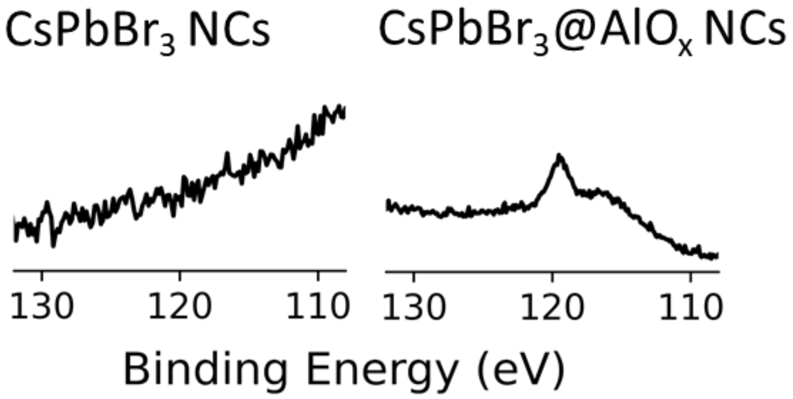

Figure S13: XPS spectra of the Al 2s core regions of 5.1 nm $CsPbBr_3$ NCs and $CsPbBr_3$@AlO_x NCs. Signal arising from Al 2s orbitals is only observed after shell growth.

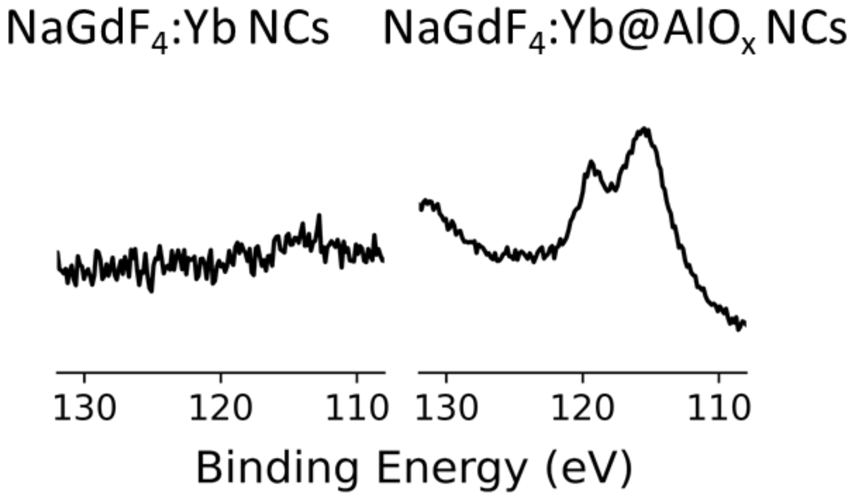

Figure S14: XPS spectra of the Al 2s core regions of $NaGdF_4$:Yb NCs and $NaGdF_4$:Yb@AlO_x NCs. Signal arising from Al 2s orbitals is only observed after shell growth.

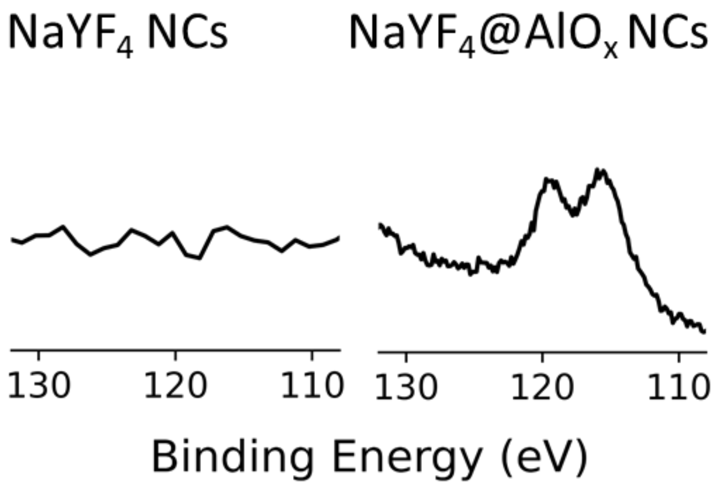

Figure S15: XPS spectra of the Al 2s core regions of $NaYF_4$ NCs and $NaYF_4$@AlO_x NCs. Signal arising from Al 2s orbitals is only observed after shell growth.

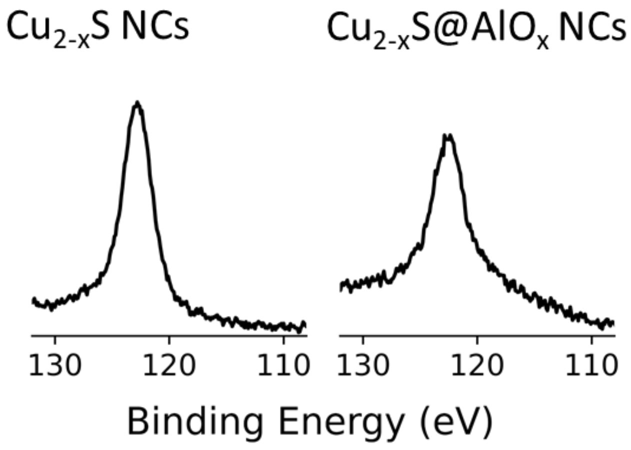

Figure S16: XPS spectra of the Al 2s core regions of $Cu_{2-x}S$ NCs and $Cu_{2-x}S$@AlO_x NCs. The signal from Cu 3p overlaps with the region expected for Al 2s on the native $Cu_{2-x}S$ NCs. However, we note a shoulder around 119 eV on the $Cu_{2-x}S$@AlO_x NCs that is not present on the $Cu_{2-x}S$. This shoulder might be attributed to the presence of alumina.

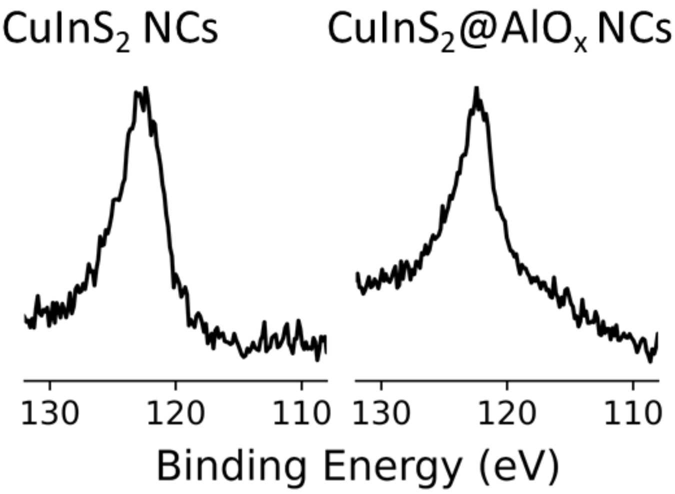

Figure S17: XPS spectra of the Al 2s core regions of $CuInS_2$ NCs and $CuInS_2$@AlO_x NCs. The signal from Cu 3p overlaps with the region expected for Al 2s on the native $CuInS_2$ NCs. However, we note a shoulder around 119 eV on the $CuInS_2$@AlO_x NCs that is not present on the $CuInS_2$. This shoulder might be attributed to the presence of alumina.

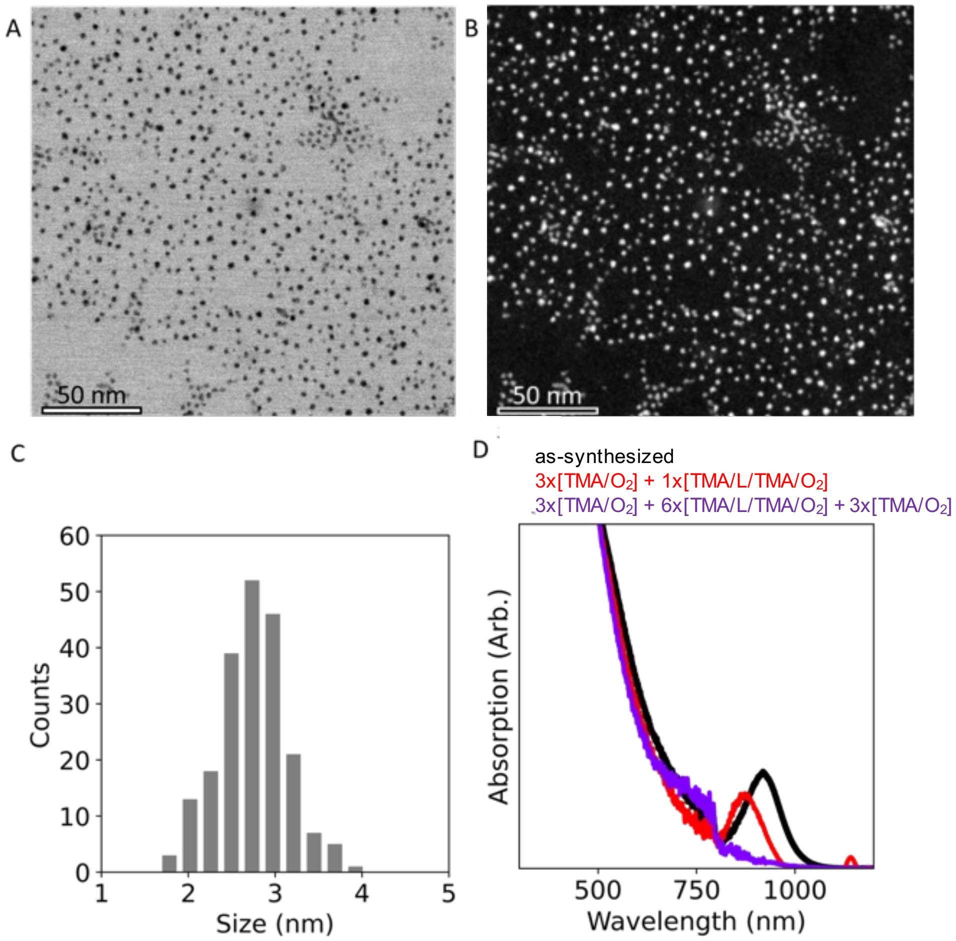

Figure S18: A,B) Representative (A) bright field and (B) dark field STEM images of 9-ACA/PbS@AlOx hybrids. Further data on this sample is presented in Figure 2 of the manuscript. We note that sample beam damage was observed during these measurements, possibly reflecting the high ligand density around the NCs. C) Corresponding size histogram showing an average size of 2.7 ± 0.4 nm. This size is undistinguishable within our resolution from the as-synthesized NCs (2.9 ± 0.5 nm). D) Absorption spectra of the 9-ACA/PbS@AlOx NCs with increasing numbers of c-ALD cycles. A clear blue shift is observed with increasing number of cycles . As there is no substantial size change to justify this shift (absorption at 750 nm would correspond to around 2 nm PbS)[8], this change in absorption can originate from a combination of surface oxidation[14,15], which was observed in a previous study applying the c-ALD to PbS NCs[13], or to an electronic interaction with the ligand shell[16]. Furthermore, we note that the shift is substantial for sample prepared with higher number of cycles (purple trace). However, it decreases for thinner shells prepared with less cycles (red trace). These data indicate that less ALD cycles eventually allow to preserve the electronic properties of the PbS NCs. Indeed, 2x[TMA/O2] cycles resulted in a blue shift of only 0.23 nm.[13] This shift is smaller than the one previously observed for comparably sized PbS NCs which underwent ligand exchanges to introduce PAH ligands.[16]

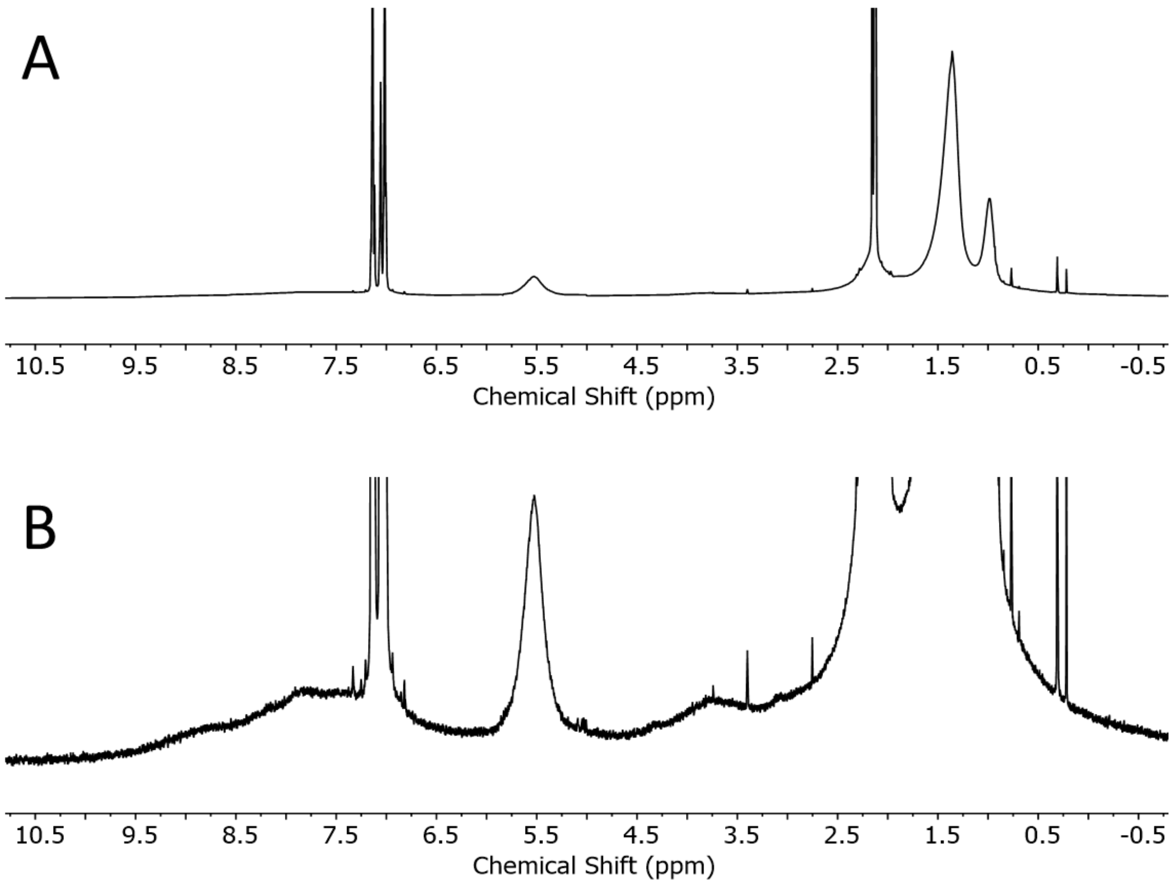

Figure S19: Full ^{1}H NMR of the sample displayed in Figure 2. A) Full spectrum and B) Zoom in to reveal the broad resonances of bound 9-ACA.

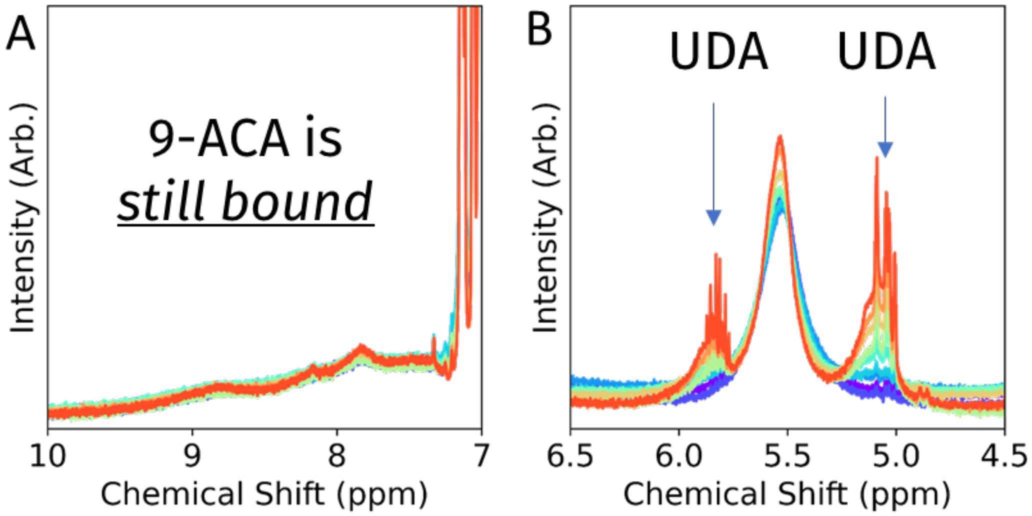

Figure S20: Titration of undec-10-enoic acid (UDA) to 9-ACA/PbS@ to verify if 9-ACA was embedded in the AlO_x shell. UDA was selected as it displays two vinylic resonances centered around 5.1 and 5.8 ppm that are clearly distinguishable from OLAC and 9-ACA. Throughout the titration, we observe a marginal displacement of 9-ACA despite observing a significant portion of freely diffusing UDA (narrow ~2 Hz resonances around 5.8 and 5.05 ppm). A bound contribution of UDA is also observed. Throughout the titration the resonance for OLAC narrows from 88 to 56 Hz and shifts slightly downfield, but remains a single symmetric peak. To comprehend the linewidth narrowing, we note that increasing 9-ACA:OA on the surface of PbS NCs results in larger linewidths. The reintroduction of alkyl chains, such as UDA, increases the alkyl chain density and potentially reinstates the narrower linewidths. In all, we show that c-ALD can be used as a method to bind all added ligands in the newly synthesized 9-ACA/PbS@AlO_x.

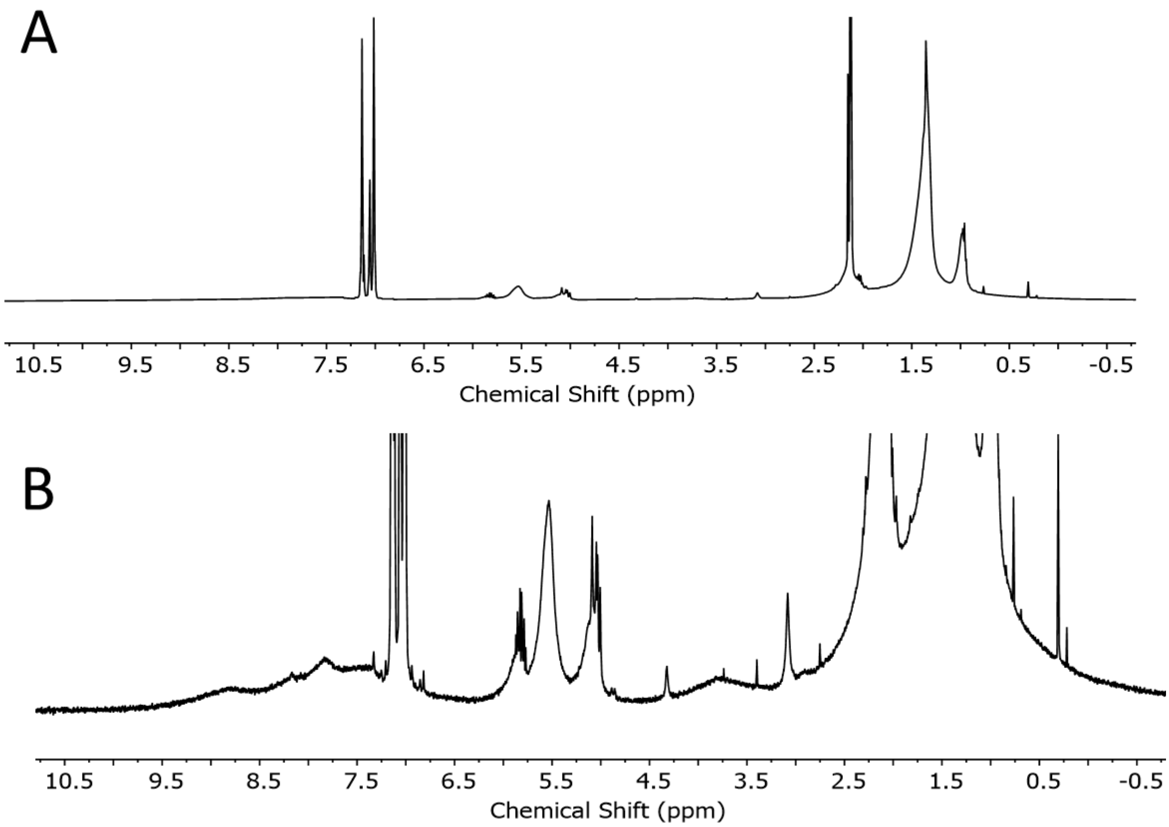

Figure S21: Full ^{1}H NMR of the sample displayed in Figure S20 with added UDA. A) Full spectrum and B) Zoom in to reveal the broad resonances of bound 9-ACA.

Discussion on X-for-X exchange on PbS:

We tested the possibility of introducing photoactive ligands like 9-anthracene carboxylic acid (9-ACA on the surface of PbS NC. To track this process, we titrated the desired ligand in a solution of NCs in deuterated toluene. This initiates the ligand exchange from which the extent can be tracked by ^{1}H NMR by contrasting the broad resonance characteristic of bound ligands versus the narrow resonances associated with free or dynamic ligands.[17] The alkene resonance of the native oleate ligands is conveniently isolated in an ^{1}H NMR spectrum to allow simple quantification of the exchange by monitoring the free versus bound OLAC ratio.[18] This can be seen in figure S22 where we titrate 9-antracene carboxylic acid (9-ACA) to a solution of PbS NCs. As prepared PbS NCs display a resonance around 5.7 ppm with a width of approximately 44Hz. As 9-ACA is titrated (up to 85 equivalents) a new sharper (~18Hz) resonances emerges around 5.5 ppm. Concomitantly, three new resonances associated with 9-ACA are observed at 8.67, 8.11 and 7.73 ppm with linewidths around 20Hz, two more resonances are expected, but are convolved with the deuterated toluene resonance. All four narrow resonances (1 from OLAC and 3 from 9-ACA) are associated with ligands in a dynamic equilibrium with the surface.[17] Of importance is the lack of a clearly resolved signal for bound 9-ACA. This is due to the large linewidth broadening of bound 9-ACA (Figure S22, 23). To yield a sample with solely bound ligands, antisolvent purification is required. Through this procedure the bound 9-ACA resonances can be revealed by ^{1}H NMR, but remains very broad (Figure S23). Nonetheless, this confirms that 9-ACA can be placed on the surface of PbS NCs.

The broad resonance of 9-ACA observed by ^{1}H NMR results in a difficult quantification of 9-ACA. Nonetheless its concentration can be estimated by optical absorption (Figure S23).[17] This is manifested by the optical absorption of 9-ACA a top that of PbS NCs (Figure 22). The optical response for 9-ACA is composed of a primary peak at 390 nm associated with the S0 to S1 transition followed by a Frank-Condon progression with an energy spacing of 1380 cm^{-1} consistent with the C-C bond stretching of 9-ACA. By subtracting the optical absorption of purified 9-ACA passivated NCs and native NCs, the concentration of 9-ACA and consequently the number of 9-ACA units per NC can be estimated. In an effort to increase the amount of bound 9-ACA ligands we added large excess of 9-ACA, from 85 to 380 equivalents. After antisolvent purification to separate free and bound ligands we observed that the 9-ACA:PbS system displayed an equivalent 9-ACA contribution in optical absorption irrespective of the initially added 9-ACA equivalences (Figure S22). This indicates that the number of 9-ACA that can be placed on PbS NCs through an X-for-X exchange is limited. We estimate that this process reaches a maximum of 30 9-ACA per PbS NCs. Quantification of the remaining OLAC yields 90 OLAC per NCs resulting in a total ligand coverage of 120 ligands per NCs as compared to the 132 OA:NCs from native PbS NCs. The slight decrease in ligand coverage is expected due to the requirement of additional purification steps. This result in a 25% coverage by 9-ACA on the surface. This observation is in stark contrast to long tailed ligands such as UDA which can be exchanged to cover more then 90% of the surface, but is similar to exchanges with branched aliphatic carboxylates.[17,19,20] We also noted the significant broadening of the OLAC resonance in mixed 9-ACA-OLAC ligand shells from 44 Hz to 153Hz, an observation that was previously seen to scale with the number of 9-ACA per NC (Figure S22, 23).[17] We also observed a 40meV red shift of the 9-ACA absorption when fully bound (Figure S23). This energy shift can be associated with 9-ACA/NC or 9-ACA/9-ACA coupling.[17,21,22] In sum, this experiment demonstrates that the extent of 9-ACA coverage on PbS NCs is inherently thresholded.

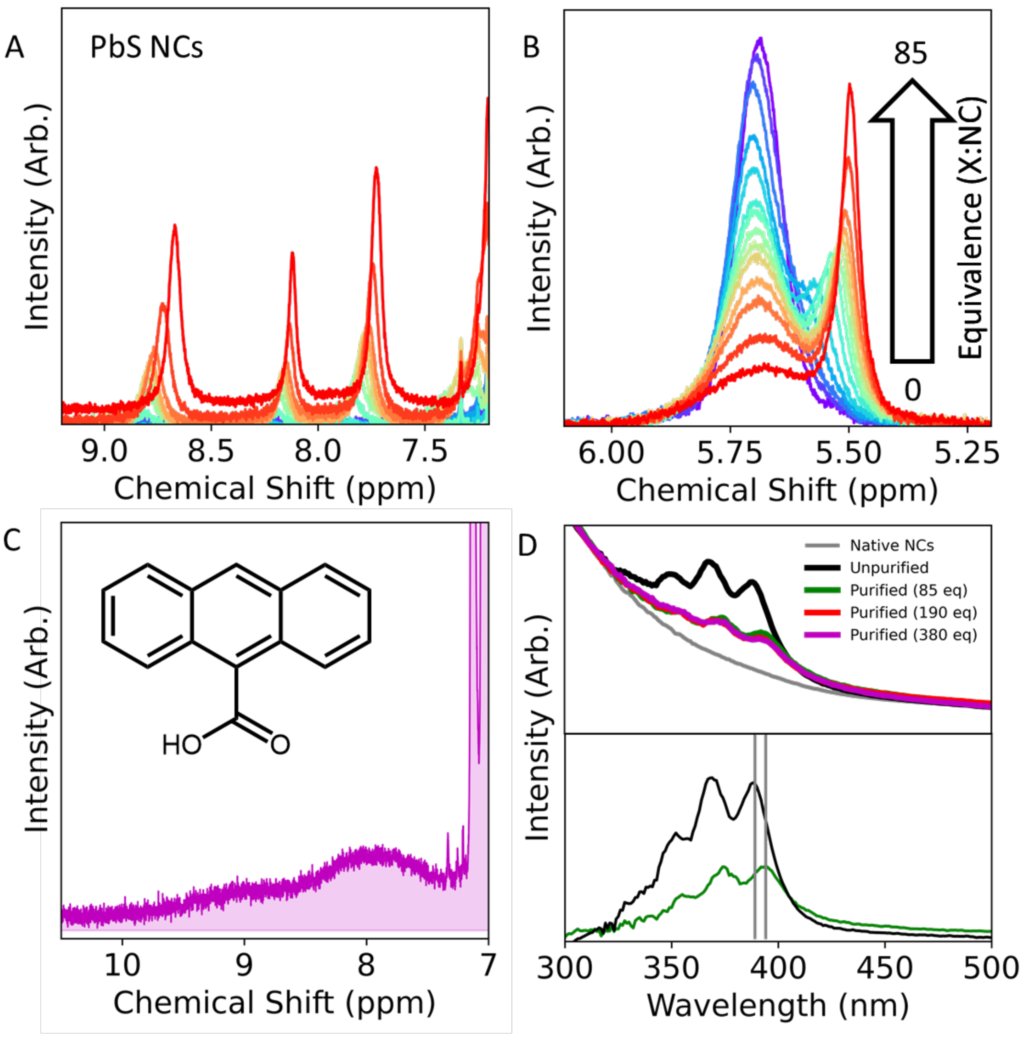

Figure S22: Limitations in the X-for-X exchange of 9-ACA on oleate passivated PbS NCs.

(A, B) ^{1}H NMR of (A) the aromatic region of 9-ACA and (B) the alkene resonances of OLAC. during a titration with 9-ACA. The three resonances (~20 Hz) associated with 9-ACA are observed between 9 and 7.5 pm and they are indicative of dynamically binding 9-ACA. Concurrently, the broad resonances at 5.7 ppm of the alkene protons (~40 Hz) diminishes and broaden at the expense of a narrower resonances (~20 Hz) at 5.5 ppm associated with dynamic OLAC. This reflects an exchange between 9-ACA and OLAC on the surface of the NCS.

C) Purifying samples titrated with 9-ACA renders a NC solution with solely bound ligands. This allows for the observation of the bound 9-ACA resonances as a broad feature between 10 and 7 ppm.

To quantify the bound 9-ACA, (D) optical absorption is used. By subtracting the native NC absorption to the exchanged one the amount of 9-ACA per NC can be quantified. The amount of 9-ACA per NCs is capped to approximately 30, irrespective of the equivalences of 9-ACA employed during the exchange. Subtracting the optical absorption from the native sample from those with exchange 9-ACA, we observed a red-shift in the 9-ACA absorption. This shift can reflect 9-ACA/NC or 9-ACA/9-ACA coupling.[17,21,22] This is only observed when ligands are fully bound.

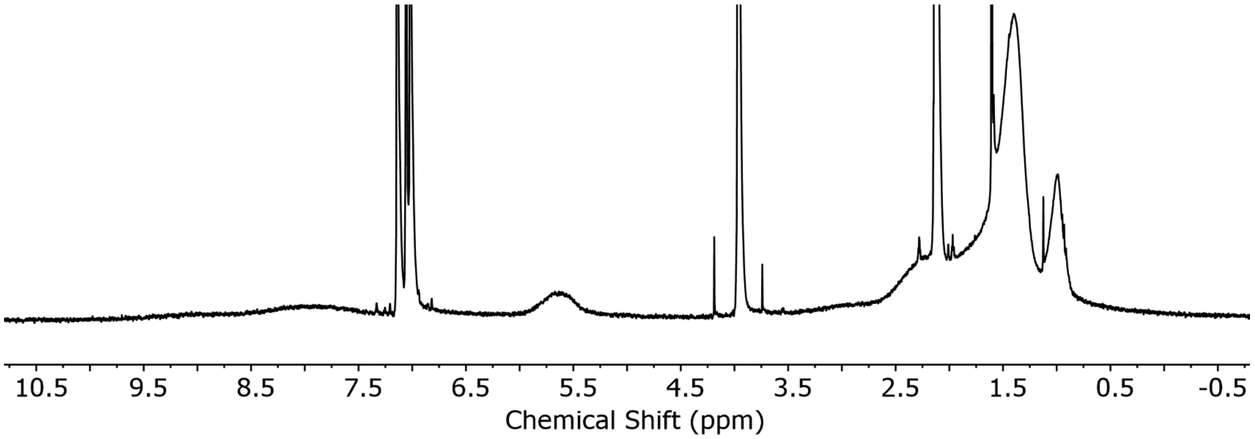

Figure S23: Complete ^{1}H NMR of PbS NCs having undergone a ligand exchange with 9-ACA (380 eq) and then purified three times with acetone. The alkene resonance around 5.6 ppm is seen to broaden from 44 Hz to 153 Hz. A very broad convoluted resonance associated with bound 9-ACA is seen between 10 and 7.5 ppm.

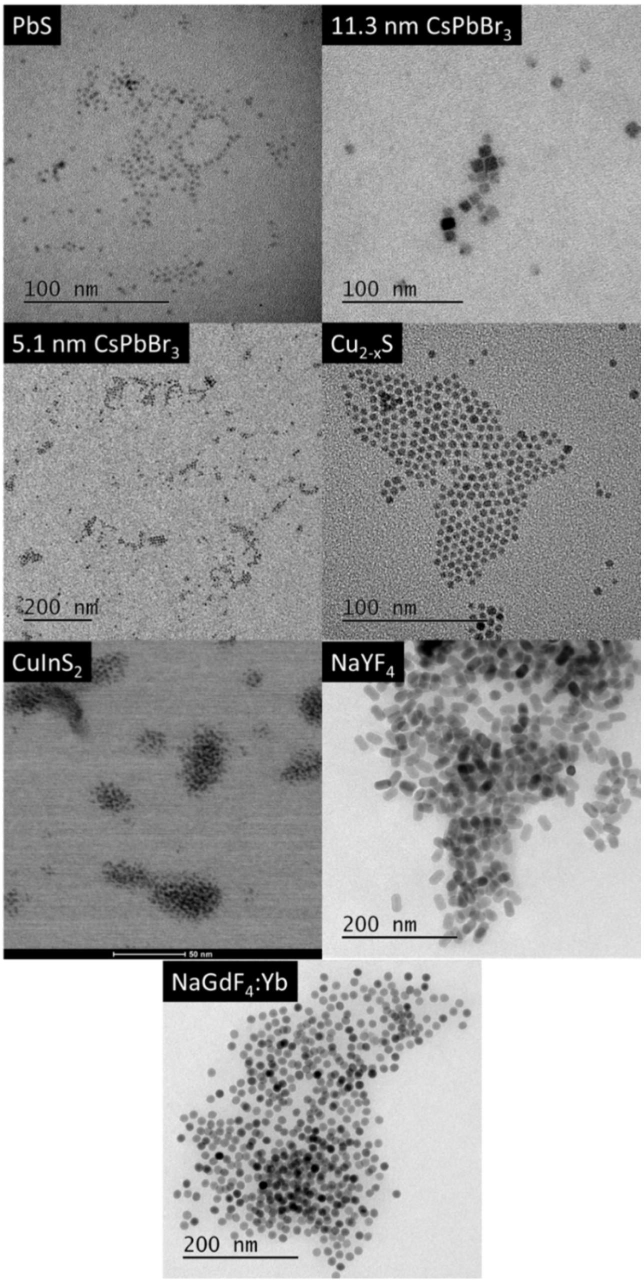

Figure S24. Representative TEM images of all nanocrystalline compositions after growth of the alumina shells to incorporate a high loading of PAH ligands. At the exception of $NaYF_4$, all the NCs preserve their initial shape. Furthermore, there is no evidence of homogeneously nucleated alumina.

Except from this sample, all NCs preserved excellent colloidal stability throughout the c-ALD process which was indicated both by the absence of scattering in the absorption spectra and by the lack of any large aggregates in the DOSY measurements. Thus, we conclude that the aggregation in some of the images is only apparent and due to the drying process and change of the surface chemistry

We note that the NC@AlOx samples, in particular $CsPbBr_3$ NCs, degraded rapidly under the electron beam. This phenomenon might derive from the larger ligand density surrounding the NCs as the organic matter readily decompose under the beam.

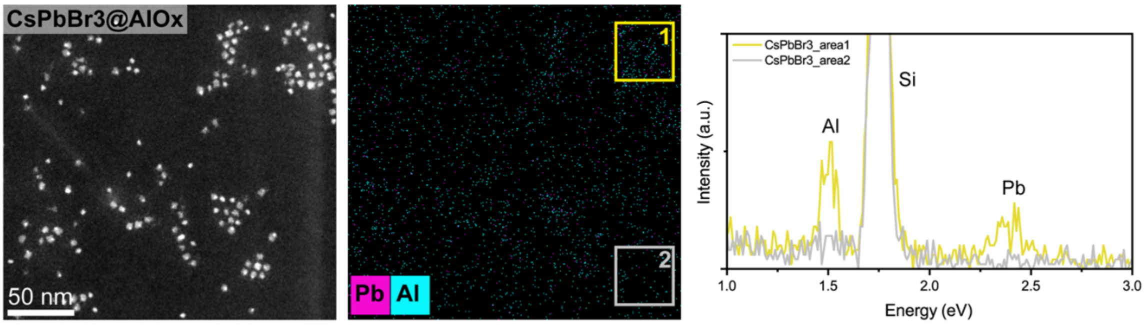

Figure S25: Representative $CsPbBr_3$@AlOx (CsPbBr3 size is 5.1 nm) HAADF-STEM images and the corresponding EDX spectra of the areas highlighted. We observe colocalization of the aluminium and Pb signal, suggesting that Al surrounds the $CsPbBr_3$ NCs.

Discussion on X-for-X exchange on $CsPbBr_3$:

$CsPbBr_3$ NCs, both 5.1 and 11.3 nm, showed the appearance of narrow resonances (~20 Hz) associated with dynamic 9-ACA (Figure S32, 33) upon ligand exchanges. Given the lack of stability of these NCs to antisolvent purification, an accurate estimate of bound 9-ACA cannot be achieved with optical absorption. Consequently, a significant fraction of the added 9-ACA is not tightly bound to the NC surface but in a dynamic equilibrium.

Similar experiments of addition of the ligand to the NC solution were performed with 1-PCA on the 5.1 nm $CsPbBr_3$ NCs (Figure S26). Through the titrations, the five resonances at 9.71, 8.92, 8.12, 7.98 and 7.85 ppm associated with 1-PCA were observed by ^1H NMR. These resonances were narrower then expected for dynamically passivated NCs, however two dimensional nuclear Overhauser effect spectroscopy (2D NOESY) suggested that 1-PCA interacts with the NCs through a dynamic passivation (See figure S27). Photoluminescence (PL) quenching of the 5.1 nm $CsPbBr_3$ NCs, when selectively exciting the NCs with 450 nm light, was observed and has been previously associated to triplet energy transfer from the NC to 1-PCA (Figure S45).[23] The average PL lifetime went from 4.1 to 1.8 ns upon addition of 1-PCA.

To separate free from bound 1-PCA, we performed an antisolvents purification with mixture of ethylacetate-acetone mixture (10% acetone, figure S43) or only acetone (Figure S44). These solvents were selected as they are aprotic and mild compared to alcohols. After only one purification the optical absorption of the NCs displayed a broader red shifted excitonic peak independently of the antisolvents employed (Figure S43, 44). The optical absorption measurements also pointed to a larger amount of 1-PCA to NCs in the solution, which could account for the dissolution of the $CsPbBr_3$ NCs (Figure S43, 44). ^1H NMR revealed the presence of free 1-PCA by noting the narrow linewidths, which confirms that = the excess 1-PCA is not bound and that further purification steps would be required to obtain solely bound 1-PCA (Figure S43). Therefore, our attempts to purify resulted in the dissolution of the desired NCs and the formation of larger structures. Time resolved PL measurements indicated a distinct quenching mechanism as compared to triplet energy transfer (Figure S43-44). Indeed, concurrent with the red shifted absorption, we consider that triplet energy transfer is less favorable.[23] This view is supported by the work of Luo et al, which suggests that quantum confinement is necessary for efficient triplet energy transfer.[23] Further analysis of the PL decay, indicate two primary contributions: a fast component that could be consistent with triplet energy transfer and a much longer component that is inconsistent with triplet energy transfer or the native radiative recombination channel of 5.1 nm $CsPbBr_3$ NC (Figure S43-44). Therefore, we associate its presence to the creation of trap states, which are known to form as a consequence of ligand loss during purification.

Finally, the same experiments were performed with 9-PTA. ^1H NMR revealed narrow linewidth resonances associated with 9-PTA (Figure S28). 2D NOESY showed that 9-PTA was dynamically interacting with the NC surface (Figure S29). Endothermic energy transfer from the 5.1 nm $CsPbBr_3$ NCs to 9-PTA was also observed (Figure S48). In all, nanocrystalline systems such as the $CsPbBr_3$ NCs employed will have dynamically binding PAH ligands resulting in potential parasitic absorption and hampered processability.

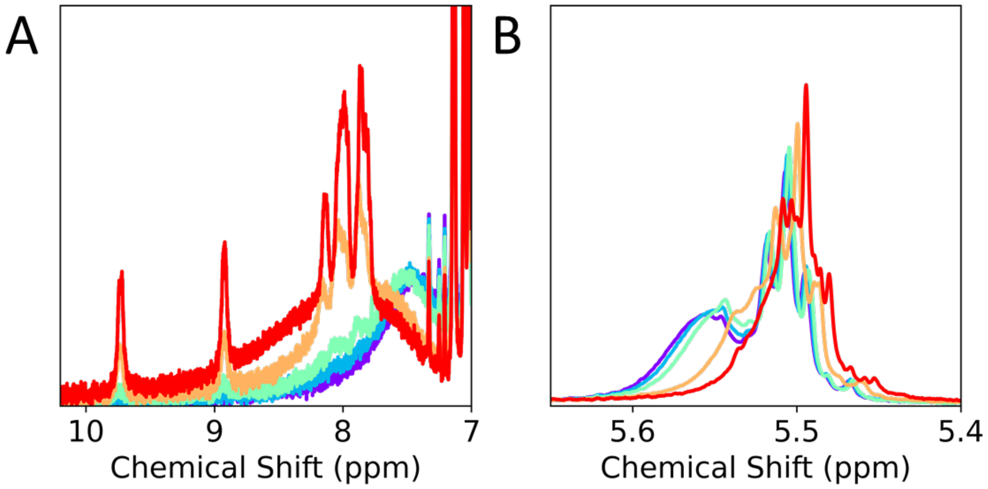

Figure S26: Ligand exchange with 1-PCA on 5.1 nm $CsPbBr_3$ NCs. A) As 1-PCA is introduced five new resonances are observed and are associated with a dynamic passivation (See Figure S50). The oleylamonium resonance is observed to shift to higher ppm as was previously noted when carboxylic acids are added to perovskite NCs. B) The alkene resonances arising from both OLAM and OLAC are seen to shift to lower ppm, indicating that are being displaced and are more dynamic.

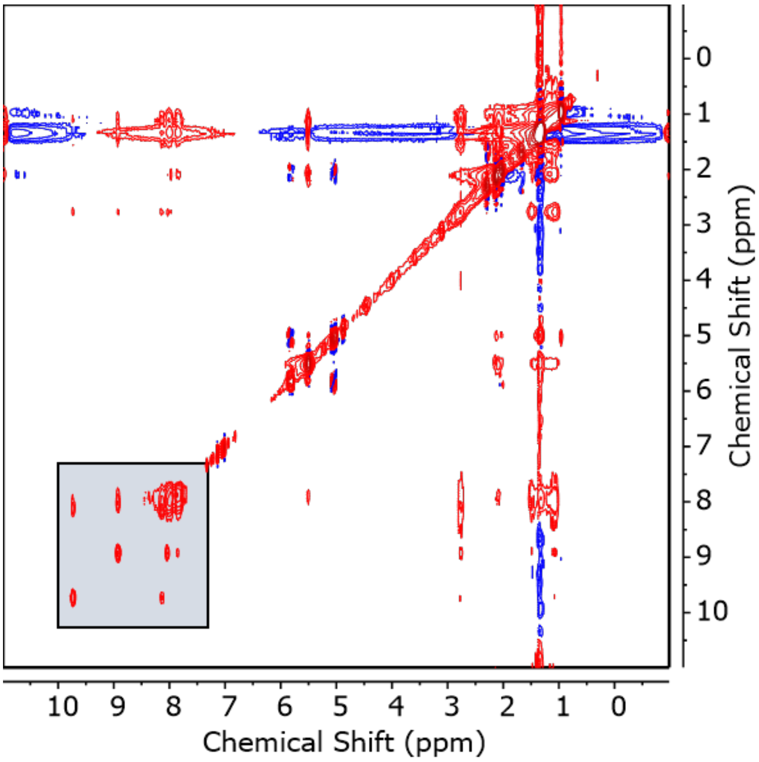

Figure S27: 2D NOESY plot of 5.1 nm $CsPbBr_3$ NCs with added 1-PCA. This experiment can reveal if ligands are interacting with the surface of the NCs by contrasting the sigh of the off-diagonal peaks with those on the diagonal. When the diagonal and off-diagonal peaks are of the same sign the ligands are bound or dynamically interacting with the NC surface. We observe, highlighted in the grey box, that the off diagonal and diagonal signal for 1-PCA are of the same sign (red) suggesting that 1-PCA is interacting with the NC surface.

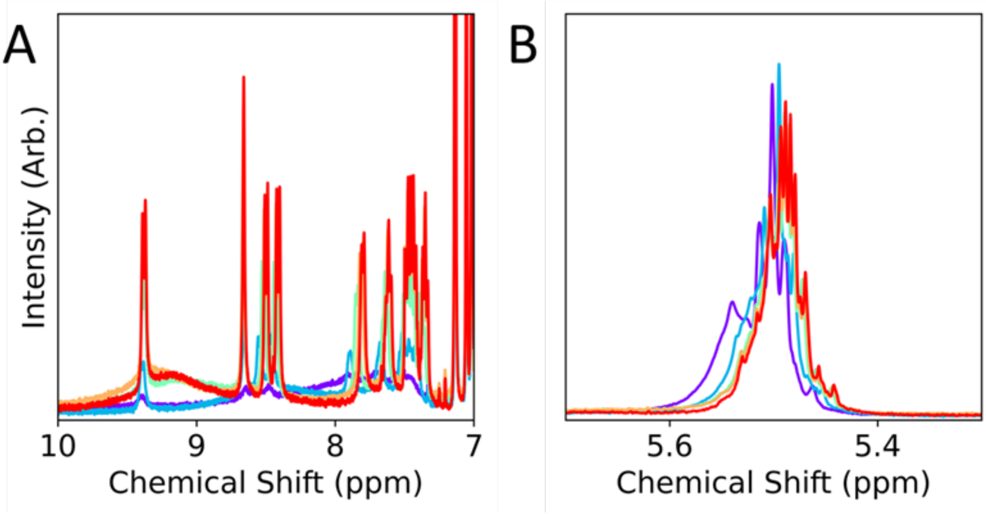

Figure S28: Ligand exchange with 9-PTA on 5.1 nm $CsPbBr_3$ NCs. A) As 9-PTA is introduced eight new resonances are observed and are associated with a dynamic passivation (See Figure S55). The oleylamonium resonance shifts to higher ppm as it was previously noted when carboxylic acids are added to perovskite NCs. B) The alkene resonances arising from both OLAM and OLAC shifts to lower ppm, indicating that they are being displaced and are more dynamic.

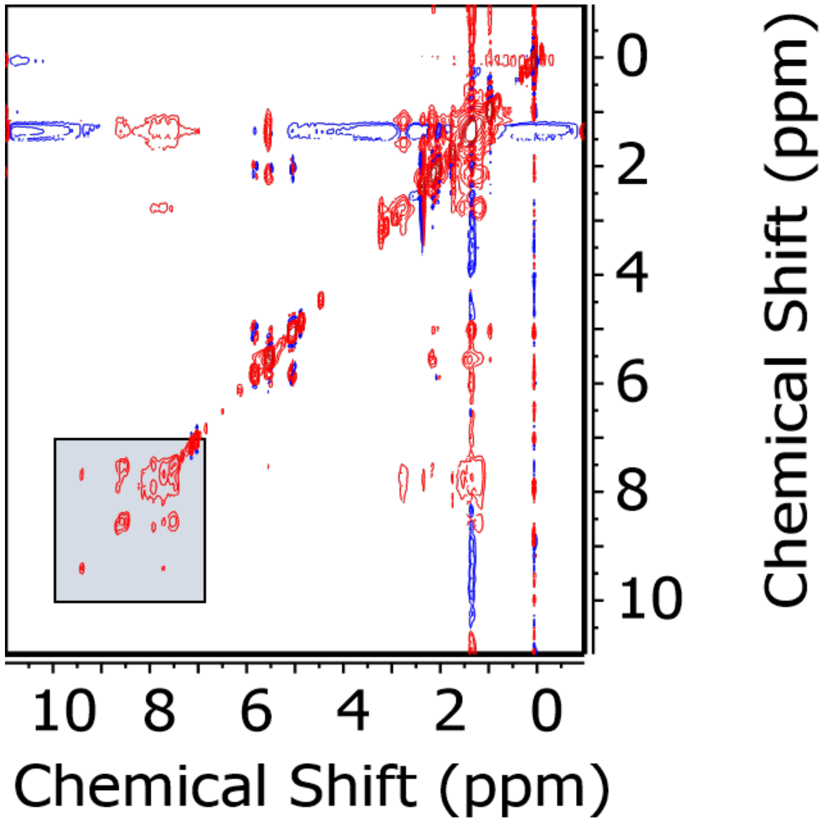

Figure S29: 2D NOESY plot of 5.1 nm 9-PTA/$CsPbBr_3$ NCs. We observe, highlighted in the grey box, that the off diagonal and diagonal signal for 9-PTA are of the same sign (red) suggesting that 9-PTA interacts with the NC surface.

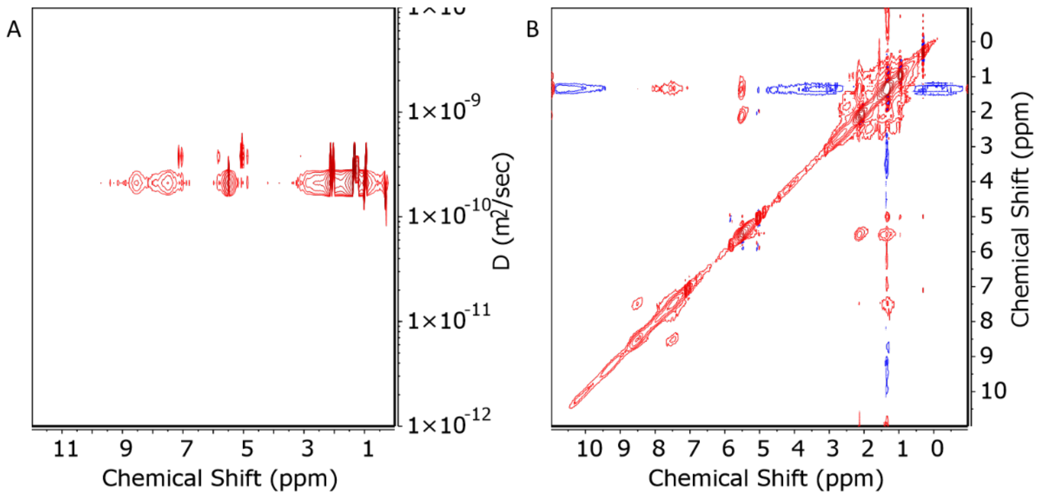

Figure S30: DOSY (A) and 2D NOESY (B) of 5.1 nm 9-PTA/$CsPbBr_3$@AlOx. DOSY reveals one diffusion coefficient for all ligands. 2D NOESY shows cross peaks of the same sign as the diagonal in the 9-PTA region suggesting 9-PTA is bound.

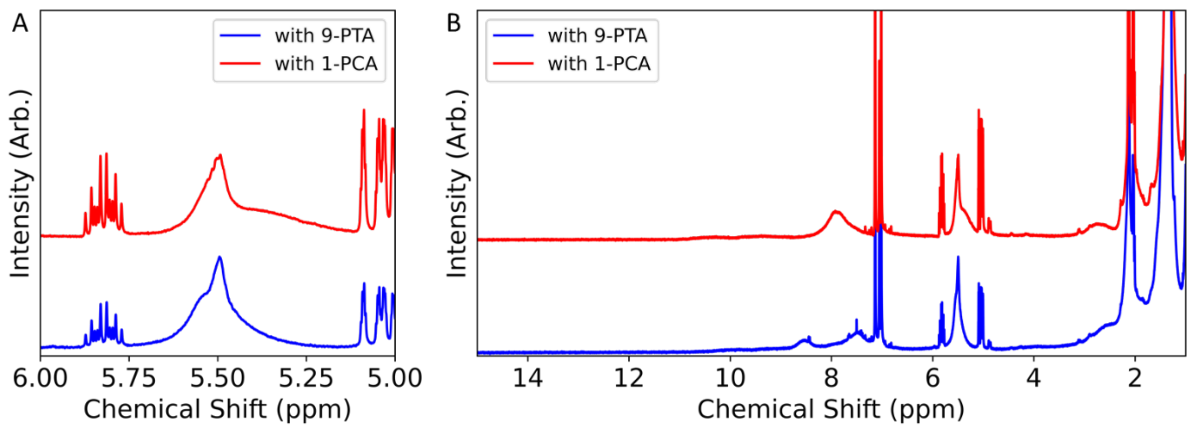

Figure S31: Alkene region (A) and full (B) ^{1}H NMR of 5.1 nm PAH/$CsPbBr_3$@AlO_x NCs (PAH: 9-PTA in red and 1-PCA in blue). A narrower contribution is observed in the alkene region. We postulate that this signal originates from the native oleylamonium ligands, possibly in the form of oleylamine.

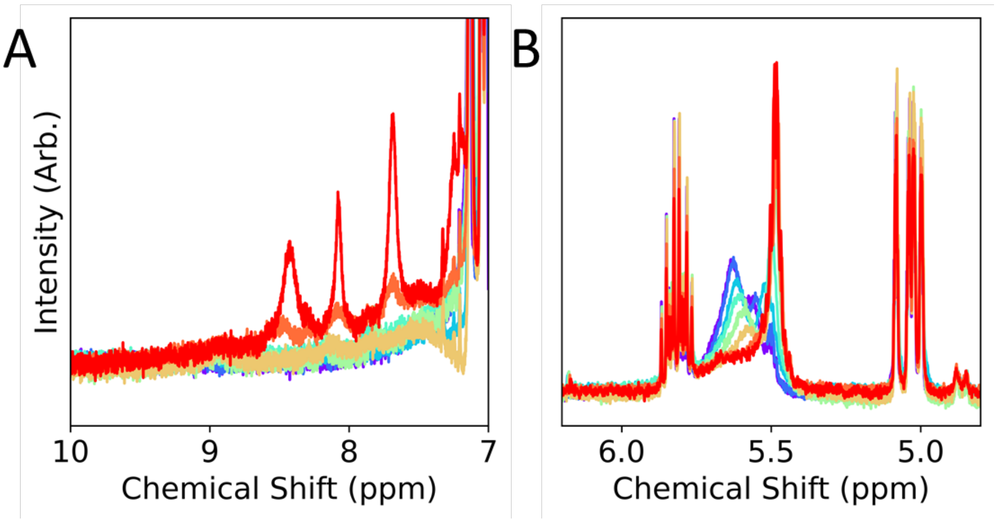

Figure S32: Titration of 9-ACA to 11.3 nm $CsPbBr_3$ NCs. A) The aromatic region showing dynamic binding of 9-ACA as more equivalences are added. B) Alkene regions showing the displacement of native ligand as more 9-ACA is added.

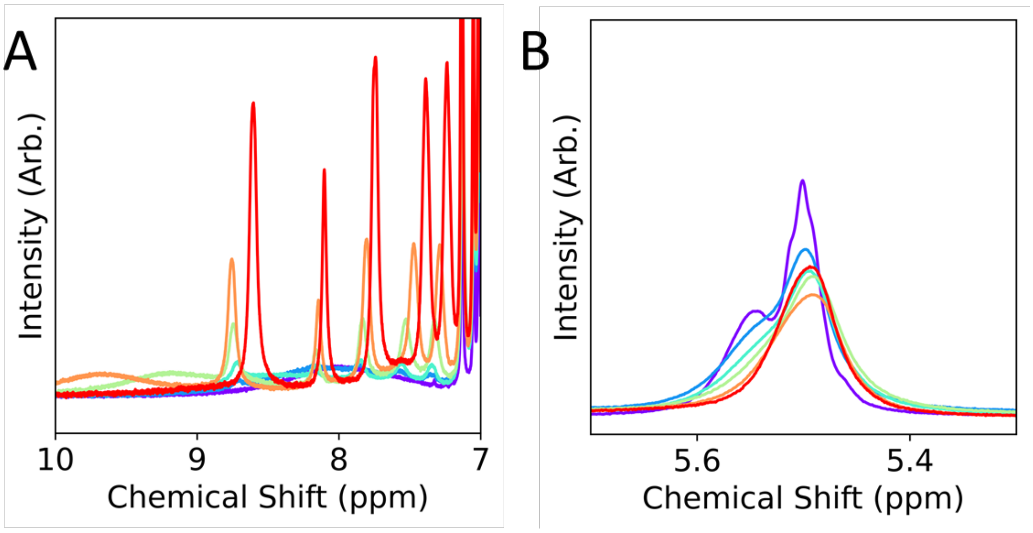

Figure S33: Ligand exchange with 9-ACA on 5.1 nm $CsPbBr_3$ NCs. A) Initially the broad oleylamonium resonance (purple) is at 8 ppm. As 9-ACA is introduced five new resonances are observed and are associated with a dynamic passivation due to the narrow linewidths of ~ 20 Hz. The oleylamonium resonance shifts to higher ppm as it was previously noted when carboxylic acids are added to perovskite NCs. B) The alkene resonances arising from both OLAM and OLAC shift to lower ppm, indicating that they are being displaced and are more dynamic.

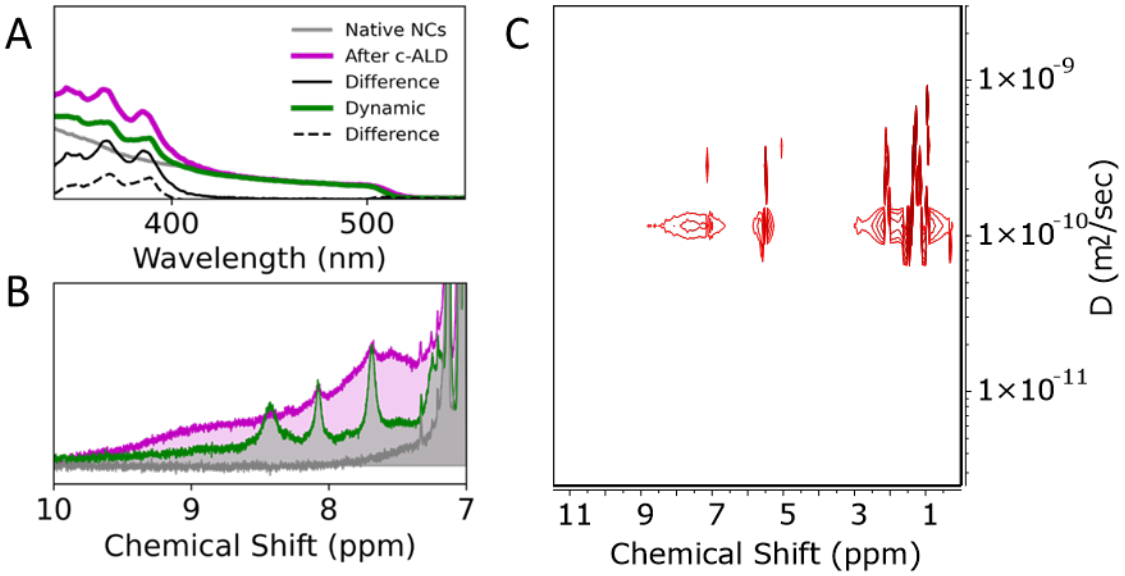

Figure S34: All the added 9-ACA is bound on 11 nm $CsPbBr_3$ NCs after c-ALD in the 9-ACA/ $CsPbBr_3$@AlOx sample. A) Optical absorption of the native sample (grey), with added 9-ACA (green) and after c-ALD (purple). The corresponding ^{1}H NMR are shown in (B). C) After c-ALD all ligands (OLAC and 9-ACA) diffuse with a similar diffusion coefficient as measured by DOSY, suggesting that all the ligands are travelling as a unit.

Discussion on X-for-X exchange on $Cu_{2-x}S$ and $CuInS_2$:

9-ACA does not bind to $Cu_{2-x}S$ NCs when conventional ligand exchange is performed This conclusion is revealed by the narrow linewidths of 9-ACA (5 to 10 Hz) and DOSY measurements which confirm that 9-ACA was not interacting with the NC surface (Figure S36, S37). This finding is consistent with previous observations that carboxylates cannot penetrate thiolated surfaces.[24,25] 9-ACA could be added to $CuInS_2$ NCs, at low equivalences, which was observed with a shift of the OLAM alkene resonances towards free OLAM (Figure S35). However, as the titration progressed the 9-ACA resonance resembled that of free 9-ACA, similar to $Cu_{2-x}S$ NCs. Purifications of this sample by antisolvent purification resulted in the complete loss of 9-ACA (Figure S42). The OLAM signal was also lost, suggesting 9-ACA and OLAM weakly interacts with the surface.

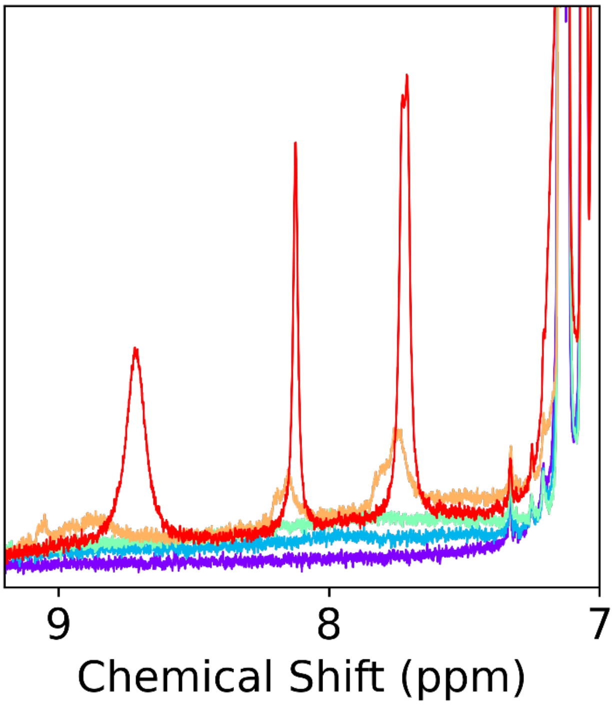

Figure S35: Ligand exchange with 9-ACA on $CuInS_2$ NCs. As 9-ACA is introduced three new resonances are observed. These linewidths range from ~30 Hz for the resonance at 8.72 ppm and ~10 Hz for the other two. These results indicate a certain level of interaction of 9-ACA with the NC, however, as seen in figure S42 the interaction is very week and purified NCs do not contain any 9-ACA.

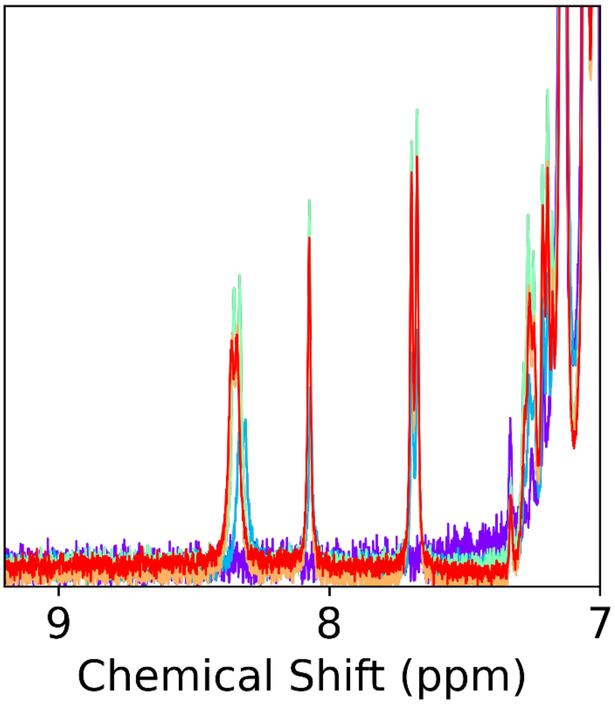

Figure S36: Ligand exchange with 9-ACA on $Cu_{2-x}S$ NCs. As 9-ACA is introduced three new resonances are observed. The narrow linewidth (~ 5 *to* 10 Hz) are associated with free (non-interacting) 9-ACA.

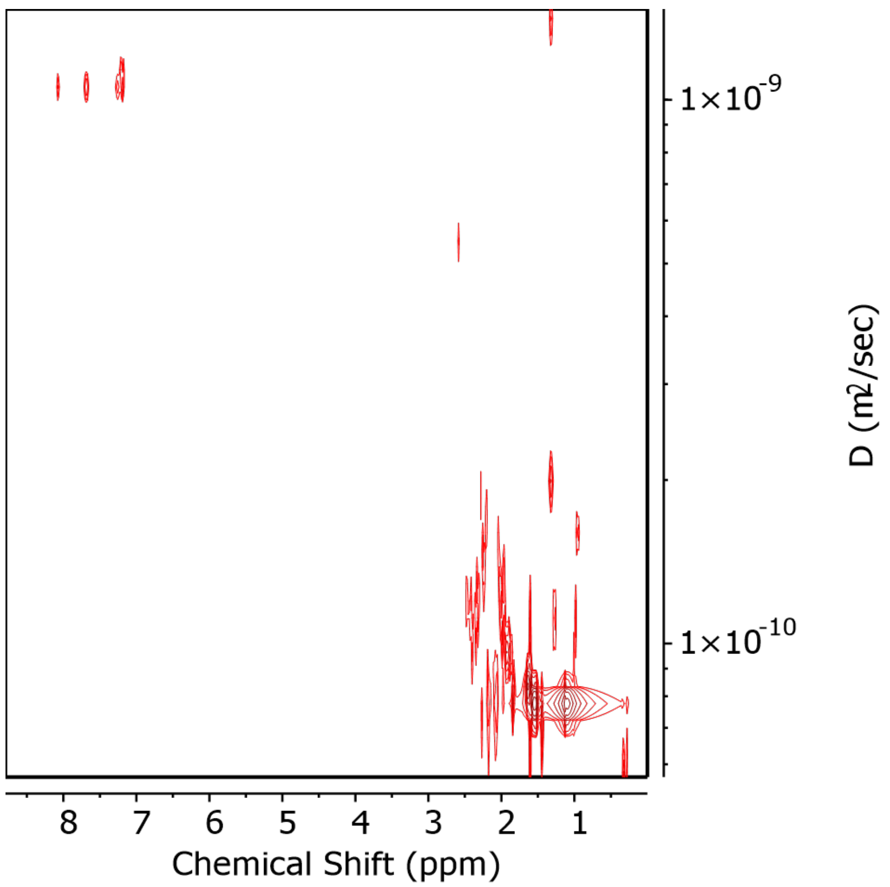

Figure S37: DOSY acquired for a sample of $Cu_{2-x}S$ NCs with added 9-ACA. DOSY suggest through the diffusion coefficient that 9-ACA is not interacting with the NC surface.

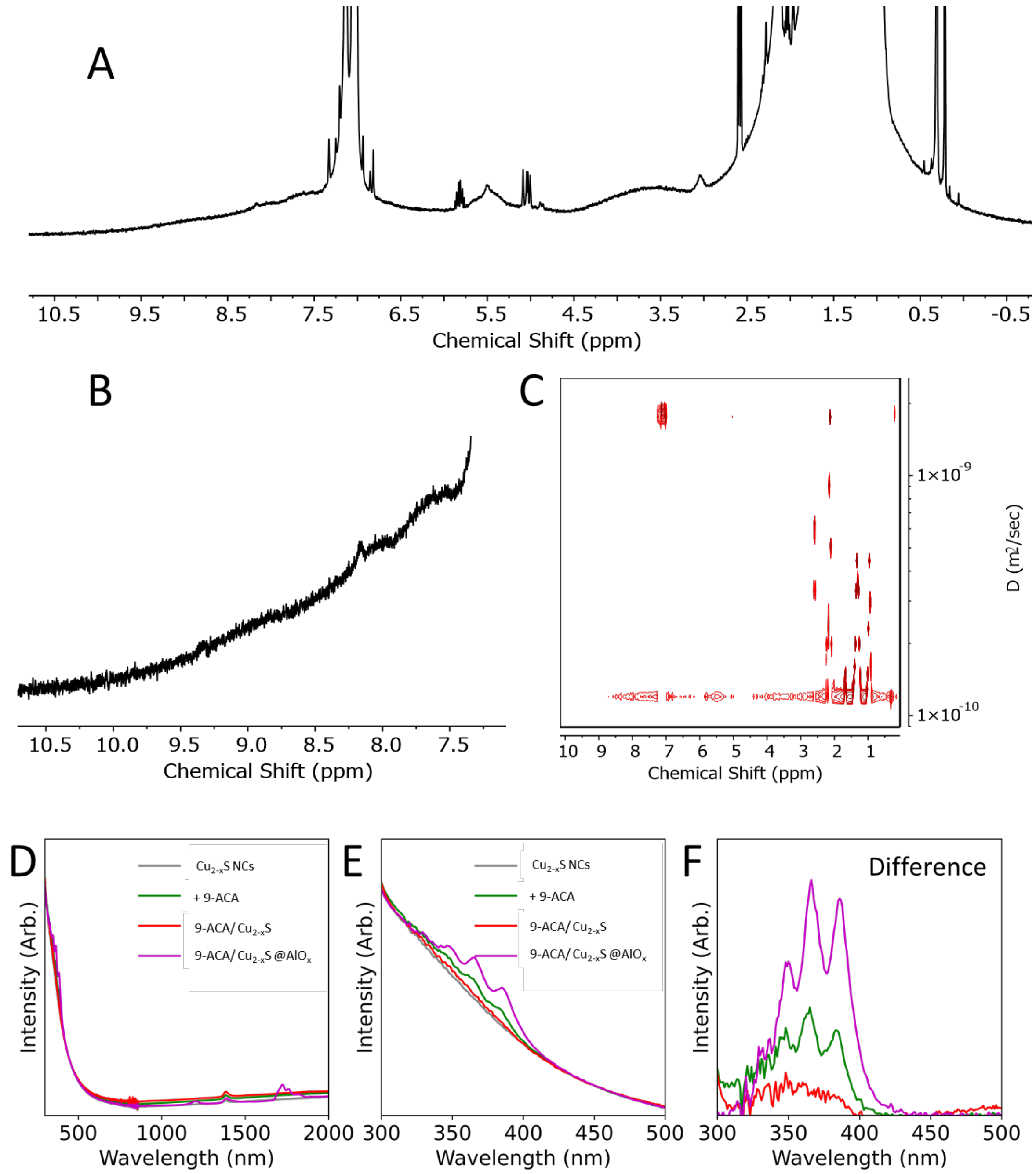

Figure S38: A) ^{1}H NMR of 9-ACA/$Cu_{2-x}S$@AlOx. B) Zoom in the aromatic region showing the broadened 9-ACA resonances. C) DOSY shows a similar diffusion coefficient for 9-ACA, OLAC and the native DDT. These results confirm that 9-ACA diffuses with the NC. D) Complete optical absorption of the native (grey) $Cu_{2-x}S$ NCs, with added 9-ACA (green) and then purified (red) and then after c-ALD to incorporate 9-ACA. E) Zoom in on the optical signal from 9-ACA. F) Difference spectrum, from which we subtract the native spectrum. The direct addition of 9-ACA does not result in binding as antisolvent purification completely remove the 9-ACA. In contrast, c-ALD incorporates 9-ACA within the shell.

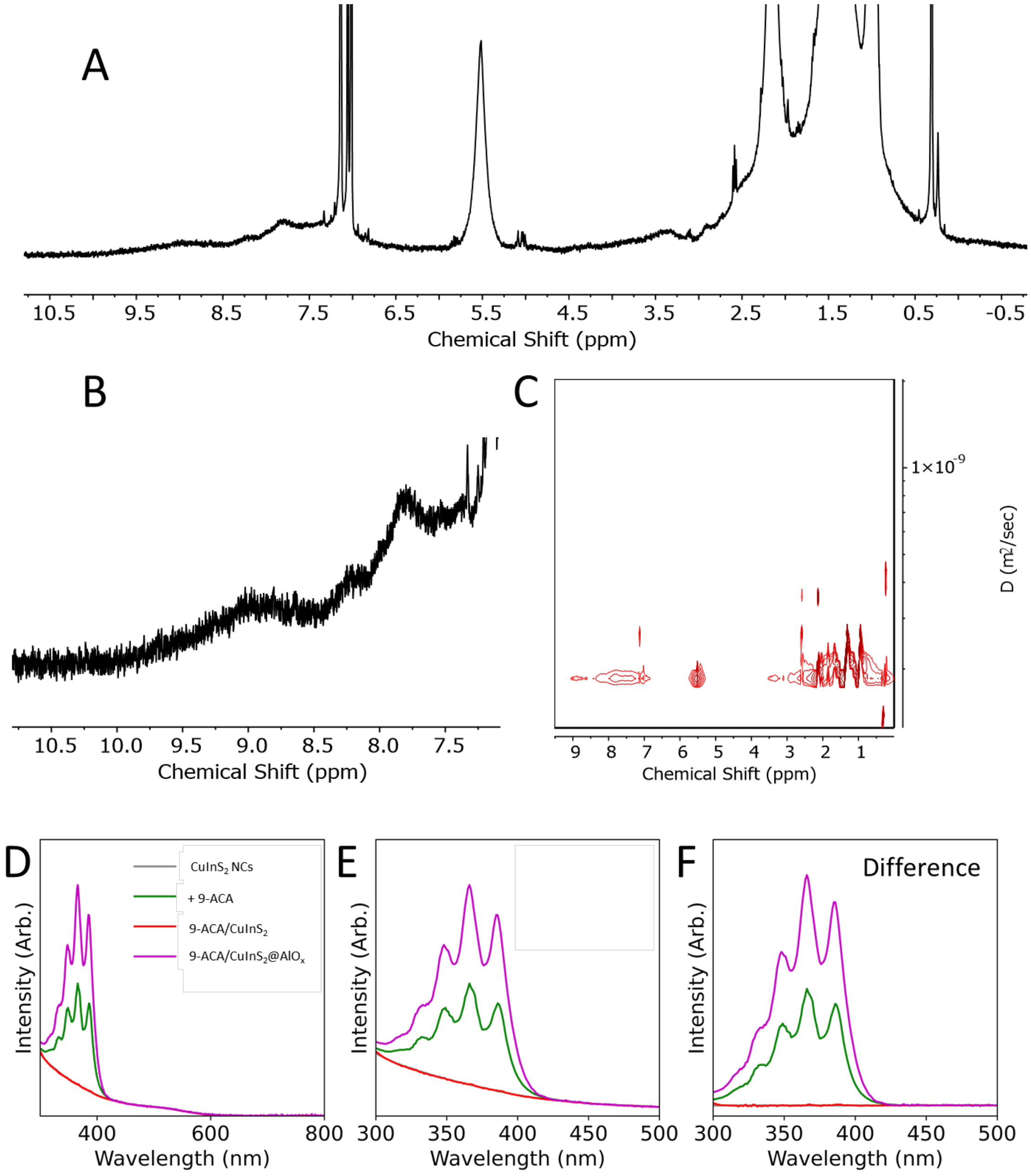

Figure S39: A) ^{1}H NMR of 9-ACA/$CuInS_2$ @AlOx. B) Zoom in the aromatic region showing the broadened 9-ACA resonances. C) DOSY shows a similar diffusion coefficient for 9-ACA, OLAC and the native DDT. These results confirm that 9-ACA is diffusing with the NC. D) Complete optical absorption of the native (grey) $CuInS_2$ NCs, with added 9-ACA (green) and then purified (red) and then after c-ALD to incorporate 9-ACA. E) Zoom in on the optical signal from 9-ACA. F) Difference spectrum, from which we subtract the native spectrum. The direct addition of 9-ACA does not result in binding as antisolvent purification causes complete loss of 9-ACA. In contrast, c-ALD incorporates 9-ACA within the shell.

Discussion on X-for-X exchange on $NaYF_4$:

The exchange on $NaYF_4$ NCs with 9-ACA revealed a dynamic passivation of 9-ACA similar to that of PbS NCs (Figure S22). Further, the alkene resonance of OLAC became resolvable confirming an exchange with 9-ACA (Figure S40). Monitoring of these exchanges could not be performed on $NaGdF_4$:Yb NCs as the paramagnetic nature of the Gd^{3+} ions in the NC core broadened all the proton resonances (Figure S5).

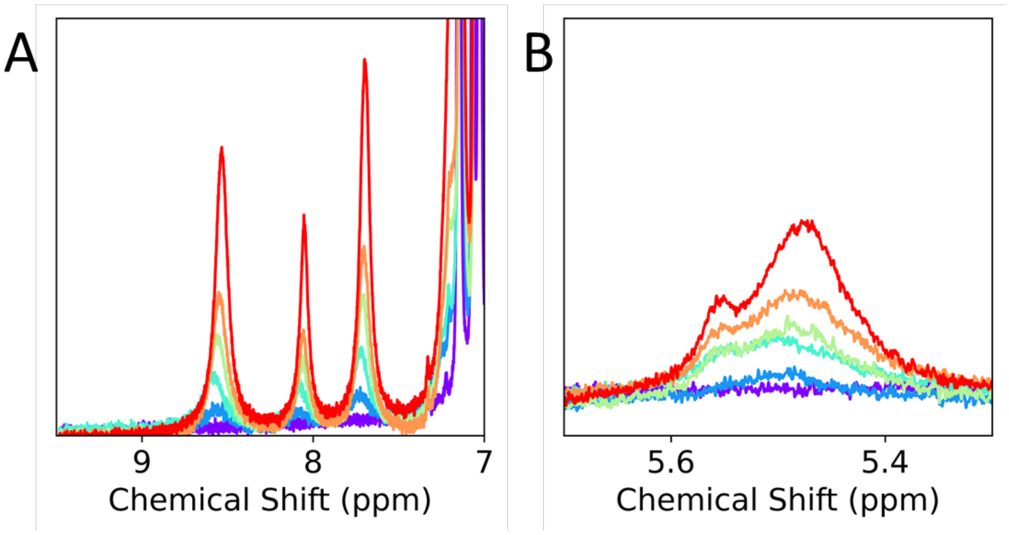

Figure S40: Ligand exchange with 9-ACA on $NaYF_4$ NCs. A) As 9-ACA is introduced three new resonances are observed and are associated with a dynamic passivation due to the narrow linewidths of ~ 20 Hz. B) As more 9-ACA is added, an alkene signal rises from the background and is consistent with the release of native OLAC.

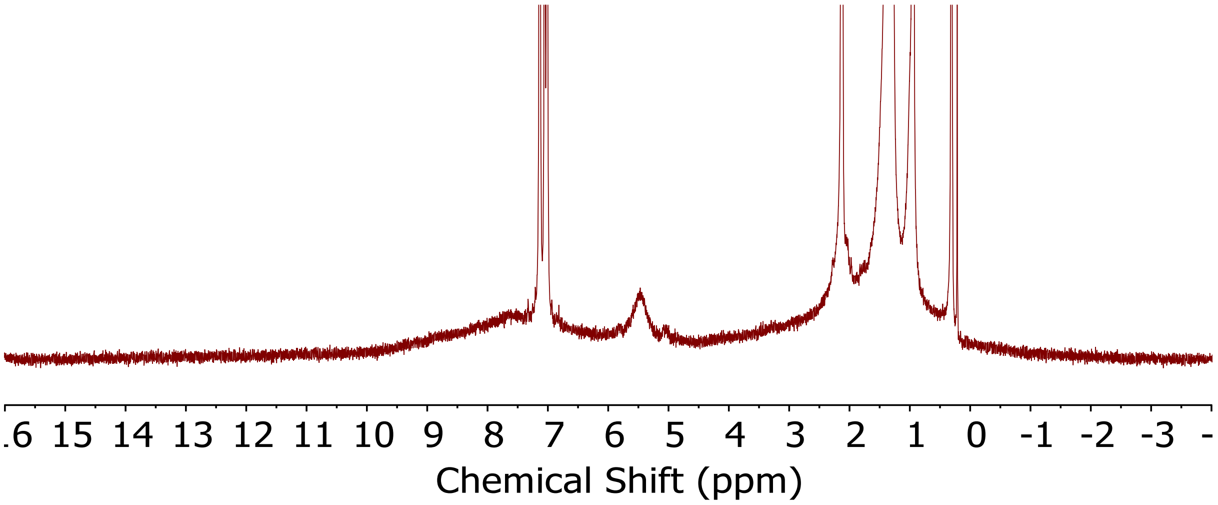

Figure S41: ^{1}H NMR of $NaYF_4$ NCs after undergoing c-ALD to incorporate 9-ACA. The broad resonance of 9-ACA between 10 and 7 ppm is observed suggesting all 9-ACA is bound.

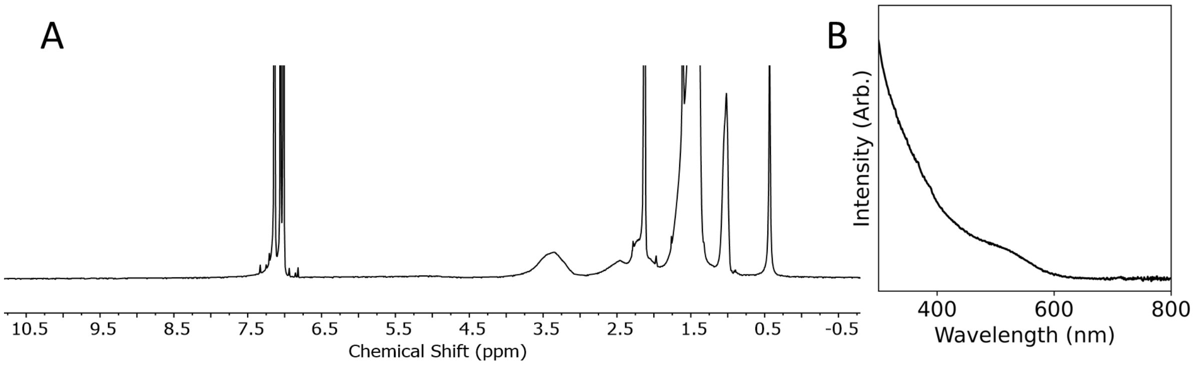

Figure S42: Purifying $CuInS_2$ NCs exposed to 9-ACA. After purification so 9-ACA is not observed by (A) ^{1}H NMR or (B) optical absorption.

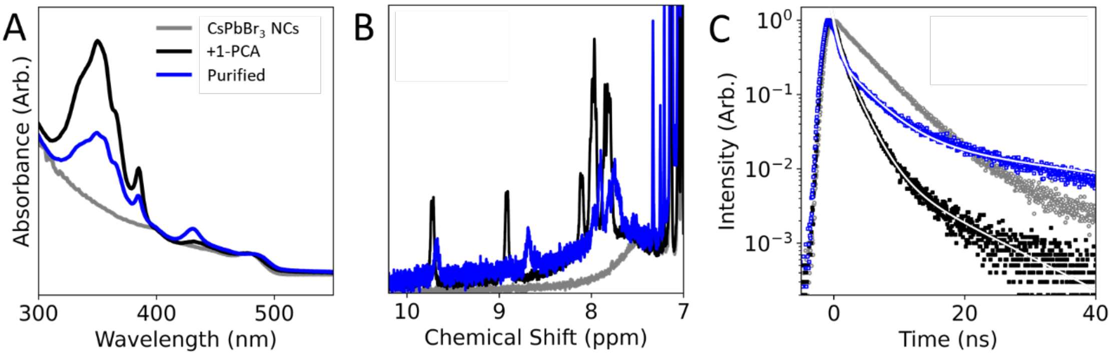

Figure 43: Purification of 5.1 nm CsPbBr3 with added 1-PCA using ethyl acetate as the antisolvent. A) A red shifted absorption is observed indicating the formation of larger NCs. Remaining 1-PCA is observed and ^{1}H NMR (B) evidences that it is not fully bound. C) TRPL suggest that trap states are formed during antisolvent purification.

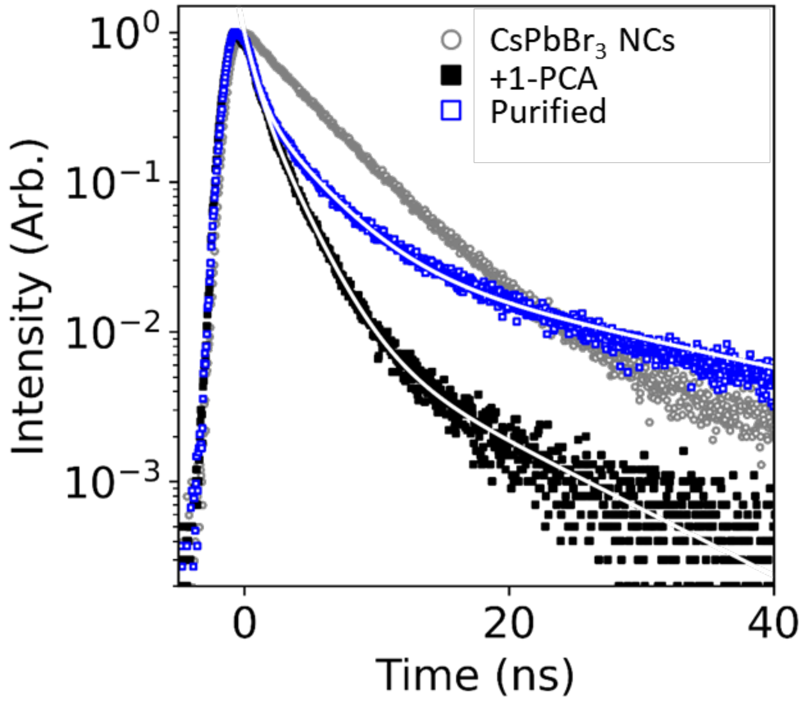

Figure 44: Purification of 5.1 nm CsPbBr3 with added 1-PCA using acetone as the antisolvent. The main data are presented in Figure 4C. We highlight it here again such that comparison can be done with purifications using ethyl acetate. Both decays after purification (blue) show similar behaviors, indicative of trap formation due to the loss of ligands.

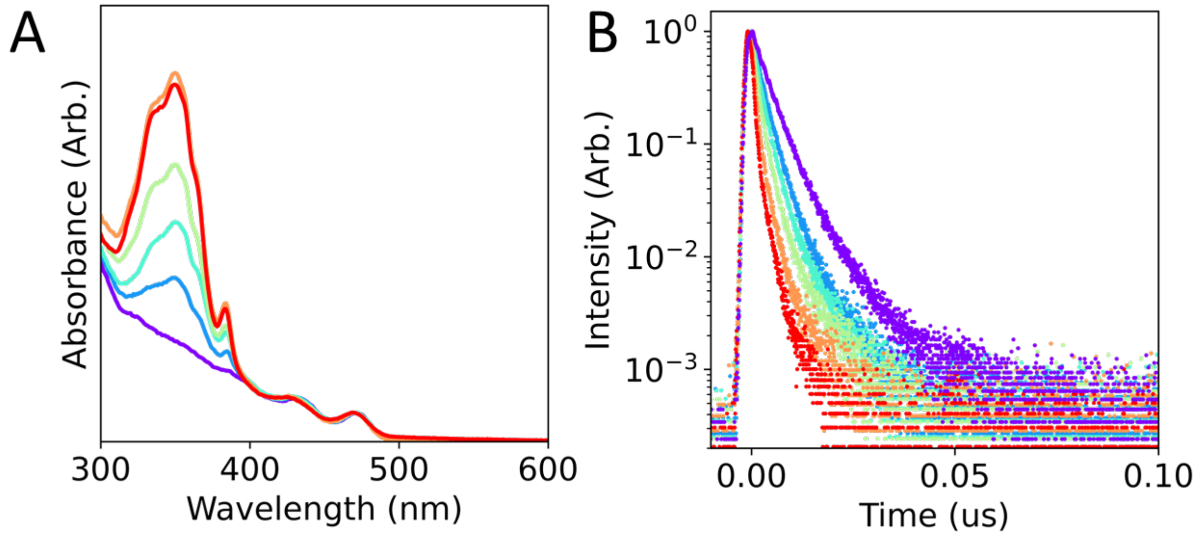

Figure S45: A) Optical absorption and B) time resolved photoluminescence (TRPL) of 5.1 nm $CsPbBr_3$ (450 nm excitation) with variable amounts of 1-PCA. The optical absorption of 1-PCA grows on top of that of the NCs. Concomitantly, the TRPL decay with selective excitation of only the NCs shows quenching indicative of triplet energy transfer, which becomes more pronounced as more 1-PCA molecules are added.[23]l

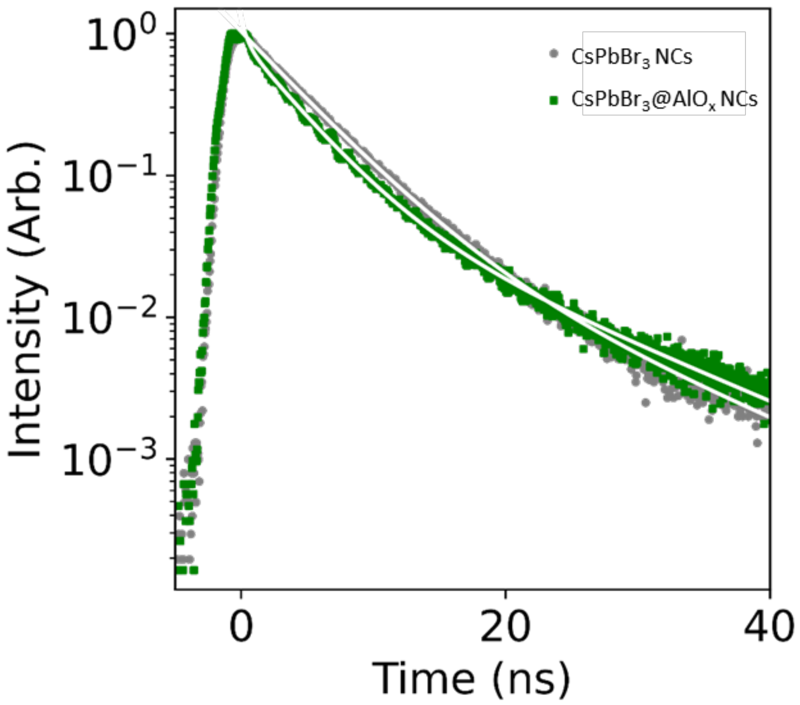

Figure S46: TRPL decay of the as-synthesized $CsPbBr_3$ NCs and $CsPbBr_3@AlOx$ showing that the alumina shell does not significantly alter the TRPL of the 5.1 nm $CsPbBr_3$ NCs.

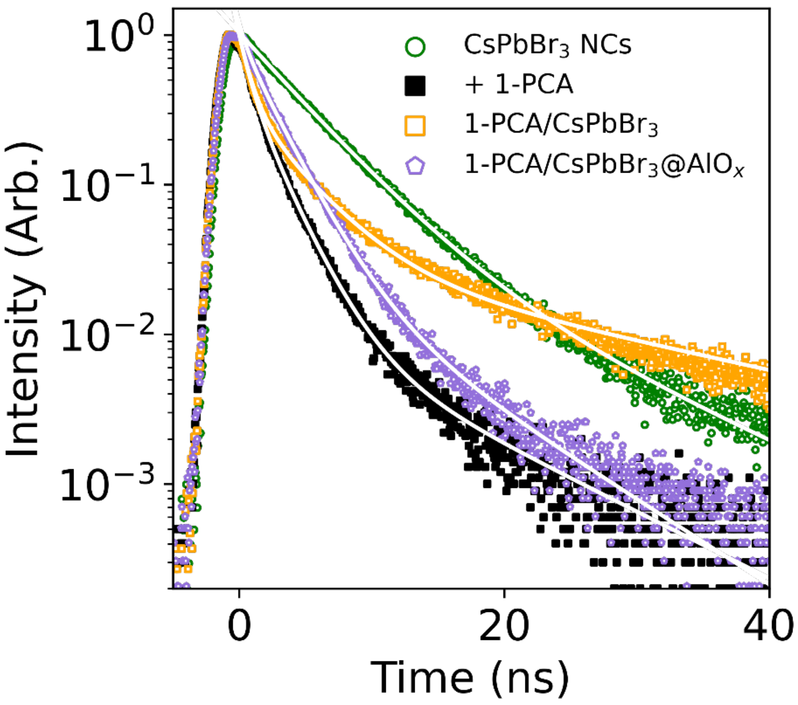

Figure S47: Comparing the quenching ability of 1-PCA on $CsPbBr_3$ NC. Time resolved photoluminescence reveals the quenching of the as-synthesized sample (green) when adding 1-PCA (black). These data indicate triplet energy transfer from the NC to 1-PCA. Antisolvent purification results in the lost of this quenching behavior and the emergence of a long lifetime component indicative of trap formation (orange). Quenching can be preserved when performing c-ALD, although is not as efficient as the direct exposure to 1-PCA because the shell increases the physical distance between the NC surface and the 1-PCA.

Table 2: Summary of the time-resolved photoluminescence decays

	A	t	B	t	C	t	Average
Native	0.96	4.27	0.07	11.03			4.1
+ c-ALD	0.80	3.62	0.09	11.10	0.40	0.40	3.7
+ 1-PCA	0.48	2.16	0.014	9.75	0.50	0.75	1.8
Purified	0.33	3.72	0.03	22.5	0.63	0.79	7.1
+ c-ALD +1-PCA	0.87	2.38	0.04	7.63	0.28	0.56	2.0

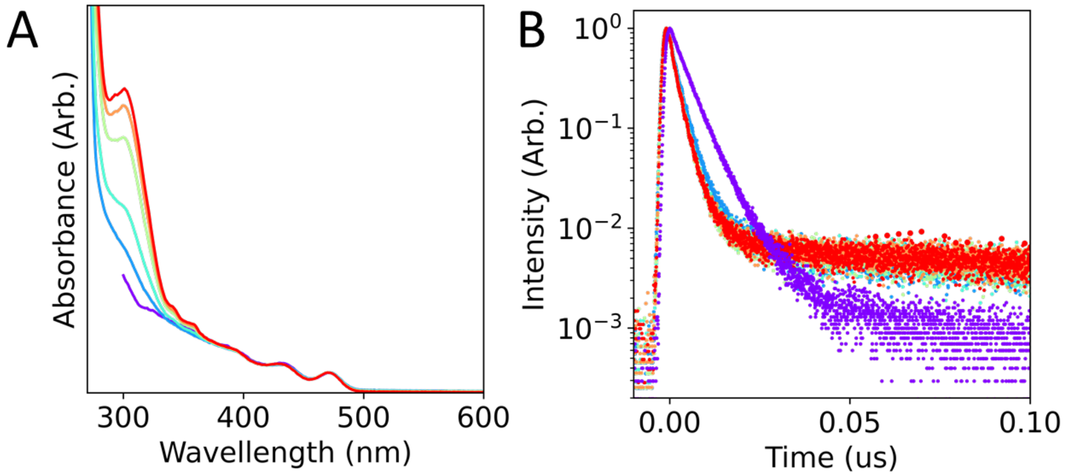

Figure S48: A) Optical absorption and B) time resolved photoluminescence (TRPL) of 5.1 nm $CsPbBr_3$ (450 nm excitation) with variable amounts of 9-PTA. The optical absorption of 9-PTA grows on top of that of the NCs as the amount of 9-PTA increases. Concomitantly, the TRPL decay with selective excitation of only the NCs shows initial quenching, indicative of triplet energy transfer, and a long-time component, consistent with reverse triplet energy transfer back to the NC.[26,27]

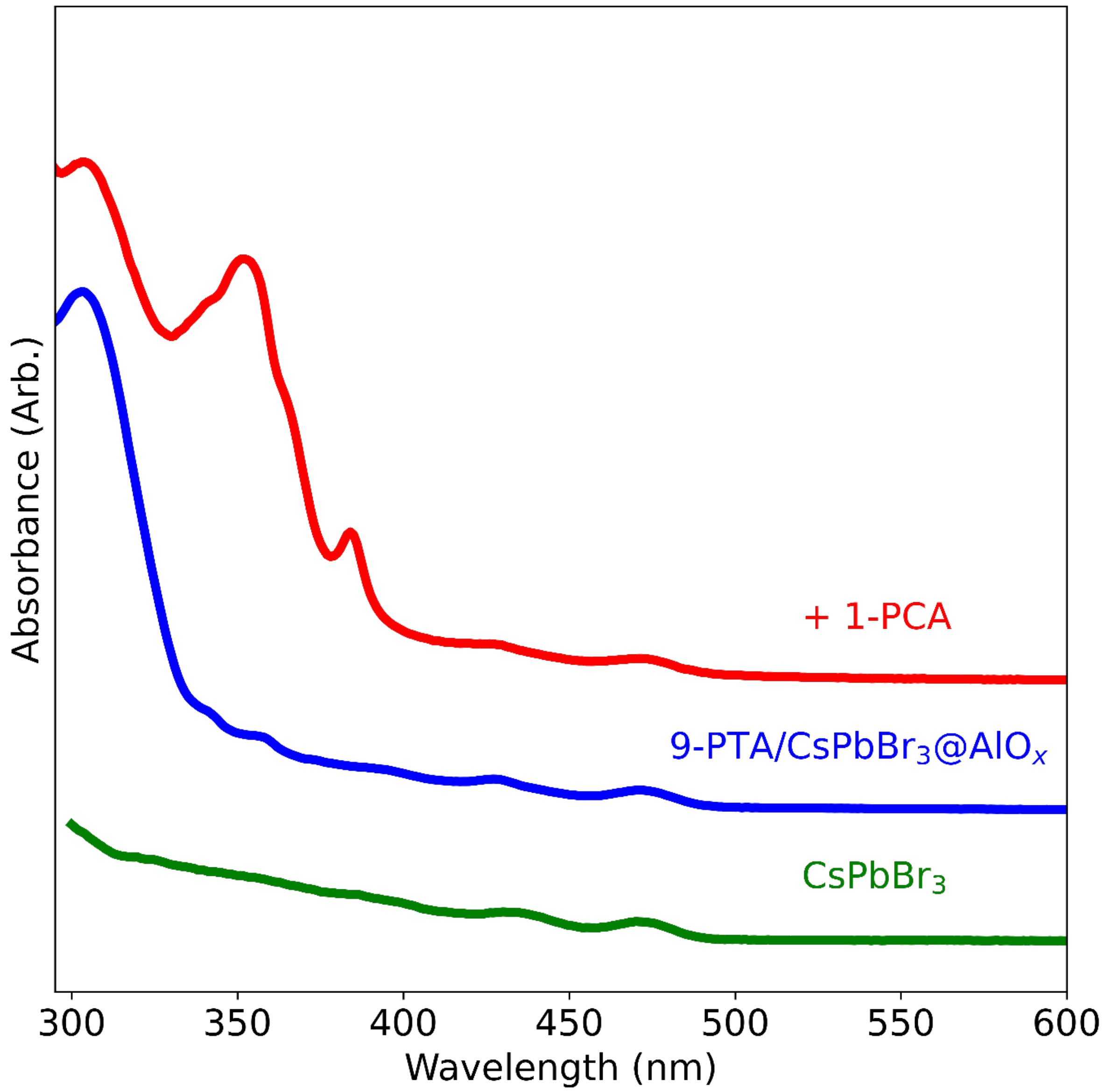

Figure S49: Optical absorption spectrum of the native 5.1 nm $CsPbBr_3$ NCs (green), after undergoing c-ALD to incorporate 9-PTA (9-PTA/ $CsPbBr_3@AlO_x$, blue) and after the addition of 1-PCA (red). The optical signatures of 9-PTA and 1-PCA emerge a top that of the native NCs. The complete process does not alter the shape of the NCs as observed through the unchanged excitonic peak.

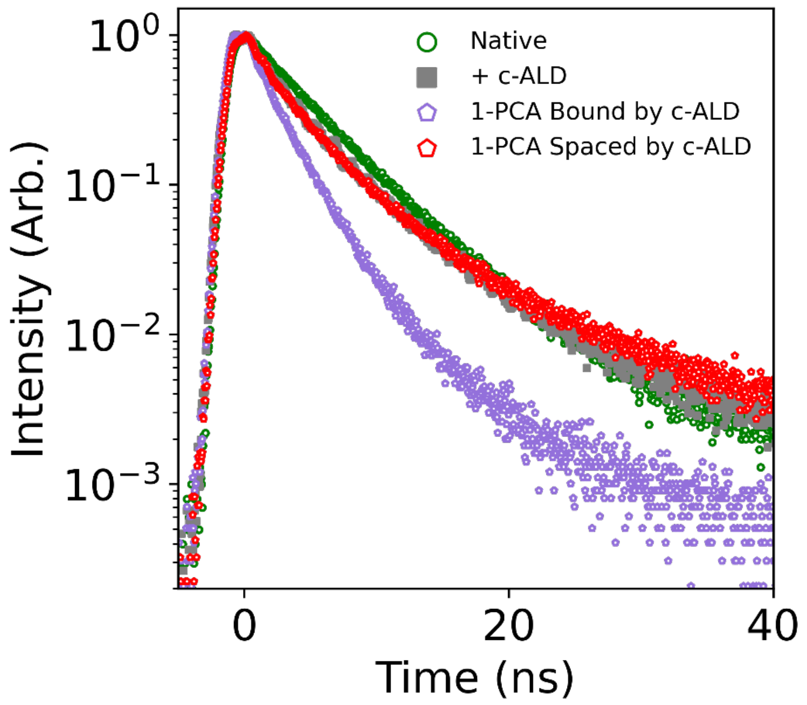

Figure S50: When 1-PCA is added in solution before alumina growth, the shell traps it directly on the NCs. In this case, efficient triplet transfer is observed (purple). If a shell is initially grown and then the 1-PCA is added, the shell creates a distance between the NC surface and the chromophore (approximately 0.5nm per 3 cycles). In this case, no energy transfer is observed (red). Indeed, the TRPL of such samples is very similar to one having solely undergone the growth of alumina.

References

(1) Green, P. B.; Narayanan, P.; Li, Z.; Sohn, P.; Imperiale, C. J.; Wilson, M. W. B. Controlling Cluster Intermediates Enables the Synthesis of Small PbS Nanocrystals with Narrow Ensemble Line Widths. *Chem. Mater.* **2020**, *32* (9), 4083–4094. https://doi.org/10.1021/acs.chemmater.0c00984.

(2) Hines, M. A.; Scholes, G. D. G. D. Colloidal PbS Nanocrystals with Size-Tunable Near-Infrared Emission: Observation of Post-Synthesis Self-Narrowing of the Particle Size Distribution. *Adv. Mater.* **2003**, *15* (21), 1844–1849. https://doi.org/10.1002/adma.200305395.

(3) Protesescu, L.; Yakunin, S.; Bodnarchuk, M. I.; Krieg, F.; Caputo, R.; Hendon, C. H.; Yang, R. X.; Walsh, A.; Kovalenko, M. V. Nanocrystals of Cesium Lead Halide Perovskites (CsPbX 3 , X = Cl, Br, and I): Novel Optoelectronic Materials Showing Bright Emission with Wide Color Gamut. *Nano Lett.* **2015**, *15* (6), 3692–3696. https://doi.org/10.1021/nl5048779.

(4) Dong, Y.; Qiao, T.; Kim, D.; Parobek, D.; Rossi, D.; Son, D. H. Precise Control of Quantum Confinement in Cesium Lead Halide Perovskite Quantum Dots via Thermodynamic Equilibrium. *Nano Lett.* **2018**, *18* (6), 3716–3722. https://doi.org/10.1021/acs.nanolett.8b00861.

(5) Han, S.; Deng, R.; Gu, Q.; Ni, L.; Huynh, U.; Zhang, J.; Yi, Z.; Zhao, B.; Tamura, H.; Pershin, A.; Xu, H.; Huang, Z.; Ahmad, S.; Abdi-Jalebi, M.; Sadhanala, A.; Tang, M. L.; Bakulin, A.; Beljonne, D.; Liu, X.; Rao, A. Lanthanide-Doped Inorganic Nanoparticles Turn Molecular Triplet Excitons Bright. *Nature* **2020**, *587* (7835), 594–599. https://doi.org/10.1038/s41586-020-2932-2.

(6) Saldanha, P. L.; Brescia, R.; Prato, M.; Li, H.; Povia, M.; Manna, L.; Lesnyak, V. Generalized One-Pot Synthesis of Copper Sulfide, Selenide-Sulfide, and Telluride-Sulfide Nanoparticles. *Chem. Mater.* **2014**, *26* (3), 1442–1449. https://doi.org/10.1021/cm4035598.

(7) Zhong, H.; Zhou, Y.; Ye, M.; He, Y.; Ye, J.; He, C.; Yang, C.; Li, Y. Controlled Synthesis and Optical Properties of Colloidal Ternary Chalcogenide CuInS2 Nanocrystals. *Chem. Mater.* **2008**, *20* (20), 6434–6443. https://doi.org/10.1021/cm8006827.

(8) Moreels, I.; Lambert, K.; Smeets, D.; Muynck, D. De; Nollet, T.; Martins, C.; Vanhaecke, F.; Delerue, C.; Allan, G.; Hens, Z. Size-Dependent Optical Properties of Colloidal PbS Quantum Dots. *ACS Nano* **2009**, *3* (10), 3023–3030.

(9) De Roo, J.; Ibáñez, M.; Geiregat, P.; Nedelcu, G.; Walravens, W.; Maes, J.; Martins, J. C.; Van Driessche, I.; Kovalenko, M. V.; Hens, Z. Highly Dynamic Ligand Binding and Light Absorption Coefficient of Cesium Lead Bromide Perovskite Nanocrystals. *ACS Nano* **2016**, *10* (2), 2071–2081. https://doi.org/10.1021/acsnano.5b06295.

(10) De Roo, J.; Yazdani, N.; Drijvers, E.; Lauria, A.; Maes, J.; Owen, J. S.; Van Driessche, I.; Niederberger, M.; Wood, V.; Martins, J. C.; Infante, I.; Hens, Z. Probing Solvent-Ligand Interactions in Colloidal Nanocrystals by the NMR Line Broadening. *Chem. Mater.* **2018**, *30* (15), 5485–5492. https://doi.org/10.1021/acs.chemmater.8b02523.

(11) Segura Lecina, O.; Hope, M. A.; Venkatesh, A.; Bjorgvinsdottir, S.; Rossi, K.; Loiudice, A.; Emsley, L.; Buonsanti, R. Colloidal-ALD-Grown Hybrid Shells

Nucleate via a Ligand-Precursor Complex. *J. Am. Chem. Soc.* **2022**, *144* (9), 3998–4008. https://doi.org/10.1021/jacs.1c06777.

(12) Zhang, J.; Zhang, H.; Cao, W.; Pang, Z.; Li, J.; Shu, Y.; Zhu, C.; Kong, X.; Wang, L.; Peng, X. Identification of Facet-Dependent Coordination Structures of Carboxylate Ligands on CdSe Nanocrystals. *J. Am. Chem. Soc.* **2019**, *141* (39), 15675–15683. https://doi.org/10.1021/jacs.9b07836.

(13) Loiudice, A.; Segura Lecina, O.; Bornet, A.; Luther, J. M.; Buonsanti, R. Ligand Locking on Quantum Dot Surfaces via a Mild Reactive Surface Treatment. *J. Am. Chem. Soc.* **2021**, *143* (33), 13418–13427. https://doi.org/10.1021/jacs.1c06777.

(14) Becker-Koch, D.; Albaladejo-Siguan, M.; Lami, V.; Paulus, F.; Xiang, H.; Chen, Z.; Vaynzof, Y. Ligand Dependent Oxidation Dictates the Performance Evolution of High Efficiency PbS Quantum Dot Solar Cells. *Sustain. Energy Fuels* **2019**, *4* (1), 108–115. https://doi.org/10.1039/c9se00602h.

(15) Tang, J.; Brzozowski, L.; Barkhouse, D. A. R.; Wang, X.; Debnath, R.; Wolowiec, R.; Palmiano, E.; Levina, L.; Pattantyus-Abraham, A. G.; Jamakosmanovic, D.; Sargent, E. H. Quantum Dot Photovoltaics in the Extreme Quantum Confinement Regime: The Surface-Chemical Origins of Exceptional Air- and Light-Stability. *ACS Nano* **2010**, *4* (2), 869–878. https://doi.org/10.1021/nn901564q.

(16) Gray, V.; Allardice, J. R.; Zhang, Z.; Dowland, S.; Xiao, J.; Petty, A. J.; Anthony, J. E.; Greenham, N. C.; Rao, A. Direct vs Delayed Triplet Energy Transfer from Organic Semiconductors to Quantum Dots and Implications for Luminescent Harvesting of Triplet Excitons. *ACS Nano* **2020**, *14* (4), 4224–4234. https://doi.org/10.1021/acsnano.9b09339.

(17) Green, P. B.; Yarur Villanueva, F.; Imperiale, C. J.; Hasham, M.; Demmans, K. Z.; Burns, D. C.; Wilson, M. W. B. Directed Ligand Exchange on the Surface of PbS Nanocrystals: Implications for Incoherent Photon Conversion. *ACS Appl. Nano Mater.* **2021**, *4* (6), 5655–5664. https://doi.org/10.1021/acsanm.1c00853.

(18) Hens, Z.; Martins, J. C. A Solution NMR Toolbox for Characterizing the Surface Chemistry of Colloidal Nanocrystals. *Chem. Mater.* **2013**, *25* (8), 1211–1221.

(19) Kessler, M. L.; Starr, H. E.; Knauf, R. R.; Rountree, K. J.; Dempsey, J. L. Exchange Equilibria of Carboxylate-Terminated Ligands at PbS Nanocrystal Surfaces. *Phys. Chem. Chem. Phys.* **2018**, *20* (36), 23649–23655. https://doi.org/10.1039/c8cp04275f.

(20) De Nolf, K.; Cosseddu, S. M.; Jasieniak, J. J.; Drijvers, E.; Martins, J. C.; Infante, I.; Hens, Z. Binding and Packing in Two-Component Colloidal Quantum Dot Ligand Shells: Linear versus Branched Carboxylates. *J. Am. Chem. Soc.* **2017**, *139* (9), 3456–3464. https://doi.org/10.1021/jacs.6b11328.

(21) Cadena, D. M.; Sowa, J. K.; Cotton, D. E.; Wight, C. D.; Hoffman, C. L.; Wagner, H. R.; Boette, J. T.; Raulerson, E. K.; Iverson, B. L.; Rossky, P. J.; Roberts, S. T. Aggregation of Charge Acceptors on Nanocrystal Surfaces Alters Rates of Photoinduced Electron Transfer. *J. Am. Chem. Soc.* **2022**, *144* (49), 22676–22688. https://doi.org/10.1021/jacs.2c09758.

(22) Xia, P.; Raulerson, E. K.; Coleman, D.; Gerke, C. S.; Mangolini, L.; Tang, M. L.; Roberts, S. T. Achieving Spin-Triplet Exciton Transfer between Silicon and Molecular Acceptors for Photon Upconversion. *Nat. Chem.* **2020**, *12* (2), 137–144.

https://doi.org/10.1038/s41557-019-0385-8.

(23) Luo, X.; Lai, R.; Li, Y.; Han, Y.; Liang, G.; Liu, X.; Ding, T.; Wang, J.; Wu, K. Triplet Energy Transfer from CsPbBr3 Nanocrystals Enabled by Quantum Confinement. *J. Am. Chem. Soc.* **2019**, *141* (10), 4186–4190. https://doi.org/10.1021/jacs.8b13180.

(24) Knauf, R. R.; Lennox, J. C.; Dempsey, J. L. Quantifying Ligand Exchange Reactions at CdSe Nanocrystal Surfaces. *Chem. Mater.* **2016**, *28* (13), 4762–4770. https://doi.org/10.1021/acs.chemmater.6b01827.

(25) Kessler, M. L.; Kelm, J. E.; Starr, H. E.; Cook, E. N.; Miller, J. D.; Rivera, N. A.; Hsu-Kim, H.; Dempsey, J. L. Unraveling Changes to PbS Nanocrystal Surfaces Induced by Thiols. *Chem. Mater.* **2022**, *34* (4), 1710–1721. https://doi.org/10.1021/acs.chemmater.1c03888.

(26) He, S.; Han, Y.; Guo, J.; Wu, K. Entropy-Gated Thermally Activated Delayed Emission Lifetime in Phenanthrene-Functionalized CsPbBr3Perovskite Nanocrystals. *J. Phys. Chem. Lett.* **2021**, 8598–8604. https://doi.org/10.1021/acs.jpclett.1c02547.

(27) He, S.; Han, Y.; Guo, J.; Wu, K. Entropy-Powered Endothermic Energy Transfer from CsPbBr 3 Nanocrystals for Photon Upconversion . *J. Phys. Chem. Lett.* **2022**, 1713–1718. https://doi.org/10.1021/acs.jpclett.2c00088.
